# Prevalence and determinants of faecal carriage of carbapenem- and third-generation cephalosporin-resistant Enterobacterales: a cross-sectional household survey in northern Vietnam

**DOI:** 10.1016/j.lanwpc.2024.101281

**Published:** 2025-01-13

**Authors:** Max van Wijk, Hoang Huy Tran, Bich Ngoc Thi Vu, Costanza Tacoli, Tu Cam Thi Nguyen, Quynh Dieu Pham, Thương Hong Thi Nguyen, Trang Thu Nguyen, Hien Anh Thi Nguyen, Tung Son Trinh, Thai Duy Pham, Huong Kieu Thi Tran, Dung Tien Viet Vu, Duc Anh Dang, Tien Dac Tran, Duong Thanh Nguyen, H. Rogier van Doorn, Thomas Kesteman, Sonia Lewycka

**Affiliations:** a Oxford University Clinical Research Unit (OUCRU), National Hospital for Tropical Diseases, 78 Giai Phong, Dong Da District, Hanoi, Vietnam; b Faculty of Pharmacy – University of Tours, 31 Avenue Monge, 37200, Tours, France; c National Institute of Hygiene and Epidemiology (NIHE), 1 Yec Xanh, Hanoi, Pham Dinh Ho, Hai Ba Trung, Vietnam; d Centre for Disease Control, Ha Nam Province, Vietnam; e Department of Health, Ha Nam Province, Vietnam; f Centre of Tropical Medicine and Global Health, Nuffield Department of Medicine, University of Oxford, New Richards Building, Roosevelt Dr, Headington, Oxford, OX3 7LG, United Kingdom

**Keywords:** Antibiotic, Resistance, Third-generation cephalosporin, Carbapenem, Enterobacterales, Commensal, Carriage, Community, Meat, Water, Sanitation and hygiene (WASH), Vietnam

## Abstract

**Background:**

Antimicrobial resistance (AMR) is a silent pandemic causing 1.27 million deaths in 2019, disproportionately affecting low- and middle-income countries, but resistance among commensal microbiota and the determinants of carriage have not been widely reported. This cross-sectional household study aimed to determine the prevalence of carbapenem-resistant (CRE) and third-generation cephalosporin-resistant Enterobacterale*s* (C3GRE) in a rural community in Ha Nam northern Vietnam, as well as the socio-demographic, behavioural, and environmental determinants of carriage.

**Methods:**

1502 individuals across 324 households were surveyed between July 2018 and April 2019. Faecal samples were cultured on meropenem and ceftazidime supplemented media to identify CRE and C3GRE, respectively. Logistic regression models were used to explore risk factors for CRE and C3GRE carriage compared to susceptible strains.

**Findings:**

Colonisation with C3GRE and CRE was 94.0% (95% Confidence Interval (CI) 93.5%–94.4%) and 1.9% (1.6%–2.2%), respectively. The CRE prevalence was too low to explore determinants. Antibiotic use in the last month (adjusted OR 1.22 [95% CI 0.45–3.31]) and recent illness (aOR 1.48 [0.34–6.51]) were not associated with C3GRE carriage. Variables associated with C3GRE carriage were high-income (OR 0.29 [0.12–0.74]), worse sanitary conditions (aOR 4.35 [1.07–17.43]), and frequent beef consumption (aOR 6.56 [2.16–19.98]). A protective association between C3GRE carriage and animal husbandry was observed in children under 5-years (aOR 0.27 [0.09–0.84]). For participants 5-years and older, chicken consumption was associated with increased likelihood of C3GRE carriage (aOR 3.45 [1.45–8.22]), while a protective association was observed for regular tofu (aOR 0.32 [0.14–0.74]) and fermented food consumption (aOR 0.55 [0.31–0.96]).

**Interpretation:**

In this high-prevalence setting, colonisation with C3GRE was not associated with individual antibiotic use, while environmental exposures, including food and sanitary conditions, were associated with C3GRE colonisation. Further research is required to understand the mechanisms behind these associations.

**Funding:**

This work was supported by 10.13039/501100000769Oxford University Clinical Research Unit internal grants in Vietnam from the 10.13039/100010269Wellcome Trust Africa Asia Programme core grants (2015-2022—106680/Z/14/Z, and 2022-2029—225167/Z/22/Z).

**Research in context**

**Evidence before this study**

A targeted literature review was initially performed in PubMed in February and March 2021 and updated in May 2024 to identify peer-reviewed publications that describe the carriage of third-generation cephalosporin- and/or carbapenem-resistance in various settings and countries, with a particular focus on community studies in Vietnam and the wider region of southeast Asia. We found no evidence of good quality that described resistance rates in the community in Vietnam. One meta-analysis by Xu et al. reviewed 61 studies and estimated the overall community-prevalence of carbapenem-resistant Enterobacterales to be low in Asia (less than 1.0%), with a significantly higher rate in Vietnam (3.0%). Another meta-analysis by Malchione et al. reported similar resistance rates across Asia, but again higher levels in Vietnam. National surveillance data published by Vu et al. from 13 hospitals in Vietnam suggests high levels of third-generation cephalosporin-resistance (e.g., over 70% among *E. coli* isolates). Hospital based AMR surveillance relies on clinical isolates, but less was known before this study about the prevalence of third-generation cephalosporin- and carbapenem-resistance among the general population, as well as the determinants for colonization with resistant bacteria in the community in Vietnam.

**Added value of this study**

Our study confirms high levels of resistance to third-generation cephalosporins in commensal Enterobacterales in Vietnamese communities, and identifies specific factors associated with higher likelihood of colonisation. Individual antibiotic use was not associated with higher likelihood of colonisation, but water sanitation and hygiene (WASH) and consumption of animal products were. The results i) confirm that third-generation cephalosporin-resistant Enterobacterales rates are also high in rural communities in Vietnam, ii) provide evidence that carbapenem-resistant Enterobacterales have not (yet) widely spread to the community in Vietnam, and iii) expand our knowledge on potential risk factors for colonization with resistant bacteria in communities of low- and middle-income countries.

**Implications of all the available evidence**

While interventions to reduce the high levels of antibiotic use in communities and in livestock production are urgently needed, further research is also required to understand the mechanisms behind the observed effects in this study. In particular, we need to understand possible sources of occult exposure to antibiotics through residues in animal food products, as well as possible sources of bacterial contamination from the environment, such as through water, animal food products, and other foods. Resistance profiling and phylogenetic analysis would help to identify likely sources of resistance in human commensal bacteria, and transmission between humans, animals, and the environment. The findings from the current study can inform the development of interventions and the design of large-scale population-based trials targeting AMR in similar settings.

## Introduction

Antimicrobial resistance (AMR) caused 1.27 million deaths in 2019 and represents a growing treatment challenge for bacterial infections worldwide.^1^ Antibiotics are vital for the treatment of bacterial infections, but antibiotics are often used inappropriately for the prevention and treatment of infectious diseases as well as for livestock management. Southeast Asia is regarded as a global hotspot for the emergence and spread of AMR, but there is a lack of systematic data collection from the region.^2^

Enterobacterales belong to the commensal gut microbiota but are also a common cause of community- and healthcare-acquired infections. These infections are often treated with β-lactam antibiotics, such as third-generation cephalosporins. However, Enterobacterale*s* can become resistant to this class of antibiotics, often by producing extended-spectrum β-lactamases (ESBLs).^3^ National surveillance data from 13 hospitals in Vietnam demonstrated high and increasing levels of third-generation cephalosporin-resistant *Escherichia coli* (71.7%) and *Klebsiella pneumoniae* (55.6%) in 2016–2017.^4^^,^^5^ Carbapenems, a class of broad-spectrum antibiotics, are considered a last resort option to treat resistant Enterobacterale*s*. Worryingly, carbapenem-resistant Enterobacterales (CRE) are now abundantly found in clinics around the world, particularly in the Mediterranean, the Middle-East, and in Asia.^6^ CRE are more prevalent in Vietnam than in most Southeast Asian countries^7^ and have emerged as a major cause of hospital-acquired infections throughout the country.^8^ The rapid emergence of third generation cephalosporin resistant Enterobacterales (C3GRE) and CRE is also associated with increased medical expenses,^9^ which is of particular concern for LMICs.

World Health Organization (WHO) GLASS AMR surveillance relies on clinical isolates and is mostly hospital-based, but less is known about the prevalence of C3GRE and CRE in human commensal microbiota in the community and the determinants associated with this colonization in the general population in LMICs. Limiting the unnecessary and inappropriate use of antimicrobials is the cornerstone of antimicrobial stewardship strategies,^10^ though transmission from environmental sources may also play an important role in colonization. Other factors previously identified as risk factors for the carriage of resistant Enterobacterales include food,^10^ living in high-density areas,^11^ poor living conditions,^12^ and healthcare exposure.^13^^,^^14^ In Southeast Asia, the community-carriage of ESBL-producing Enterobacterales has previously been associated with consumption of undercooked meat in Thailand,^15^ and exposure to animal manure and raw meat products in Cambodia.^16^

A better understanding of community behaviour related to CRE and C3GRE colonization is the first step to identifying risk factors and may facilitate the design of interventions that mitigate AMR in the community. In line with these objectives, the present household study aimed (i) to provide prevalence estimates of third-generation cephalosporin- (C3GRE) and carbapenem-resistant Enterobacterales (CRE) in a rural community in northern Vietnam, and (ii) to explore the behavioural and environmental determinants that are associated with carriage. These findings will also inform the development of interventions and the design of large-scale population-based trials targeting AMR in similar settings.

## Methods

### Study population

**Fig. 1:**
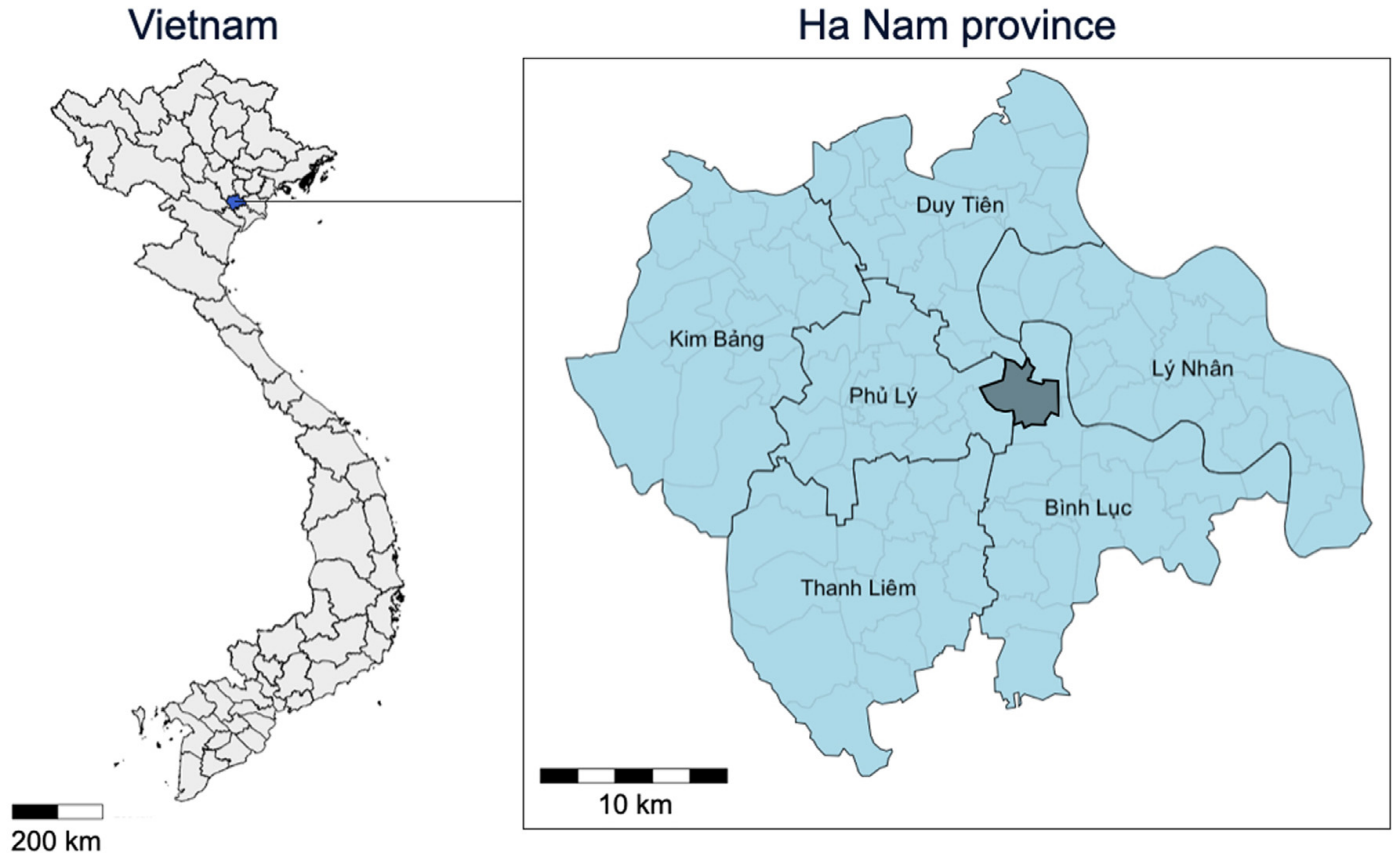
Spatial visualization of the location of Binh Luc district in Ha Nam province, northern Vietnam.

Between 16 July 2018 and 10 April 2019, a total of 1868 individuals from 389 households were randomly selected from a list of registered households provided by the local health authority for 19 villages in one commune of Binh Luc District, Ha Nam province, northern Vietnam. All residents of selected households were invited to participate (see Fig. 1). No further eligibility criteria were applied. This limited the possibility of selection bias.

A cross-sectional household survey was designed (Supplementary File 1) and the primary caregiver of each household was interviewed in Vietnamese by trained interviewers. Sociodemographic characteristics, recent illnesses, antibiotic use, and healthcare exposures were collected for every household member. General socioeconomic information (e.g., direct observations of assets and house construction), handwashing practices, dietary habits, animal husbandry, and knowledge on antibiotics and antibiotic resistance were recorded at the household level. We also captured the birth history, vaccination coverage, and breastfeeding practices of children under the age of 5. Questions were based on standard Demographic and Health Survey tools,^17^ with the addition of more detailed questions on antibiotic knowledge and use, knowledge of AMR, household food consumption, and sources of health information. Questionnaires were reviewed by experts, piloted, and revised to ensure content and face validity.

**Fig. 2:**
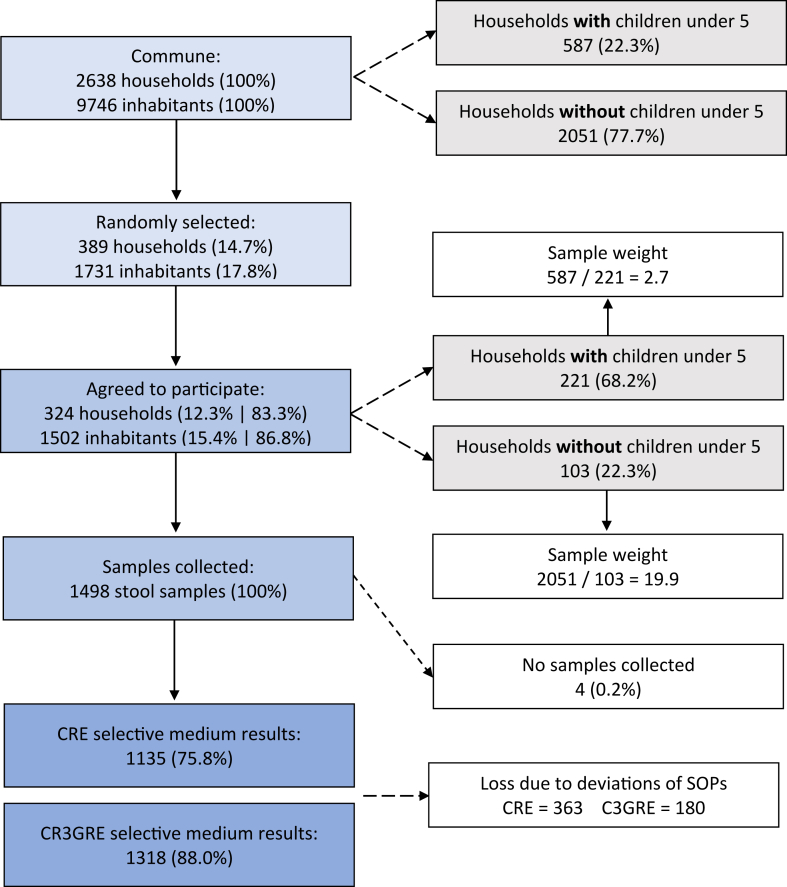
Study design and flow diagram of participant recruitment and faecal sample collection. Abbreviations: C3GRE (third-generation cephalosporin-resistant Enterobacterales), CRE (carbapenem-resistant Enterobacterales), SOP (standard operating procedure).

We oversampled households with children under 5 years-of-age. Sampling weights were calculated based on the total number of households within the commune, with or without children under 5, and incorporated in the analyses to correct for study design (see Fig. 2).

### Data and sample collection

Household members were requested to self-collect faeces using faeces collection containers provided at the time of the interview. Samples were collected the day after the interview and shipped to Hanoi at 2–8 °C within 24 h.

### Ethics approval and consent to participate

The study was approved by Oxford Tropical Research Ethics Committee (Reference 552-17), the Institutional Review Board in Bio-medical Research of the National Institute of Hygiene & Epidemiology (Reference IRB-VN0105 7-01/2018) and the Ethics Committee of London School of Hygiene & Tropical Medicine (Reference 15831). Written informed consent was collected from all household members aged 18-years or older or parent/legal guardian of those under 18-years, and signed assent from those aged 12 to 18 years.

### Isolation and culture of Enterobacterales on selective media

We used a direct plating method similar to the WHO Tricycle protocol (for One Health surveillance in low-resource settings), which was designed to be a relatively cheap and simple approach that could be used to assess resistance patterns across a large number of samples.^18^^,^^19^ Stool samples were directly plated on two MacConkey (MC) agar plates (MacConKey agar No. 3, Code CM0115, Oxoid, UK^20^), one supplemented with ceftazidime (2.0 mg/L) and one with meropenem (0.5 mg/L), using a sterile inoculating loop. Plates were incubated overnight at 37 °C. Colonization with CRE and C3GRE was defined as the growth of lactose-fermenting pink large smooth colonies on the meropenem and ceftazidime supplemented MC plate, respectively.^19^^,^^21^ Quality control (QC) for MC agar plates followed the technical guidance from Oxoid, which required checking the plates (for each batch of media) with positive controls. These were: *Escherichia coli* ATCC 25922 ∗ (Good growth; red colonies with bile precipitation) and *Shigella sonnei* ATCC 25931 ∗ (Good growth; straw coloured colonies). The negative control was *Enterococcus faecalis* ATCC 29212 ∗ (Inhibited).

### Data transformation

Microsoft Excel and Rstudio V1.4.1106^22^ were used for data curation and statistical analyses. The socioeconomic condition of the household was calculated based on a principal component analysis (PCA) of household assets (floor, roof, and wall materials, crowding (number of people per sleeping room), ownership of electricity, radio, television, telephone, mobile phone, refrigerator, bed, table and chair set, sofa, computer, tablet or iPad, fan, air conditioner, gas cooker, electric cooker, washing machine, bicycle, motorcycle, tractor, car or truck, ship or boat) and subdivided into three wealth terciles. The household's WASH condition was based on three factors: (1) using rainwater as a source of drinking water, (2) boiling water before consumption, and (3) having a flush toilet. Households were considered to have a ‘better’ WASH condition when they met these three criteria, ‘middle’ was assigned to households where one of these criteria were missing, and ‘worse’ for the remaining households with one or none of these conditions. Details of all curated variables can be found in Supplementary File 2.

### Statistical analysis

The total sample size for this study was determined for a different aspect of the study,^19^ but we estimated that with 1500 participants in 324 households we would have a margin of error of between 2.7% and 3.7% to estimate prevalence, using the most conservative value of 50%, a 95% confidence level, and a design effect of between 1.18 and 2.09 (based on intracluster correlation coefficient (ICC) in the range of 0.05–0.30 and average household size of 4.64).

All analyses were adjusted for survey design using the R svyr package (which computes robust standard errors to adjust for correlation within households and applies sampling weights).^23^ Logistic regression models were used to explore the association between factors and C3GRE or CRE colonization. Analyses were carried out in 4 levels: 1) CRE on a household level (comparing households with and without at least one member colonised with CRE), 2) C3GRE on an individual level, 3) C3GRE on an individual level stratified for member under and over the age of 5, and 4) C3GRE on an individual level for factors that were only recorded for children under 5 years-of-age. Models were adjusted for age and economic status as possible confounders. Mediation analysis was conducted to explore the possible mediating roles of antibiotic use in the last 4 weeks, recent illness, and household's WASH condition on the effects of other factors on C3GRE.^24^

### Role of the funding source

The funders had no role in study design, data collection, data analysis, interpretation, or writing of the report.

## Results

### Sample description

A total of 324 households (83.9% of invited households) agreed to participate, accounting for 1502 individuals (80.4% of invited individuals). Faecal samples and survey responses were collected from 1498 participants. Most samples were successfully cultured to test for C3GRE (1318, 88.0%) and CRE (1135, 75.7%). A total of 24.2% (363 out of 1498) CRE and 12.0% (180 out of 1498) C3GRE culture results were invalid and could not be re-tested (see Fig. 2). The ICC was 0.08 for C3GRE and 0.23 for CRE. No data were missing on explanatory variables or variables used for adjusting (age, SES, WASH, and antibiotic use), so models for each exposure variable had the same number of records.

The median age of the study population was 29 years, and 246 (16.4%) participants were under 5 years-of-age. The male to female sex ratio was 0.99. Of the 503 children under 17 years-of-age, half (48.5%) were attending school. Most adults were farmers (49.7%), followed by manual labourers (18.0%) and factory workers (17.7%). Most adults only received lower secondary education (56.9%), followed by no education (15.7%) and upper secondary education (15.5%). There were minimal differences between participants for whom C3GRE and CRE results were available and the whole study population (see Table 1).

**Table 1:** Sociodemographic description of complete study population, and C3GRE and CRE subsets.

	Complete dataset *n = 1498*	C3GRE dataset *n = 1318*	CRE dataset *n = 1135*
**Age**			
Mean ± std	30.0 ± 21.9	30.2 ± 22.1	30.2 ± 22.0
Median [IQR]	29 [10–48]	29 [10–48]	29 [10–48]
**Age**			
<5 years	16.4% (246)	16.1% (183)	16.0% (211)
5–19 years	19.4% (290)	19.6% (223)	19.9% (262)
20–29 years	16.2% (242)	15.6% (177)	15.3% (201)
30–39 years	17.9% (268)	17.8% (202)	18.1% (238)
40–49 years	8.1% (121)	8.5% (97)	8.4% (111)
50–59 years	11.3% (169)	11.5% (131)	12.0% (158)
≥60 years	10.8% (162)	10.7% (122)	10.4% (137)
**Sex**			
Female	51.8% (776)	51.8% (588)	52.0% (685)
Male	48.2% (722)	48.2% (547)	48.0% (633)
**Household wealth**			
Low-income	34.0% (510)	35.2% (399)	34.4% (453)
Middle-income	32.6% (489)	29.0% (329)	32.1% (423)
High-income	33.3% (499)	35.9% (407)	33.5% (442)
**Occupation**^a^			
Farmer	49.7% (495)	50.2% (379)	50.8% (444)
Labourer	18.0% (179)	17.5% (132)	17.4% (152)
Factory worker	17.7% (176)	18.1% (137)	17.3% (151)
Office worker	2.7% (27)	3.3% (25)	3.0% (26)
Shop/retail/hospitality	2.1% (21)	1.3% (10)	1.8% (16)
Other work	1.5% (15)	1.7% (13)	1.5% (13)
Student	0.9% (9)	0.8% (6)	0.9% (8)
No work/unknown	7.3% (73)	7.0% (53)	7.3% (64)
**Education**^a^			
College/university	4.5% (45)	5.2% (39)	4.9% (43)
Professional school	0.3% (3)	0.1% (1)	0.2% (2)
Upper secondary	15.5% (154)	16.0% (121)	15.7% (137)
Lower secondary	56.9% (566)	56.3% (425)	56.9% (497)
Primary and lower	4.1% (41)	4.5% (34)	4.2% (37)
No education	15.7% (156)	15.2% (115)	15.4% (135)
Unknown	3.0% (30)	2.6% (20)	2.6% (23)
**Participants ≤16 years-of-age**			
In school	48.5% (244)	45.3% (172)	48.9% (217)
Attended school before	33.6% (169)	37.1% (141)	34.5% (153)
Never attended school	17.9% (90)	17.6% (67)	16.7% (74)

a Stratified analysis for all individuals >16 years-of-age only, and proportions do therefore not represent the complete sample.

### Household colonization with carbapenem-resistant Enterobacterales (CRE)

CRE were found in 17 out of 1135 faecal samples, resulting in an overall weighted prevalence of 1.9% (95% CI: 1.6%–2.2%). Of these 17 CRE carriers, 5 were children under 5 years-of-age (U5) and 12 were 5 years or older (O5). The overall CRE colonization was 2.7% (95% CI: 1.0%–6.4%) among U5, and 1.3% (95% CI: 0.7%–2.2%) among O5. The prevalence was not significantly different between these two age groups (Fisher exact test, p = 0.170). No CRE carriers were detected in 13 out of the 19 villages surveyed (68.4%) and more than half (10/17, 58.8%) of all CRE carriers were living in one village. This village had a CRE carriage of 12.2% (95% CI 6.6%–21.2%), whereas the prevalence in the remaining 5 villages ranged between 1.3% and 4.6%, with a mean of 2.6% (see Supplementary File 3).

Given the small number of isolates, analyses for CRE were performed on a household level. Households were considered CRE positive when at least one member was colonized with CRE, resulting in 12 CRE positive households. Most CRE-positive households (10 out of 12, 83.3%) were sampled between July and September 2018. Almost all (11 out of 12, 91.7%) CRE-positive households owned animals, but the CRE prevalence was not significantly different from households that did not own animals. None of the 12 CRE-positive households reported any member exposed to a hospital in the last month. Illness and antibiotic use within the household were not associated with CRE carriage (Table 2). A complete overview of all analysed factors can be found in supplementary file 4.

**Table 2:** Unadjusted and adjusted analysis of CRE colonisation on a household level (weighted).

Variable	Total n = 245	CRE positive	Crude OR	p-value^a^	OR adjusted for SES	p-value^a^
**Socio-economic status (SES)**						
Low- and middle-income	65.7% (161)	1.9% (3)	ref			
High-income	34.3% (84)	10.7% (9)	3.93 [0.76–20.35]	0.105		
**AMU 4 weeks**						
No and unknown	66.1% (162)	5.6% (9)	ref		ref	
Yes	33.9% (83)	3.6% (3)	0.54 [0.09–3.37]	0.506	0.61 [0.09–4.07]	0.609
**Illness**^b^						
No and unknown	74.7% (183)	5.5% (10)	ref			
Yes	25.3% (62)	3.2% (2)	0.79 [0.11–5.75]	0.812	1.02 [0.12–8.68]	0.983
**Sampling period**						
Period 1 (July–September)	49.8% (122)	8.2% (10)	ref		ref	
Period 2 (November–December)	9.0% (22)	4.5% (1)	0.13 [0.01–1.14]	0.067	0.10 [0.01–0.97]	**0.048∗**
Period 3 (March–April)	41.2% (101)	1.0% (1)	0.25 [0.03–2.18]	0.210	0.26 [0.03–2.27]	0.224
**Owning animals**						
No	44.1% (108)	0.9% (1)	ref		ref	
Yes	55.9% (137)	8.0% (11)	4.52 [0.52–39.32]	0.172	4.48 [0.52–38.44]	0.173
**Antibiotic use in animals**^c^**(n = 137)**						
No	17.1% (42)	11.9% (5)	ref		ref	
Yes	38.8% (95)	6.3% (6)	0.18 [0.03–1.01]	0.053	0.18 [0.03–1.00]	0.051
**Owning livestock**						
No livestock	49.8% (122)	1.6% (2)	ref		ref	
1 livestock species	30.6% (75)	9.3% (7)	3.56 [0.62–20.60]	0.157	3.65 [0.64–20.86]	0.147
2 livestock species or more	19.6% (48)	6.3% (3)	0.56 [0.09–3.54]	0.539	0.62 [0.10–3.82]	0.610
**Beef consumption**						
Less than once per week	66.5% (163)	4.3% (7)	ref		ref	
Once per week	15.1% (37)	5.4% (2)	2.01 [0.50–16.11]	0.510	1.56 [0.21–11.32]	0.663
More than once per week	18.4% (45)	6.7% (3)	1.83 [0.27–12.56]	0.540	1.52 [0.24–9.48]	0.657
**Chicken consumption**						
Less than once per week	61.2% (150)	3.3% (5)	ref		ref	
Once per week	14.3% (35)	2.9% (1)	1.62 [0.16–16.13]	0.681	1.53 [0.16–14.59]	0.712
More than once per week	24.5% (60)	10.0% (6)	1.58 [0.29–8.48]	0.594	1.83 [0.33–10.06]	0.487
**WASH condition**^d^						
Better condition	35.1% (86)	5.8% (5)	ref		ref	
Middle or worse condition	64.9% (159)	4.4% (7)	0.23 [0.05–1.08]	0.063	0.22 [0.05–1.04]	0.058
**Tofu consumption**						
Once per week and less	24.5% (60)	8.3% (5)	ref		ref	
More than once per week	75.5% (185)	3.8% (7)	0.42 [0.09–2.08]	0.291	0.36 [0.07–1.83]	0.220
**Fermented food consumption**						
Once per week and less	69.0% (169)	5.9% 910)	ref		ref	
More than once per week	31.0% (76)	2.6% (2)	0.59 [0.08–4.31]	0.607	0.56 [0.08–3.94]	0.562
**Drinking water source**						
Improved source	64.1% (157)	4.5% (7)	ref		ref	
Rainwater	35.9% (88)	5.7% (5)	3.96 [0.85–18.47]	0.081	3.98 [0.85–18.65]	0.081
**Washing hands combined**						
Not often	52.2% (128)	3.1% (4)	ref		ref	
Often	47.8% (117)	6.8% (8)	1.24 [0.27–5.76]	0.787	0.83 [0.14–5.09]	0.840

a Significance as ∗ at p-value <0.05, ∗∗ at p-value <0.01, and ∗∗∗ at p-value <0.001.

b Households were considered as having illness when at least one member reported to have diarrhoea, cough, or fever in the last 2 weeks.

c Analysis only for individuals that owned animals including livestock and domestic animals (n = 137).

d Better condition if households used 1) only rain water for drinking, 2) always boiled water before consuming, and 3) had a flushed toilet. Middle if one was missing, and worse if two were missing.

### Determinants for and colonization with C3GRE

C3GRE were found in 1233 of 1318 faecal samples, resulting in an overall weighted prevalence of 94.0% (95% CI: 93.5%–94.4%). Children U5 were less likely to be colonized with C3GRE (88.6%, 95% CI: 83.6%–92.3%) compared to individuals O5 (94.5%, 95% CI: 93.0%–95.7%), but this difference was not significant after incorporating the sampling weights. Participants who were living in a high-income household (92.8%, 95% CI: 89.9%–94.9%) were less likely to carry C3GRE compared to low-income households (95.8%, 95% CI: 93.5%–97.3%), this difference was statistically significant after incorporating the sampling weights (see Table 3).

**Table 3:** Unadjusted and adjusted analysis of C3GRE colonisation on an individual level (weighted).

Variable	Proportion (n) n = 1318	C3GRE positive	Crude OR	p-value^a^	OR adjusted for age and SES	p-value^a^	OR adjusted for age, SES, and WASH	p-value^a^	OR adjusted for age, SES, and antibiotic use	p-value^a^
**Age**										
≥5 years-of-age	84.0% (1107)	94.5% (1046)	ref							
<5 years-of-age	16.0% (211)	88.6% (187)	0.69 [0.37–1.28]	0.240						
**Socio-economic status (SES)**										
Low income	34.4% (453)	95.8% (434)	ref							
Middle income	32.1% (423)	92.0% (389)	0.46 [0.20–1.05]	0.066						
High income	33.5% (442)	92.8% (410)	0.29 [0.12–0.74]	**0.010∗**						
**WASH condition**^b^										
Better condition	39.0% (514)	90.9% (467)	ref		ref					
Middle condition	42.3% (557)	94.3% (525)	1.51 [0.63–3.67]	0.358	1.78 [0.77–4.12]	0.180				
Worse condition	18.7% (247)	97.6% (241)	3.96 [1.07–14.61]	**0.040∗**	4.35 [1.07–17.43]	**0.039∗**				
**Antibiotic use 4 weeks**										
No and unknown	87.3% (1150)	93.6% (1076)	Ref		ref		ref			
Yes	12.7% (168)	93.5% (157)	1.15 [0.44–3.02]	0.774	1.22 [0.45–3.31]	0.696	1.42 [0.48–4.22]	0.531		
**Sex**										
Female	52.0% (685)	94.2% (645)	ref		ref		ref		ref	
Male	48.0% (633)	92.0% (588)	0.80 (0.38–1.69)	0.559	0.81 (0.39–1.72)	0.591	0.84 (0.39–1.78)	0.646	0.82 (0.38–1.75)	0.608
**Illness**^c^										
No and unknown	92.2% (1215)	93.4% (1135)	ref		ref		ref		ref	
Yes	7.8% (103)	95.1% (98)	1.45 [0.34–6.21]	0.616	1.48 [0.34–6.51]	0.603	1.53 [0.33–7.02]	0.583	1.43 [0.33–6.17]	0.631
**Sampling period**										
Period 1 (July–September)	48.1% (634)	93.1% (590)	ref		ref		ref		ref	
Period 2 (November–December)	15.3% (201)	94.5% (190)	1.59 [0.56–4.47]	0.385	1.50 [0.51–4.38]	0.462	1.60 [0.56–4.51]	0.379	1.48 [0.50–4.41]	0.481
Period 3 (March–April)	36.6% (483)	93.8% (453)	0.85 [0.37–1.97]	0.706	0.79 [0.34–1.84]	0.589	0.85 [0.35–2.05]	0.723	0.78 [0.33–1.86]	0.580
**Owning animals**										
No	40.1% (528)	95.8% (506)	ref		ref		ref		ref	
Yes	59.9% (790)	92.0% (727)	0.79 [0.33–1.87]	0.596	0.86 [0.37–2.00]	0.729	0.90 [0.39–2.05]	0.794	0.87 [0.36–2.08]	0.758
**Antibiotic use in animals**^d^**(n = 790)**										
No	18.5% (244)	93.9% (229)	ref		ref		ref		ref	
Yes	41.4% (546)	91.2% (498)	0.44 [0.17–1.14]	0.091	0.40 [0.14–1.17]	0.096	0.46 [0.14–1.55]	0.213	0.40 [0.14–1.15]	0.090
**Owning livestock**										
No livestock	46.8% (617)	95.8% (591)	ref		ref		ref		ref	
1 livestock species	31.2% (411)	93.7% (385)	0.87 [0.34–2.20]	0.769	0.90 [0.36–2.24]	0.825	1.01 [0.42–2.43]	0.985	0.91 [0.37–2.26]	0.838
2 livestock species or more	22.0% (290)	88.6% (257)	0.93 [0.39–2.25]	0.877	0.92 [0.39–2.18]	0.858	1.05 [0.48–2.29]	0.906	0.94 [0.40–2.20]	0.881
**Owning pigs**										
No	81.8% (1078)	94.8% (1022)	ref		ref		ref		ref	
Yes	18.2% (240)	87.9% (211)	0.85 [0.41–1.76]	0.668	0.83 [0.39–1.74]	0.616	0.98 [0.49–1.96]	0.947	0.83 [0.40–1.75]	0.632
**Beef consumption**										
Less than once per week	66.2% (872)	92.8% (809)	ref		ref		ref		ref	
Once per week	14.6% (193)	93.3% (180)	0.92 [0.24–3.49]	0.903	1.28 [0.36–4.48]	0.703	1.15 [0.32–4.07]	0.832	1.27 [0.37–4.35]	0.710
More than once per week	19.2% (253)	96.4% (244)	4.36 [1.53–12.43]	**0.006∗∗**	6.56 [2.16–19.98]	**0.001∗∗**	5.48 [1.66–18.06]	**0.006∗∗**	6.58 [2.16–19.99]	**0.001∗∗**
**Chicken consumption**										
Less than once per week	60.8% (802)	92.4% (741)	ref		ref		ref		ref	
Once per week	12.7% (167)	91.0% (152)	0.65 [0.19–2.23]	0.494	0.62 [0.19–2.00]	0.422	0.55 [0.16–1.84]	0.333	0.62 [0.19–2.00]	0.422
More than once per week	26.5% (349)	97.4% (340)	2.60 [0.95–7.11]	0.064	2.74 [0.95–7.90]	0.063	2.18 [0.74–6.45]	0.159	2.75 [0.96–7.92]	0.061
**Tofu consumption**										
Once per week and less	23.8% (313)	96.2% (301)	ref		ref		ref		ref	
More than once per week	76.3% (1005)	92.7% (932)	0.58 [0.24–1.39]	0.222	0.60 [0.24–1.50]	0.277	0.69 [0.27–1.74]	0.431	0.60 [0.24–1.51]	0.284
**Fermented food consumption**										
Once per week and less	67.2% (886)	94.1% (834)	ref		ref		ref		ref	
More than once per week	32.8% (432)	92.4% (399)	1.01 [0.49–2.09]	0.972	0.96 [0.49–1.89]	0.901	1.12 [0.58–2.15]	0.741	0.95 [0.48–1.89]	0.889
**Drinking water source**^e^										
Improved source	59.9% (789)	95.2% (751)	ref		ref				ref	
Rainwater	40.1% (529)	91.1% (482)	0.59 [0.26–1.33]	0.201	0.52 [0.24–1.15]	0.107			0.51 [0.23–1.16]	0.109
**Washing hands combined**										
Not often	52.3% (689)	92.6% (638)	ref		ref		ref		Ref	
Often	47.7% (629)	94.6% (595)	0.96 [0.42–2.21]	0.926	1.37 [0.62–3.07]	0.439	1.02 [0.49–2.11]	0.960	1.41 [0.60–3.30]	0.430

a Significance as ∗ at p-value <0.05, ∗∗ at p-value <0.01, and ∗∗∗ at p-value <0.001.

b Better condition if households used 1) only rain water for drinking, 2) always boiled water before consuming, and 3) had a flushed toilet. Middle if one was missing, and worse if two were missing.

c Participants were considered as having illness when reported to have diarrhoea, cough, or fever in the last 2 weeks.

d Analysis only for individuals that owned animals including livestock and domestic animals (n = 790).

e Not adjusted for WASH, since drinking water source was one of the factors used to determine the WASH condition.

Based on weighted bivariable logistic regression analyses of all 1318 individuals, C3GRE colonization was significantly associated with frequent beef consumption. Participants who reported consuming beef more than once per week were more likely to be colonised with C3GRE than those who consumed beef less than once per week (aOR 6.56, 95% CI: 2.16–19.98). Participants of households with worse water, sanitation, and hygiene (WASH) conditions were more likely to carry C3GRE than individuals of households with a better WASH score (aOR of 4.35, 95% CI: 1.07–17.43). Only 5 participants reported hospital exposure within the last month, and all were colonised with C3GRE. There was no difference in prevalence between the three sampling periods.

Self-reported antibiotic use in the last month was not associated with an increased risk of C3GRE faecal carriage (adjusted OR 1.22 [95% CI 0.45–3.31]).

The addition of WASH conditions as a mediating factor had a stronger effect on the associations than antibiotic exposure or illness. As an example, the aOR of beef consumption changed from 6.56 to 5.48 after incorporating WASH but became 6.58 and 6.50 upon incorporating antibiotic use and recent illness, respectively. A complete overview of all analysed factors can be found in Supplementary File 5.

### Differences in determinants for C3GRE between U5 and O5

As described before, the proportion of individuals colonised with C3GRE was significantly different between U5 and O5 (88.6% and 94.5%, respectively). Stratified analyses were performed to examine which factors are associated with C3GRE carriage per subset (Table 4).

**Table 4:** Univariable and adjusted analyses of C3GRE colonisation stratified by individuals older (O5) and younger than 5 years-of-age (U5) (unweighted).

Variable	Adults and children aged 5 and above (n = 1107)				Children under 5 (n = 211)			
	Proportion (n)	C3GRE positive	OR adjusted for SES	p-value^a^	Proportion (n)	C3GRE positive	OR adjusted for SES	p-value^a^
**Socio-economic status (SES)**								
Low income	34.9% (386)	96.1% (371)			31.8% (67)	94.0% (63)		
Middle income	32.1% (355)	93.2% (331)			32.2% (68)	85.3% (58)		
High income	33.1% (366)	94.0% (334)			36.0% (76)	86.8% (66)		
**Antibiotic use 4 weeks**								
No	90.6% (1003)	94.3% (946)	ref		69.7% (147)	88.4% (130)	ref	
Yes	9.4% (104)	96.2% (100)	1.50 [0.54–4.21]	0.441	30.3% (64)	89.1% (57)	1.11 [0.46–2.68]	0.813
**Illness**^b^								
No	93.9% (1040)	94.5% (983)	ref		82.9% (175)	86.9% (152)	ref	
Yes	6.1% (67)	94.0% (63)	0.92 [0.34–2.55]	0.880	17.1% (36)	97.2% (35)	5.32 [0.67–42.01]	0.115
**Sex**								
Female	53.1% (588)	95.2% (560)			46.0% (97)	87.6% (85)	ref	
Male	46.9% (519)	93.6% (486)	0.85 (0.37–1.97)	0.704	54.0% (114)	89.5% (102)	1.20 (0.51–2.82)	0.676
**Sampling period**								
Period 1 (July–September)	47.3% (524)	93.5% (490)	ref		52.1% (110)	90.9% (100)	ref	
Period 2 (November–December)	15.6% (173)	95.4% (165)	1.43 [0.55–3.75]	0.469	13.3% (28)	89.3% (25)	0.95 [0.25–3.57]	0.935
Period 3 (March–April)	37.0% (410)	95.4% (391)	1.47 [0.77–2.78]	0.240	34.6% (73)	84.0% (62)	0.56 [0.20–1.54]	0.262
**Owning animals**								
No	39.8% (441)	95.9% (423)	ref		41.2% (87)	95.4% (83)	ref	
Yes	60.2% (666)	93.5% (623)	0.64 [0.33–1.24]	0.190	58.8% (124)	83.9% (104)	0.27 [0.09–0.84]	**0.025**
**Antibiotic use in animals**^c^								
No	18.6% (206)	96.6% (199)	ref		18.0% (38)	78.9% (30)	ref	
Yes	41.6% (460)	92.2% (424)	0.39 [0.16–0.98]	**0.046∗**	40.8% (86)	86.0% (74)	1.55 [0.52–4.66]	0.434
**Owning livestock**								
No livestock	46.5% (515)	95.9% (494)	ref		48.3% (102)	95.1% (97)	ref	
1 livestock species	31.2% (345)	94.2% (325)	0.71 [0.36–1.43]	0.343	31.3% (66)	90.9% (60)	0.55 [0.16–1.94]	0.357
2 livestock species or more	22.3% (247)	91.9% (227)	0.50 [0.25–1.01]	0.053	20.4% (43)	69.8% (30)	0.12 [0.04–0.39]	**<0.001∗∗∗**
**Owning pigs**								
No	81.5% (902)	95.5% (861)	ref		83.4% (176)	91.5% (161)	ref	
Yes	18.5% (205)	90.2% (185)	0.45 [0.25–0.79]	**0.006∗∗**	16.6% (35)	74.3% (26)	0.29 [0.11–0.76]	**0.013∗**
**Beef consumption**								
Less than once per week	67.6% (748)	93.9% (702)	ref		58.8% (124)	86.3% (107)	ref	
Once per week	14.0% (155)	94.2% (146)	1.13 [0.50–2.57]	0.767	18.0% (38)	89.5% (34)	1.39 [0.44–4.41]	0.576
More than once per week	18.4% (204)	97.1% (198)	2.47 [0.89–6.88]	0.085	23.2% (49)	93.9% (46)	2.92 [0.69–12.37]	0.148
**Chicken consumption**								
Less than once per week	61.4% (680)	93.5% (636)	ref		57.8% (122)	86.1% (105)	ref	
Once per week	12.5% (138)	92.0% (127)	0.79 [0.35–1.77]	0.568	13.7% (29)	86.2% (25)	0.94 [0.27–3.28]	0.927
More than once per week	26.1% (289)	97.9% (283)	3.45 [1.45–8.22]	**0.006∗∗**	28.4% (60)	95.0% (57)	3.41 [0.96–12.09]	0.059
**Tofu consumption**								
Once per week and less	23.7% (262)	97.7% (256)	ref		24.2% (51)	88.2% (45)	ref	
More than once per week	76.3% (845)	93.7% (638)	0.32 [0.14–0.74]	**0.009∗∗**	75.8% (160)	88.8% (142)	0.93 [0.31–2.78]	0.891
**Fermented food consumption**								
Once per week and less	67.6% (748)	95.6% (715)	ref		65.4% (138)	86.2% (119)	ref	
More than once per week	32.4% (359)	92.2% (331)	0.55 [0.31–0.96]	**0.037∗**	34.6% (73)	93.2% (68)	2.11 [0.74–6.03]	0.450
**WASH condition**^d^								
Better condition	39.4% (437)	91.8% (401)	ref		36.5% (77)	85.7% (66)	ref	
Middle condition	42.2% (467)	95.7% (447)	2.11 [1.11–4.00]	**0.023∗**	42.7% (90)	86.7% (78)	1.13 [0.40–3.21]	0.814
Worse condition	18.3% (203)	97.5% (198)	3.51 [1.35–9.17]	**0.011∗**	20.9% (44)	97.7% (43)	7.18 [0.71–72.68]	0.097
**Drinking water source**								
Improved source	59.3% (656)	96.2% (631)	ref		63.0% (133)	90.2% (120)	ref	
Rainwater	40.7% (451)	92.0% (415)	0.44 [0.25–0.79]	**0.007∗∗**	37.0% (78)	85.9% (67)	0.65 [0.22–1.90]	0.436
**Washing hands combined**								
Not often	53.4% (591)	93.4% (552)	ref		46.4% (98)	87.8% (86)	ref	
Often	46.6% (516)	95.7% (494)	1.94 [1.05–3.60]	**0.035∗**	53.6% (113)	89.4% (101)	1.67 [0.54–5.15]	0.376

a Significance as ∗ at p-value <0.05, ∗∗ at p-value <0.01, and ∗∗∗ at p-value <0.001.

b Participants were considered as having illness when reported to have diarrhoea, cough, or fever in the last 2 weeks.

c Analysis only for individuals that owned animals including livestock and domestic animals (n = 666 in O5 and n = 124 in U5).

d Better condition if households used 1) only rain water for drinking, 2) always boiled water before consuming, and 3) had a flushed toilet. Middle if one was missing, and worse if two were missing.

Regarding participants O5, determinants that had a protective effect were the use of rainwater as the only source of drinking water (aOR 0.44, 95% CI: 0.25–0.79), frequent tofu consumption (i.e., more than once per week) (aOR 0.32, 95% CI: 0.14–0.74), frequent fermented food consumption (i.e., more than once per week) (aOR 0.55, 95% CI: 0.31–0.96) and owning pigs within the household (aOR 0.45, 95% CI: 0.25–0.79). Participants O5 who consumed chicken more frequently than once per week were more likely to be colonised (aOR of 3.45, 95% CI: 1.45–8.22) compared to participants who consumed chicken less. The frequent consumption of meat other than pork, chicken, or beef was also associated with an increased odds of C3GRE colonization (aOR 2.89, 95% CI: 1.15–7.29), as was a worse WASH condition (aOR 2.38, 95% CI: 1.32–4.29). Conversely, individuals who reported washing their hands more often were more likely to carry C3GRE (aOR 1.94, 95% CI: 1.05–3.60). An overview of all analysed factors in O5 can be found in Supplementary File 6.

Children U5 living in a household that owned animals had an aOR of 0.27 (95% CI: 0.09–0.84) for C3GRE colonization compared to children who were not. The protective effect was more pronounced for children living in a household with at least two livestock species (aOR 0.12, 95% CI: 0.04–0.39), whereas the odds for children in households with one livestock species or no animals were not significantly different.

Additional information on birth history and immunisation was captured from 203 children U5 (Table 5). All children (100%; 27/27) who were not breastfed within the first hour after delivery carried C3GRE, whereas the prevalence of C3GRE was 88.1% in children who were breastfed immediately after birth. No difference was observed between children who were fully vaccinated and children who were not (aOR 0.57, 95% CI: 0.22–1.48). Results from all analysed factors in U5 are available in Supplementary File 7.

**Table 5:** Univariable and adjusted analyses of C3GRE colonisation and birth and immunisation factors in children under 5 years-of-age (unweighted).

Variable	Total n = 203	C3GRE positive	Crude OR	p-value^a^	OR adjusted for age and SES	p-value^a^	OR adjusted for age, SES and AMU	p-value^a^
**Age**								
<1 year	6.4% (13)	76.9% (10)	ref					
1 year	24.1% (49)	91.8% (45)	3.37 [0.64–17.74]	0.153				
2 years	23.6% (48)	87.5% (42)	2.10 [0.50–8.77]	0.310				
3 years	24.6% (50)	92.0% (46)	3.45 [0.66–17.98]	0.143				
4 years	21.2% (43)	90.7% (39)	2.92 [0.57–15.04]	0.201				
**Socio-economic status (SES)**								
Low income	32.5% (66)	93.9% (62)	Ref					
Middle income	31.0% (63)	87.3% (55)	0.44 [0.13–1.56]	0.208				
High income	36.5% (74)	87.8% (65)	0.47 [0.13–1.65]	0.238				
**Antibiotic use 4 weeks**								
No	68.5% (139)	89.9% (125)	ref		ref			
Yes	31.5% (64)	89.1% (57)	0.91 [0.36–2.28]	0.844	0.94 [0.33–2.73]	0.915		
**Sex**								
Female	45.3% (92)	89.1% (82)	ref		ref		ref	
Male	54.7% (111)	90.1% (100)	1.11 (0.47–2.62)	0.815	1.08 (0.45–2.59)	0.855	1.10 (0.46–2.64)	0.832
**Education**								
In school	36.5% (74)	91.9% (68)	ref		ref		ref	
Attended school before	33.0% (67)	86.6% (58)	0.57 [0.19–1.69]	0.312	0.63 [0.20–1.96]	0.421	0.56 [0.17–1.78]	0.324
Never attended school	30.5% (62)	90.3% (56)	0.82 [0.23–2.97]	0.767	1.03 [0.23–4.59]	0.971	0.98 [0.21–4.54]	0.979
**Delivery**								
Natural delivery	70.4% (143)	88.1% (126)	ref		ref		ref	
Caesarean section	29.6% (60)	93.3% (56)	1.89 [0.60–5.95]	0.277	1.93 [0.63–5.90]	0.248	1.93 [0.63–5.89]	0.248
**Hospitalization**								
Less than 1 day	52.7% (107)	86.9% (93)	ref		ref			
One day or more	47.3% (96)	92.7% (89)	1.91 [0.72–5.06]	0.192	2.03 [0.70–5.84]	0.192	2.05 [0.71–5.90]	0.184
**Early breastfed after birth**								
Early breastfed (<1 h)	86.7% (176)	88.1% (155)	ref		ref		ref	
Later (>1 h)	13.3% (27)	100% (27)	Inf	**<0.001∗∗∗**	Inf	**<0.001∗∗∗**	Inf	**<0.001∗∗∗**
**Exclusive breastfed 4 months**								
No	67.5% (137)	92.0% (126)	ref		ref		ref	
Yes	32.5% (66)	84.8% (56)	0.49 [0.19–1.27]	0.143	0.49 [0.20–1.24]	0,136	0.40 [0.12–1.32]	0,134
**Liquids first 3 days**								
Only mother milk	45.3% (92)	87.0% (80)	ref		ref		ref	
Other milk/infant formula	54.7% (111)	91.9% (102)	1.70 [0.71–4.09]	0.238	1.79 [0.76–4.20]	0.184	1.99 [0.71–5.55]	0.190
**Fully vaccinated**								
Yes	59.1% (120)	92.7% (153)	ref		ref		ref	
No	40.9% (83)	76.5% (26)	0.60 [0.23–1.54]	0.287	0.57 [0.22–1.48]	0.246	0.57 [0.22–1.47]	0.244

a Significance as ∗ at p.

## Discussion

With this study we aimed to quantify the prevalence of C3GRE and CRE in a rural community in Ha Nam in northern Vietnam, and to explore environmental and behavioural determinants. The weighted prevalence of C3GRE was 94.0% in this community, and the weighted prevalence of CRE was 1.9%. These results are consistent with previous data from Southeast Asia, on community carriage of ESBL-producing *E. coli* – which represent the major quantity of C3GRE^3^–in Siem Reap, Cambodia (95.0% in 2019) and rural Bavi, Vietnam in 2013–2014 (87.9% in 2014),^25^^,^^26^ but higher than in Thailand (73.9% among a rural community in 2013 and 66.5% among urban communities in 2014).^15^^,^^27^ A more recent study from southern Vietnam found a CRE prevalence of 0.6% in human faecal samples.^28^ This is aligned with our findings and suggests that the carbapenem resistance has not yet widely spread into the community in Vietnam. In our study, high colonisation in one village may be related to a food or water contamination event, though our study was not designed to identify the source of contamination.

The C3GRE faecal colonization in children under the age of 5 appeared to be lower (88.6%) than in older participants (94.5%), but the statistical power was limited since there were only 203 children under 5. We found the lowest colonization proportion (76.9%) in children younger than 1 year-of-age. Although children were less likely to carry C3GRE, the detected levels were still considerably higher than previously shown for ESBL-producing Enterobacterales among pre-school children in Vientiane, Laos (23.0%) and 4-month infants in Nagasaki, Japan (19.3%).^11^^,^^29^ More in line with the current study results, a recent household study in Cambodia described a high prevalence of ESBL-producing *E. coli* among the community of 95.0%, but a relatively lower prevalence among children under the age of 14 of 92.9%.^26^ The prevalence among children was far above previously detected among healthy children in high-income, Western countries, such as the United States (4.4%), the Netherlands (4.5%), and Sweden (2.9%).^30^, ^31^, ^32^

Although participants U5 reported to consume more antibiotics in the previous month (30.3%) than participants O5 (9.4%), the C3GRE colonization was lower in children. No associations were found between antibiotic consumption or recent illness and C3GRE or CRE carriage. Studies from high-income countries generally show that antibiotic consumption is one of the main risk factors for AMR colonization.^33^ Antibiotic use in Vietnam is persistently high, likely driving high overall levels of C3GRE colonisation. However, the lack of association with individual antibiotic consumption in our study may arise from frequent antibiotic use leading to lower microbial diversity, resulting in microbiota that are more resilient to further perturbations by individual antibiotic use.^34^ Continual transmission of AMR bacteria from animals or the environment may also partly explain the persistence of high levels of resistance in human commensal bacteria regardless of individual antibiotic use.

The household's socio-economic status was one of the main determinants for C3GRE carriage. The proportion of carriers was higher among low-income household members compared to members from middle- and high-income households. These results further support the hypothesis that individuals from low-income environments are at greater risk of getting exposed to antimicrobial-resistant bacteria due to poor hygiene and environmental contamination.^35^^,^^36^ A better WASH condition (i.e., using only rainwater for drinking water, boiling water before consumption, and utilizing a flush toilet) was one of the environmental factors that seemed to have a protective effect against C3GRE colonization in our study. Rainwater, piped supplies, and protected wells and springs are all considered improved drinking water sources by WHO, but contamination of piped drinking water with Enterobacterales has been described in Vietnam.^37^ A household study from Guatemala has previously shown that higher household hygiene had a more significant effect on the prevalence of resistant Enterobacterales than individual antibiotic use.^38^ This is in line with our finding that WASH conditions were associated with C3GRE colonization, and the addition of the household's WASH condition into models had a stronger mediating effect on the multivariable associations than recent illness or antibiotic use. Nevertheless, this association was not consistent across our analyses, and frequent hand-washing was surprisingly associated with higher C3GRE carriage when stratifying by age groups. Thus, we cannot exclude the possibility that the association between WASH conditions and C3GRE carriage reflects other unmeasured confounding factors. Water samples were not collected during this study, but the inclusion of environmental samples in future studies might provide new insights.

Our study found several associations between dietary habits and carriage of C3GRE. Frequent consumption of beef, chicken, and other meat products (excluding pork) was significantly related to higher odds for C3GRE colonization. A study in Thailand previously found that the consumption of undercooked meat was one of the main determinants for colonization with ESBL-producing Enterobacterales.^15^ It may not necessarily be the consumption of meat, but the food hygiene and preparation that explains this association. A study assessing the exposure to C3G-resistant *E. coli* through broiler meat consumption showed that cross-contamination in kitchens is the main route for exposure to resistant bacteria.^39^ Another explanation for the association with meat consumption is the fact that meat products in Vietnam and other LMICs harbour antibiotic residues, thus contributing to the selection of resistant bacteria in the gut microbiota.^40^ Our study also found a similar association between meat consumption and colonization with penicillin non-susceptible *Staphylococcus pneumoniae* in the respiratory tract of this community (also submitted to this journal (Tacoli et al.); *under review*), suggesting that antibiotic residues could be a driving factor rather than bacterial contamination. Whereas meat consumption increased the odds for C3GRE carriage, consuming tofu and fermented food products was observed to have a protective effect on C3GRE colonization. A recent study from the US supported the role of diet, showing that individuals who consumed less animal proteins carried fewer antibiotic resistance genes.^41^ None of the dietary factors were associated with C3GRE colonization in the stratified analysis of children under 5 years-of-age, but the reported household dietary habits may not have reflected the diet of these children.

Our study also investigated the potential role of contact with animals in carriage of resistant bacteria. Participants who owned pigs were less likely to be colonized with C3GRE, and the apparent protective effect of animal husbandry was particularly pronounced in children under 5 years-of-age. A 2012–2013 cohort study in Vietnam similarly showed that chicken farmers were less likely to be colonized with ESBL producing bacteria than other individuals in the rural community.^42^ These findings are contrary to a previous resistome study in the Netherlands, that found that pig farmers and pig slaughterhouse workers harboured higher levels of antimicrobial-resistance genes than poultry farmers and unexposed individuals.^43^ Our findings may be explained by the fact that people who raise animals commercially keep some animals separately for household consumption, and raise these organically. It is unknown to what extent animals in this study were carrying C3GRE, but examining this might result in a better understanding of the relationship between animal husbandry and human AMR colonization.

We also investigated risk factors of C3GRE carriage linked to childhood. All children under 5 that had not been immediately breastfed (i.e., more than 1 h) after birth were harbouring C3GRE. In contrast, carriage was significantly lower (88.1%) in children that were breastfed right after birth. We did not find associations with exclusively breastfeeding, school attendance, in contrast to what has been seen in other countries.^32^^,^^44^ Hospitalization is often considered one of the main risk factors for faecal carriage of resistant Enterobacterales. A previous study from Lebanon suggested that hospitalization might also be a risk factor for children under 5 years-of-age.^44^ Although we observed a similar trend as the previous literature, the sample size limited the statistical power to make any conclusion about the role of this risk factor.

The weighted CRE prevalence was determined to be 1.9%, which is in line with previous studies from Vietnam and Cambodia that found community prevalence levels of respectively 4.0% and 2.1%.^26^^,^^45^ While the C3GRE levels are generally higher in Vietnam and Southeast Asia than in other parts of the world, this did not seem to be the case for CRE. Community studies describe higher prevalence levels in Brazil (6.8%) and Argentina (4.9%).^46^^,^^47^ Healthcare exposure is often considered one of the main risk factors for CRE colonization,^14^^,^^46^ but our results do not confirm this. Findings from a molecular epidemiology study on clinical *E. coli* isolates between 2014 and 2019 from a hospital in Hanoi indicated that carbapenem-resistant isolates were very heterogenous, which also suggests that patients have acquired these isolates from different sources.^48^ Furthermore, the proportion of participants with recent hospital exposure was low and suggests that hospital exposure in general has a limited impact as a population-level risk factor. The geographical and temporal clustering of CRE carriers in our sample suggest that CRE transmission is not occurring at a constant rate, but rather results from focal events. Nevertheless, given the small number of CRE carriers in the dataset, the factors associated with CRE colonization in the present study need to be interpreted with caution, and no causal relationship should be inferred from our results.

The cross-sectional design of this study limits our ability to make causal inferences about associations between risk factors and C3GRE and CRE colonization. Some of the sub-group analyses were underpowered and results should be interpreted with caution. The inclusion of environmental samples (e.g., water, food, animals) in future studies might result in a better explanation of these associations. The current findings can be incorporated in the design of community-based interventions to further examine to what extent environmental, behavioural, and contextual determinants are related to colonization with resistant Enterobacterales. We did not find any significant associations between likelihood of colonization with CRE or C3GRE and antibiotic use. Measurement of risk factors was based on recall and might have been prone to misclassification, which may have biased the result towards the null. Our direct plating method may have lacked sensitivity, and it is possible that individuals classified as negative could have been colonised with very low concentrations of CRE or C3GRE. However, to identify risk factors for colonisation with these resistant strains, we aimed to focus on people who were highly colonised and therefore more likely to transmit resistant bacteria. We did not perform tests to confirm the identification of isolates or identify the resistance mechanism, as this study did not focus on the epidemiology of a single species or resistance mechanism. The main aim of this study was to produce a low-cost, simple, and reproducible measure of the prevalence of resistance from a large population sample in the community, and use this methodology to identify risk factors for colonisation. To the best of our knowledge, MacConKey agar No. 3 gives improved differentiation between lactose and non-lactose fermenting organisms whilst Gram-positive cocci are completely inhibited. All lactose fermenting colonies growing on MacConkey agar belong to Enterobacterales,^49^ and no Enterobacterales has a constitutive resistance to carbapenems or 3rd generation cephalosporins.^50^ The large number of samples prohibited using an enumeration approach to measure the concentration of resistant strains in colonised individuals as well as testing for a wider range of phenotypic resistance to other antibiotics. Our exposure data lacked details of specific antibiotics used, therefore we were unable to say whether exposure to specific antibiotics was associated with specific resistance.

This study provides estimates of colonization levels with third-generation cephalosporin- and carbapenem-resistant Enterobacterales in healthy individuals in a rural community in north Vietnam. These results may not be generalizable to urban areas or other regions of Vietnam. The community carriage of CRE was limited in the community, but C3GRE prevalence was very high, and residents of this rural community are at increased risk for developing infections caused by resistant Enterobacterales. Recent antibiotic use and recent illness was not associated with C3GRE carriage, while the household's socioeconomic status, food, hygiene and sanitation condition, and animal ownership were. Our data suggest that antimicrobial resistance in LMIC is driven by environmental transmission. The findings from our study are in line with a longitudinal cohort study among households in Malawi which also found no association between antibiotic use and carriage of ESBL-producing Enterobacterales, but did find environmental drivers for colonization.^51^

While further research is needed to understand the mechanisms for these associations, including molecular work to identify resistance genes, it can be said that a One Health approach should be integrated in the design of interventions that aim to tackle AMR in similar settings. It will not be enough to focus only on antibiotic consumption, but environmental components must be included for interventions tackling AMR in the community.

## Contributors

SL, HRvD, BNTV, HATN, HHT conceptualised and designed the study, acquired funding, and had final responsibility for the decision to submit for publication. TCTN coordinated study implementation and planned and executed research activities with support from TDT and DTN. BNTV, HHT, HATN, QDP, KHTT, THTN, TTN and PDT conducted lab analyses. CT, TST, and DTVV led data curation and managed and prepared survey datasets and verified the data. MvW conceptualized and conducted analyses and wrote the first draft of the manuscript. TK supervised analysis and interpretation. MvW, TK, CT, TST, DTVV and SL had access to the raw data. DAD supervised implementation and was responsible for liaison with local partners. All authors reviewed and commented on the manuscript and read and approved the final version.

## Data sharing statement

Datasets generated and analysed during the current study are not publicly available as this was not included in the consent process, but anonymised datasets can be made available from the corresponding author on reasonable request.

## Editor note

The Lancet Group takes a neutral position with respect to territorial claims in published maps and institutional affiliations.

## Declaration of interests

H. Rogier van Doorn is a board member for Wellcome SEDRIC (Surveillance and Epidemiology of Drug Resistant Infections Consortium). Thomas Kesteman received support from Medecins Sans Frontieres France (MSF-OCP) to attend a conference, is member of the scientific committee for the Mini-Lab project on AMR, MSF-OCP 2016–2022. Max van Wijk received consulting fees from the Quadripartite Joint Secretariat on AMR to draft the first biannual report on AMR in 2022, and from IS Global to contribute to a report on AMR in 2022/23, commissioned by DG SANTE/European Commission. The remaining authors declare that they have no competing interests.

## Appendix A Supplementary data

Supplementary Figures and Tables

Abstract_VN_clean
