# Supplementary material for: Prevalence and determinants of faecal carriage of carbapenem- and third-generation cephalosporin-resistant Enterobacterales: a cross-sectional household survey in northern Vietnam
Source: Lancet Reg Health West Pac. 2025 Jan 13;54:101281. doi: 10.1016/j.lanwpc.2024.101281 (PMC11780954; doi:10.1016/j.lanwpc.2024.101281)
Supplement: Supplementary Figures and Tables [file mmc1.docx]

**Supplementary figures and tables**

**Supplementary file 1:** Household survey

**Supplementary file 2**: List of curated variables

**Supplementary file 4**: Complete list of the CRE household analyses (weighted)

**Supplementary file 3**: Overview of CRE and C3GRE carriage rates stratified by village

**Supplementary file 5**: Complete list of the C3GRE individual analyses (weighted)

**Supplementary file 6**: Complete list of stratified C3GRE analyses (unweighted)

**Supplementary file 7**: Complete list of C3GRE analyses of birth & immunisation factors (unweighted)

**Supplementary file 1:** Household survey

**1. Interview details**

Household number | 2 | 3 |-|__|__|-|__|__|__|__| (composite of study number, village and household)

Today’s date |__|__|/|__|__|/| 1 | 8 |

Name of interviewer ……………………………………………………………

Name of interviewee ……………………………………………………………

Role of interviewee in household 1 = Head

2 = Spouse/partner

3 = Son/daughter

4 = Son-in-law/daughter-in-law

5 = Grandchild

6 = Parent

7 = Parent-in-law

8 = Brother/sister

9 = Brother-in-law/sister-in-law

10 = Other

MY NAME IS:

WE ARE FROM

WE ARE CONDUCTING A SURVEY ABOUT THE HEALTH AND HEALTH-SEEKING PATTERNS OF FAMLIES, AND FACTORS THAT MAY BE ASSOCIATED WITH THIS. I WOULD LIKE TO TALK TO YOU ABOUT THESE SUBJECTS. THE INTERVIEW WILL TAKE ABOUT **60** MINUTES. ALL THE INFORMATION WE OBTAIN WILL REMAIN STRICTLY CONFIDENTIAL AND ANONYMOUS.

MAY I START NOW?

|__| *Yes, permission is given* 🡪 *Record the time and then begin the interview.*

|__| *No, permission is not given* 🡪 *Circle 4 in below. Discuss this result with your supervisor.*

**Result of household interview:**

1 = Completed

2 = No household member or no competent respondent at home at time of visit

3 = Entire household absent for extended period of time

4 = Refused

5 = Dwelling vacant / Address not a dwelling

6 = Dwelling destroyed

7 = Dwelling not found

8 = Other (*specify*_________________________________________________________________ )

Time of starting interview |__|__|:|__|__|

**2. Household members**

Can you tell me the names and details of all of the people who normally live in this household? That is, they have lived here for 3 months or more and intend to stay here permanently.

| # | **2.1** Name | **2.2** Sex | **2.3** Date of birth | **2.4** Position in household | **2.5** Has (NAME) ever attended school or pre-school? If so, What is the highest level of school (NAME) has attended? | **2.6** Is (NAME) still attending pre-school, school, or university? If yes, which one? | **2.7** How many days per week does (NAME) attend? | **2.8** How many hours per day does (NAME) attend? | **2.9** What is (NAME)’s current occupation? | **2.10** Does (NAME) have a medical insurance card? |
| --- | --- | --- | --- | --- | --- | --- | --- | --- | --- | --- |
|  |  | 1 = M  2 = F |  | 1 = Head  2 =Spouse/partner  3 = Son/daughter  4 = Son-in-law/daughter-in-law  5 = Grandchild  6 = Parent  7 = Parent-in-law  8 = Brother/sister  9 = Brother-in-law/sister-in-law  10 = Other | 0 = None 🡪 Q2.9  1 = Organised preschool learning programme/early childhood education/ kindergarten/community childcare group  2 = Primary  3 = Lower secondary  4 = Upper secondary  5 = Professional school  6 = College/University & above  9 = Don’t Know 🡪 Q2.9 | 0 = No 🡪 Q2.9  1 = Yes (specify) |  |  | 0 = Not currently working  1 = Farmer  2 = Labourer  3 = Factory worker  4 = Office worker  5 = Shop/retail/  Hospitality  6 = Health worker  7 = Other (specify) | 0 = No  1 = Yes  9 = Don’t know |
| 1 |  | \|__\| | \|__\|__\|/\|__\|__\|/\|__\|__\| | \|__\| | \|__\| | \|__\| | \|__\| | \|__\|__\| | \|__\| | \|__\| |
| 2 |  | \|__\| | \|__\|__\|/\|__\|__\|/\|__\|__\| | \|__\| | \|__\| | \|__\| | \|__\| | \|__\|__\| | \|__\| | \|__\| |
| 3 |  | \|__\| | \|__\|__\|/\|__\|__\|/\|__\|__\| | \|__\| | \|__\| | \|__\| | \|__\| | \|__\|__\| | \|__\| | \|__\| |
| 4 |  | \|__\| | \|__\|__\|/\|__\|__\|/\|__\|__\| | \|__\| | \|__\| | \|__\| | \|__\| | \|__\|__\| | \|__\| | \|__\| |
| 5 |  | \|__\| | \|__\|__\|/\|__\|__\|/\|__\|__\| | \|__\| | \|__\| | \|__\| | \|__\| | \|__\|__\| | \|__\| | \|__\| |
| 6 |  | \|__\| | \|__\|__\|/\|__\|__\|/\|__\|__\| | \|__\| | \|__\| | \|__\| | \|__\| | \|__\|__\| | \|__\| | \|__\| |
| 7 |  | \|__\| | \|__\|__\|/\|__\|__\|/\|__\|__\| | \|__\| | \|__\| | \|__\| | \|__\| | \|__\|__\| | \|__\| | \|__\| |
| 8 |  | \|__\| | \|__\|__\|/\|__\|__\|/\|__\|__\| | \|__\| | \|__\| | \|__\| | \|__\| | \|__\|__\| | \|__\| | \|__\| |
| 9 |  | \|__\| | \|__\|__\|/\|__\|__\|/\|__\|__\| | \|__\| | \|__\| | \|__\| | \|__\| | \|__\|__\| | \|__\| | \|__\| |
| 10 |  | \|__\| | \|__\|__\|/\|__\|__\|/\|__\|__\| | \|__\| | \|__\| | \|__\| | \|__\| | \|__\|__\| | \|__\| | \|__\| |

**3. Household socioeconomic and demographic information, access to health information**

|  | **OBSERVATION** | | | |
| --- | --- | --- | --- | --- |
| **#** | **Question** | | **Responses** | |
|  | **Household socioeconomic and demographic observations** | |  | |
|  | How many floors does this house have?  *(observe)*  *Record 9 if not known*  *Record 8 if this is an apartment building and this house occupies only 1 or 2 floors* | | \|__\| floors | |
|  | Main material of the dwelling floor  *(observe)* | | 1 = Earth / Sand  2 = Wood planks  3 = Palm / Bamboo  4 = Parquet or polished wood  5 = Vinyl or asphalt strips  6 = Ceramic tiles  7 = Cement  8 = Carpet  9 = Enamelled tiles/ marble  10 = Other (*specify* ____________________________) | |
|  | Main material of the roof  *(observe)* | | 1 = No Roof  2 = Thatch / Palm leaf  3 = Palm / Bamboo  4 = Wood planks  5 = Cardboard  6 = Metal / Tin  7 = Wood  8 = Calamine / Cement fibre  9 = Ceramic tiles  10 = Cement  11 = Stone slates  12 = Asphalt sheets  13 = Other (*specify* ____________________________) | |
|  | Main material of the exterior walls  *(observe)* | | 1 = No walls  2 = Cane / Palm / Trunks  3 = Dirt  4 = Reed  5 = Bamboo with mud  6 = Stone with mud  7 = Uncovered adobe  8 = Plywood  9 = Cardboard  10 = Reused wood  11 = Cement  12 = Stone with lime / cement  13 = Bricks  14 = Cement blocks  15 = Covered adobe  16 = Wood planks / shingles  17 = Other (*specify* ____________________________) | |
|  | We would like to learn about the places that households use to wash their hands. Can you please show me where members of your household most often wash their hands? | | 1 = Observed  *Not observed*  2 = Not in dwelling / plot / yard  3 = No permission to see  4 = Other reason (*specify* _______________________) | |
|  | *Observe presence of water at the place for handwashing.*  *Verify by checking the tap/pump, or basin, bucket, water container or similar objects for presence of water.* | | 0 = Water is not available  1 = Water is available | |
|  | *Is soap, detergent present at the place for handwashing?*  *Record your observation.*  *Circle all that apply.* | | 0 = No, not present 🡪 Q3.8  1 = Yes, present 🡪 *circle all that apply then got to 🡪 Q3.11*  a) Bar soap  b) Detergent (Powder / Liquid / Paste)  c) Liquid soap | |
|  | Do you have any soap or detergent in your house for washing hands? | | 0 = No 🡪Q3.11  1 = Yes | |
|  | Can you please show it to me? | | 0 = No, not shown 🡪Q3.11  1 = Yes, shown | |
|  | *Record your observation.*  *Circle all that apply.* | | 1 = Bar soap  2 = Detergent (Powder / Liquid / Paste)  3 = Liquid soap | |
|  | How often do members of your household wash their hands with soap before or after each of these activities?  *Tick appropriate column for each activity*  *If there is no baby in the household, record NA for f) and h)*   \|  \| Never \| Sometimes \| Most of the time \| Always \|  \| \| --- \| --- \| --- \| --- \| --- \| --- \| \| a) After toilet \|  \|  \|  \|  \| \| b) Before cooking \|  \|  \|  \|  \| \| c) After cooking \|  \|  \|  \|  \| \| d) Before eating \|  \|  \|  \|  \| \| e) After eating \|  \|  \|  \|  \| \| f) After cleaning baby’s backside \|  \|  \|  \|  \| \| h) Before feeding baby \|  \|  \|  \|  \| \| i) After handling animals \|  \|  \|  \|  \| \| j) After sneezing or coughing into hands \|  \|  \|  \|  \| | | |  |
|  | **Household socioeconomic and demographic questions** |  | |  |
|  | How many rooms in this household are used for sleeping? | \|__\|__\| rooms | |  |
|  | How many M^2^ is the area of this house?  *Record 999 if not known* | \|__\|__\|__\| m^2^ | |  |
|  | Does your household have:  *(Tick all that apply)* | \|__\| a) Electricity?  \|__\| b) A radio?  \|__\| c) A television?  \|__\| d) A fixed telephone?  \|__\| e) A mobile telephone  \|__\| f) A refrigerator?  \|__\| g) A bed?  \|__\| h) A table and chair set?  \|__\| i) Sofa?  \|__\| j) A fan?  \|__\| k) A desktop or laptop computer?  \|__\| l) A tablet PC or iPad  \|__\| m) An air conditioner?  \|__\| n) A gas cooker?  \|__\| o) An electric cooker?  \|__\| p) A washing machine?  \|__\| q) Bicycle  \|__\| r) Motorcycle / Scooter  \|__\| s) A tractor?  \|__\| t) A car or truck?  \|__\| u) A ship or boat with a motor? | |  |
|  | Does this household own any livestock, herds, other farm animals, or poultry? | 0 = No 🡪Q3.19  1 = Yes | |  |
|  | How many of the following animals does the household have?  *If none, record ‘00’. If 95 or more, record ‘95’. If unknown, record ‘98’.* | \|__\|__\| a) Cattle, milk cows, bulls or buffalo  \|__\|__\| b) Horses, donkeys, or mules  \|__\|__\| c) Goats  \|__\|__\| d) Chickens or quails  \|__\|__\| e) Pigs  \|__\|__\| f) Ducks, Muscovy ducks, geese or swans  \|__\|__\| h) Farmed fish  \|__\|__\| i) Other (specify_______________________) | |  |
|  | Do you use antibiotics for raising these animals? If yes, for what reason?  *(Circle all mentioned)* | 0 = Don’t use antibiotics 🡪Q3.19  1 = To promote growth  2 = To prevent diseases  3 = To treat diseases  4 = Other (specify_____________________________)  9 = Don’t know | |  |
|  | How often do you use antibiotics for any of your animals? | 0 = Never  1 = A few times per year  2 = Less than once per month  3 = At least once per month  4 = At least once per week  5 = Every day  9 = Don’t know | |  |
|  | What is the main source of drinking water for members of your household? | 1 = Piped into dwelling  2 = Piped into compound, yard or plot  3 = Piped to neighbour  4 = Public tap / standpipe  5 = Tube Well, Borehole  6 = Protected well  7 = Unprotected well  8 = Rainwater collection  9 = Surface water (river, stream, dam, lake, pond, canal, irrigation channel)  10 = Bottled water  11 = Other (*specify* ____________________________) | |  |
|  | Do you do anything to the water to make it safer to drink?  *Probe:*  Anything else?  *Circle all items mentioned* | 0 = No treatment  1 = Boil  2 = Add bleach / chlorine  3 = Strain it through a cloth  4 = Use water filter (ceramic, sand, composite, etc.)  5 = Solar disinfection  6 = Let it stand and settle  7 = Other (*specify* _____________________________)  8 = Don’t know | |  |
|  | What is the main source of water used by your household for other purposes such as cooking and handwashing? | 1 = Piped into dwelling  2 = Piped into compound, yard or plot  3 = Piped to neighbour  4 = Public tap / standpipe  5 = Tube Well, Borehole  6 = Protected well  7 = Unprotected well  8 = Rainwater collection  9 = Surface water (river, stream, dam, lake,  pond, canal, irrigation channel)  10 = Other (*specify* ____________________________) | |  |
|  | What kind of toilet facility do members of your household usually use?  *If “flush” or “pour flush”, probe:*  Where does it flush to?  *If not possible to determine, ask permission to observe the facility.* | 1 = Flush to piped sewer system  2= Flush to septic tank  3 = Flush to pit (latrine)  4 = Flush to somewhere else  5 = Flush to unknown place / Not sure /DK where  6 = Ventilated Improved Pit latrine (VIP)  7 = Pit latrine with slab  8 = Pit latrine without slab / Open pit  9 = Composting toilet  10 = Bucket  11 = Hanging toilet, Hanging latrine  12 = No facility, Bush, Field  13 = Other (*specify* ____________________________) | |  |
|  | Do you share this facility with others who are not members of your household? | 0 = No 🡪Q3.25  1 = Other households only (not public)  2 = Public facility | |  |
|  | How many households in total use this toilet facility, including your own household? | Number of households \|__\|__\|  10 = Ten or more households  98 = Don’t know | |  |
|  | Do you grow vegetables? | 0 = No 🡪 Q3.27  1 = Yes | |  |
|  | What fertilizer do you use for growing vegetables? If human/animal stool, is it composted or uncomposted before use? | 1 = Uncomposted human stool  2 = Composted human stool  3 = Uncomposted animal stool  4 = Composted animal stool  5 = Chemical fertilizer  6 = Other (specify _____________________________) | |  |
|  | What type of fuel does your household mainly use for cooking? | 1 = Electricity 🡪Q3.29  2 = Gas 🡪Q3.29  3 = Biogas 🡪Q3.29  4 = Kerosene 🡪Q3.29  5 = Coal / Lignite  6 = Charcoal  7 = Wood  8 = Straw / Shrubs / Grass  9 = Animal dung  10 = Agricultural crop residue  11 = No food cooked in household  12 = Other (*specify* ____________________________) | |  |
|  | Is the cooking usually done in the house, in a separate building, or outdoors?  *If ‘In the house’, probe*: Is it done in a separate room used as a kitchen? | 1 = In the house, in a separate room used as kitchen  2 = Elsewhere in the house  3 = In a separate building  4 = Outdoors  5 = Other (*specify* _____________________________) | |  |
|  | How often does anyone smoke inside your house?  Would you say daily, weekly, monthly, less than monthly, or never? | 1 = Daily  2 = Weekly  3 = Monthly  4 = Less than monthly  5 = Never | |  |
|  | **Household food consumption** |  | |  |
| 1. 3.**30** | How often do you consume the following food items:  *Tick appropriate column for each food type*   \|  \| Never \| Less than once per week \| Once per week \| More than once per week \| Every day \| \| --- \| --- \| --- \| --- \| --- \| --- \| \| a) Starchy foods like cereals and grains such as rice, noodles, bread, or maize, or roots and tubers such as potato, yam, cassava, sweet potatoes, taro? \|  \|  \|  \|  \|  \| \| b) Pulses/nuts such as beans, peas, peanuts, lentils, soy beans, other nuts? \|  \|  \|  \|  \|  \| \| c) Vegetables? \|  \|  \|  \|  \|  \| \| d) Fruits? \|  \|  \|  \|  \|  \| \| e) Chicken? \|  \|  \|  \|  \|  \| \| f) Pork? \|  \|  \|  \|  \|  \| \| g) Beef? \|  \|  \|  \|  \|  \| \| h) Other meat? \|  \|  \|  \|  \|  \| \| i) Shrimps? \|  \|  \|  \|  \|  \| \| j) Fish or other seafood? \|  \|  \|  \|  \|  \| \| k) Egg? \|  \|  \|  \|  \|  \| \| l) Tofu? \|  \|  \|  \|  \|  \| \| m) Milk and other dairy products such as yogurt or cheese? \|  \|  \|  \|  \|  \| \| n) Homemade fermented foods such as nem chua, dua chua, mam chua, tom chua, sua chua, or bought probiotics such as yakult? \|  \|  \|  \|  \|  \| | | |  |
|  | **Access to mass media and health information** |  | |  |
|  | During the last month, how often did you read a printed newspaper or magazine: almost every day, at least once a week, less than once a week, or not at all? | 1 = Almost every day  2 = At least once a week  3 = Less than once a week  4 = Not at all | |  |
|  | During the last month, how often did you listen to the radio: almost every day, at least once a week, less than once a week, or not at all? | 1 = Almost every day  2 = At least once a week  3 = Less than once a week  4 = Not at all | |  |
|  | During the last month, how often did you watch television: almost every day, at least once a week, less than once a week, or not at all? | 1 = Almost every day  2 = At least once a week  3 = Less than once a week  4 = Not at all | |  |
|  | During the last month, how often did you read or write SMS messages: almost every day, at least once a week, less than once a week, or not at all? | 1 = Almost every day  2 = At least once a week  3 = Less than once a week  4 = Not at all | |  |
|  | In the last 12 months have you used the internet?  *If necessary, probe for use from any location, with any device.* | 0 = No 🡪Q3.39  1 = Yes | |  |
|  | During the last month, how often did you use the internet: almost every day, at least once a week, less than once a week, or not at all? | 1 = Almost every day  2 = At least once a week  3 = Less than once a week  4 = Not at all | |  |
|  | In the last 12 months, have you used any social media sites, such as facebook, zalo, viber, whatsapp, twitter? | 0= No, never used any social media sites🡪Q3.39  1 = Facebook  2 = Zalo  3 = Viber  4 = WhatsApp  5 = Twitter  6 = Other (specify ________________________) | |  |
|  | During the last month, how often did you use social media sites? | 1 = Almost every day  2 = At least once a week  3 = Less than once a week  4 = Not at all | |  |
|  | How often do you get information about how to keep your family healthy from each of these sources?  *Ask for each source and tick the appropriate column*   \|  \| Never \| Sometimes \| Often \| Very often \| \| --- \| --- \| --- \| --- \| --- \| \| Television \|  \|  \|  \|  \| \| Radio \|  \|  \|  \|  \| \| Printed newspaper \|  \|  \|  \|  \| \| Magazine \|  \|  \|  \|  \| \| Social media \|  \|  \|  \|  \| \| Commune radio speaker \|  \|  \|  \|  \| \| Health worker \|  \|  \|  \|  \| \| Pharmacist/drug store worker \|  \|  \|  \|  \| \| Relatives \|  \|  \|  \|  \| \| Friends \|  \|  \|  \|  \| \| Community leader (e.g. village leader) \|  \|  \|  \|  \| \| Women’s union \|  \|  \|  \|  \| \| Books \|  \|  \|  \|  \| \| Internet (specify sites__________________) \|  \|  \|  \|  \| | | |  |
|  | Have you ever heard of a type of medicine called an antibiotic? | 0 = No 🡪 Q4.1  1 = Yes  9 = Don’t know 🡪 Q4.1 | |  |
|  | Which antibiotics have you heard of?  *(Tick those spontaneously mentioned in the first column)*  Now I am going to mention some antibiotics and I want you to let me know if you have heard of them.  *(Tick those recognised after prompting in the second column)*   \|  \| Mentioned spontaneously \| Mentioned after probing \| Don’t know \| \| --- \| --- \| --- \| --- \| \| Penicillin \|  \|  \|  \| \| Doxycycline \|  \|  \|  \| \| Tetracycline \|  \|  \|  \| \| Erythromycin \|  \|  \|  \| \| Ampicillin/ Amoxicillin \|  \|  \|  \| \| Augmentin \|  \|  \|  \| \| Streptomycin \|  \|  \|  \| \| Cotrimoxazole \|  \|  \|  \| \| Cephalexin \|  \|  \|  \| \| Ciprofloxacin \|  \|  \|  \| \| Colistin \|  \|  \|  \|   Other (specify): …………………………………………………………………………………………………………..........................................................  …………………………………………………………………………………………………………………………………………………………………………………………..  …………………………………………………………………………………………………………………………………………………………………………………………..  …………………………………………………………………………………………………………………………………………………………………………………………..  ………………………………………………………………………………………………………………………………………………………………………………………….. | | |  |

**4. Birth history and immunisation** (asked for all children UNDER 5 YEARS)

Now I would like to ask you some questions about all of the children under 5 years in your house.

|  | Name from Table 2 | Last birth  Name_________________ | Next-to-last birth  Name ____________ | Second-from-last birth  Name _____________ |
| --- | --- | --- | --- | --- |
| # | # Number from Table 2 | \|__\| | \|__\| | \|__\| |
| **4.1** | Was (NAME) born by caesarean section? | 0 = No  1 = Yes | 0 = No  1 = Yes | 0 = No  1 = Yes |
| **4.2** | Did (NAME) have to stay in hospital for any reason after he/she was born? If yes, for how many days/weeks?  *If less than 1 day, record ‘00’ days.*  *If less than 7 days, record days.*  *Otherwise, record weeks.* | \|__\|__\| \|__\|__\| Days Weeks | \|__\|__\| \|__\|__\| Days Weeks | \|__\|__\| \|__\|__\| Days Weeks |
| **4.3** | Has (NAME) ever been breastfed? | 0 = No 🡪 Q4.11  1 = Yes  9 = Don’t Know 🡪Q4.11 | 0 = No 🡪 Q4.11  1 = Yes  9 = Don’t Know 🡪Q4.11 | 0 = No 🡪 Q4.11  1 = Yes  9 = Don’t Know 🡪Q4.11 |
| **4.4** | How long after birth did you first put (NAME) to the breast?  *If less than 1 hour, record ‘00’ hours.*  *If less than 24 hours, record hours.*  *Otherwise, record days.* | 00 = Immediately  \|__\|__\| Hours \|__\|__\| Days  99 = Don’t know / Don’t remember | 00 = Immediately  \|__\|__\| Hours \|__\|__\| Days  99 = Don’t know / Don’t remember | 00 = Immediately  \|__\|__\| Hours \|__\|__\| Days  99 = Don’t know / Don’t remember |
| **4.5** | In the first three days after delivery, was (NAME) given anything to drink other than breast milk? | 0 = No 🡪 Q4.7  1 = Yes  9 = Don’t know 🡪 Q4.7 | 0 = No 🡪 Q4.7  1 = Yes  9 = Don’t know 🡪 Q4.7 | 0 = No 🡪 Q4.7  1 = Yes  9 = Don’t know 🡪 Q4.7 |
| **4.6** | What was (NAME) given to drink?  *Probe:*  Anything else?  *(Circle all mentioned)* | 1 = Milk (other than breast milk)  2 = Plain water  3 = Sugar or glucose water  4 = Gripe water  5 = Sugar-salt-water solution  6 = Fruit juice  7 = Infant formula  8 = Tea / Infusions  9 = Honey  10 = Rice soup  11 = Other (*specify*______) | 1 = Milk (other than breast milk)  2 = Plain water  3 = Sugar or glucose water  4 = Gripe water  5 = Sugar-salt-water solution  6 = Fruit juice  7 = Infant formula  8 = Tea / Infusions  9 = Honey  10 = Rice soup  11 = Other (*specify*______) | 1 = Milk (other than breast milk)  2 = Plain water  3 = Sugar or glucose water  4 = Gripe water  5 = Sugar-salt-water solution  6 = Fruit juice  7 = Infant formula  8 = Tea / Infusions  9 = Honey  10 = Rice soup  11 = Other (*specify*______) |
| **4.7** | Is (NAME) still being breastfed? | 1 = Yes 🡪 Q4.9  2 = No  3 = DK | 1 = Yes 🡪 Q4.9  2 = No  3 = DK | 1 = Yes 🡪 Q4.9  2 = No  3 = DK |
| **4.8** | For how long was (NAME) breastfed?  *If less than 1 week, record ‘00’ weeks.*  *If less than 5 weeks, record weeks.*  *Otherwise, record months.* | \|__\| Weeks \|__\|__\| Months  99 If still breastfed | \|__\| Weeks \|__\|__\| Months  99 If still breastfed | \|__\| Weeks \|__\|__\| Months  99 If still breastfed |
| **4.9** | At what age did (NAME) start drinking liquids other than breast milk?  *If less than 1 week, record ‘00’ weeks.*  *If less than 5 weeks, record weeks.*  *Otherwise, record months.*  *Probe: milk, water, sugar water, gripe water, fruit juice, infant formula, tea/infusions, honey, rice soup?* | \|__\| Weeks \|__\|__\| Months  99 If still exclusively breastfed | \|__\| Weeks \|__\|__\| Months  99 If still exclusively breastfed | \|__\| Weeks \|__\|__\| Months  99 If still exclusively breastfed |
| **4.10** | At what age did (NAME) start eating foods other than breast milk?  *If less than 1 week, record ‘00’ weeks.*  *If less than 5 weeks, record weeks.*  *Otherwise, record months.* | \|__\| Weeks \|__\|__\| Months  99 If still exclusively breastfed | \|__\| Weeks \|__\|__\| Months  99 If still exclusively breastfed | \|__\| Weeks \|__\|__\| Months  99 If still exclusively breastfed |
| **4.11** | Do you have a card/child health book where (NAME)’s vaccinations are written down?  IF YES: May I see it please? | 0 = No 🡪 Q4.13  1 = Yes  2 = Yes, but no permission to see it/not available  🡪Q 4.13 | 0 = No 🡪 Q4.13  1 = Yes  2 = Yes, but no permission to see it/not available  🡪Q 4.13 | 0 = No 🡪 Q4.13  1 = Yes  2 = Yes, but no permission to see it/not available  🡪Q 4.13 |
| **4.12** | BCG  OPV1  OPV2  OPV3  Hepatitis B (birth dose)  DPT, Hepatitis, Hib, 1^st^ dose  DPT, Hepatitis, Hib, 2^nd^ dose  DPT, Hepatitis, Hib, 3^rd^ dose  DPT (booster dose)  Measles (or MMR or MR) 1  Measles (or MMR or MR) 2  Vitamin A (1^st^ dose)  Vitamin A (2^nd^ dose)  Al/Mebendazole (most recent)  *These vaccines are not in the EPI schedule for Vietnam, but may be given*  Pneumococcal vaccine 1  Pneumococcal vaccine 2  Pneumococcal vaccine 3  Rotavirus vaccine 1  Rotavirus vaccine 2 🡪 Q4.21 | \|__\|__\|/\|__\|__\|/\|__\|__\|  \|__\|__\|/\|__\|__\|/\|__\|__\|  \|__\|__\|/\|__\|__\|/\|__\|__\|  \|__\|__\|/\|__\|__\|/\|__\|__\|  \|__\|__\|/\|__\|__\|/\|__\|__\|  \|__\|__\|/\|__\|__\|/\|__\|__\|  \|__\|__\|/\|__\|__\|/\|__\|__\|  \|__\|__\|/\|__\|__\|/\|__\|__\|  \|__\|__\|/\|__\|__\|/\|__\|__\|  \|__\|__\|/\|__\|__\|/\|__\|__\|  \|__\|__\|/\|__\|__\|/\|__\|__\|  \|__\|__\|/\|__\|__\|/\|__\|__\|  \|__\|__\|/\|__\|__\|/\|__\|__\|  \|__\|__\|/\|__\|__\|/\|__\|__\|  \|__\|__\|/\|__\|__\|/\|__\|__\|  \|__\|__\|/\|__\|__\|/\|__\|__\|  \|__\|__\|/\|__\|__\|/\|__\|__\|  \|__\|__\|/\|__\|__\|/\|__\|__\|  \|__\|__\|/\|__\|__\|/\|__\|__\| | \|__\|__\|/\|__\|__\|/\|__\|__\|  \|__\|__\|/\|__\|__\|/\|__\|__\|  \|__\|__\|/\|__\|__\|/\|__\|__\|  \|__\|__\|/\|__\|__\|/\|__\|__\|  \|__\|__\|/\|__\|__\|/\|__\|__\|  \|__\|__\|/\|__\|__\|/\|__\|__\|  \|__\|__\|/\|__\|__\|/\|__\|__\|  \|__\|__\|/\|__\|__\|/\|__\|__\|  \|__\|__\|/\|__\|__\|/\|__\|__\|  \|__\|__\|/\|__\|__\|/\|__\|__\|  \|__\|__\|/\|__\|__\|/\|__\|__\|  \|__\|__\|/\|__\|__\|/\|__\|__\|  \|__\|__\|/\|__\|__\|/\|__\|__\|  \|__\|__\|/\|__\|__\|/\|__\|__\|  \|__\|__\|/\|__\|__\|/\|__\|__\|  \|__\|__\|/\|__\|__\|/\|__\|__\|  \|__\|__\|/\|__\|__\|/\|__\|__\|  \|__\|__\|/\|__\|__\|/\|__\|__\|  \|__\|__\|/\|__\|__\|/\|__\|__\| | \|__\|__\|/\|__\|__\|/\|__\|__\|  \|__\|__\|/\|__\|__\|/\|__\|__\|  \|__\|__\|/\|__\|__\|/\|__\|__\|  \|__\|__\|/\|__\|__\|/\|__\|__\|  \|__\|__\|/\|__\|__\|/\|__\|__\|  \|__\|__\|/\|__\|__\|/\|__\|__\|  \|__\|__\|/\|__\|__\|/\|__\|__\|  \|__\|__\|/\|__\|__\|/\|__\|__\|  \|__\|__\|/\|__\|__\|/\|__\|__\|  \|__\|__\|/\|__\|__\|/\|__\|__\|  \|__\|__\|/\|__\|__\|/\|__\|__\|  \|__\|__\|/\|__\|__\|/\|__\|__\|  \|__\|__\|/\|__\|__\|/\|__\|__\|  \|__\|__\|/\|__\|__\|/\|__\|__\|  \|__\|__\|/\|__\|__\|/\|__\|__\|  \|__\|__\|/\|__\|__\|/\|__\|__\|  \|__\|__\|/\|__\|__\|/\|__\|__\|  \|__\|__\|/\|__\|__\|/\|__\|__\|  \|__\|__\|/\|__\|__\|/\|__\|__\| |
| **4.13** | Did (NAME) ever have any vaccinations to prevent him/her from getting diseases, including vaccinations received in a campaign or immunisation day or child health day? | 0 = No 🡪 Q4.21  1 = Yes  9 = Don’t Know 🡪 Q4.21 | 0 = No 🡪 Q4.21  1 = Yes  9 = Don’t Know 🡪 Q4.21 | 0 = No 🡪 Q4.21  1 = Yes  9 = Don’t Know 🡪 Q4.21 |
| **4.14** | Has (NAME) ever received a BCG vaccination – An injection in the arm or shoulder that usually causes a scar | 0 = No  1 = Yes  9 = Don’t know | 0 = No  1 = Yes  9 = Don’t know | 0 = No  1 = Yes  9 = Don’t know |
| **4.15** | Has (NAME) ever received a polio vaccination – Vaccination drops in the mouth to protect him/her from polio? If yes, how many times was it received? | 0 = No  1 = Yes, \|__\| times  9 = Don’t know | 0 = No  1 = Yes, \|__\| times  9 = Don’t know | 0 = No  1 = Yes, \|__\| times  9 = Don’t know |
| **4.16** | Has (NAME) ever received pentavalent vaccine – An injection in the thigh – to prevent him/her from getting tetanus, whooping cough, diphtheria, hepatitis B and Hib B? If yes, how many times was it received? | 0 = No  1 = Yes, \|__\| times  9 = Don’t know | 0 = No  1 = Yes, \|__\| times  9 = Don’t know | 0 = No  1 = Yes, \|__\| times  9 = Don’t know |
| **4.17** | Did (NAME) receive a Hepatitis B vaccine – An injection in the thigh to prevent him/her from getting Hepatitis B within 24 hours after birth? | 0 = No  1 = Yes  9 = Don’t know | 0 = No  1 = Yes  9 = Don’t know | 0 = No  1 = Yes  9 = Don’t know |
| **4.18** | Has (NAME) ever received a pneumococcal vaccination – An injection in the thigh to protect him/her from getting some types of pneumonia? If yes, how many times was it received? | 0 = No  1 = Yes, \|__\| times  9 = Don’t know | 0 = No  1 = Yes, \|__\| times  9 = Don’t know | 0 = No  1 = Yes, \|__\| times  9 = Don’t know |
| **4.19** | Has (NAME) ever received a rotavirus vaccination – Drops in the mouth to protect him/her from getting some types of diarrhoea? If yes, how many times was it received? | 0 = No  1 = Yes, \|__\| times  9 = Don’t know | 0 = No  1 = Yes, \|__\| times  9 = Don’t know | 0 = No  1 = Yes, \|__\| times  9 = Don’t know |
| **4.20** | Has (NAME) ever received a measles vaccination – An injection in the arm at the age of 9 months or older to prevent him/her from getting measles? If yes, how many times was it received? | 0 = No  1 = Yes, \|__\| times  9 = Don’t know | 0 = No  1 = Yes, \|__\| times  9 = Don’t know | 0 = No  1 = Yes, \|__\| times  9 = Don’t know |
| **4.21** | The last time (NAME) passed stools, what was done to dispose of the stools? | 1 = Child used toilet / latrine  2 = Put / Rinsed into toilet or latrine  3 = Put / Rinsed into drain or ditch  4 = Thrown into garbage (solid waste)  5 = Buried  6 = Left in the open  7 = Other (*specify*____________________ )  9 = Don’t Know | 1 = Child used toilet / latrine  2 = Put / Rinsed into toilet or latrine  3 = Put / Rinsed into drain or ditch  4 = Thrown into garbage (solid waste)  5 = Buried  6 = Left in the open  7 = Other (*specify*____________________ )  9 = Don’t Know | 1 = Child used toilet / latrine  2 = Put / Rinsed into toilet or latrine  3 = Put / Rinsed into drain or ditch  4 = Thrown into garbage (solid waste)  5 = Buried  6 = Left in the open  7 = Other (*specify*____________________ )  9 = Don’t Know |

**5. Household member illnesses**

Now I would like to ask some questions about illness and treatments in your family (asked for all household members)

| # | # Number from Table 2 |  | \|__\| | \|__\| | \|__\| | \|__\| | \|__\| | \|__\| | \|__\| |
| --- | --- | --- | --- | --- | --- | --- | --- | --- | --- |
|  | Name from Table 2 |  |  |  |  |  |  |  |  |
|  | **Diarrhoea** |  |  |  |  |  |  |  |  |
|  | Has anyone in the household had diarrhoea in the last 2 weeks? | 1= Yes (Complete diarrhoea questions for each household member who experienced diarrhoea)  0 = No 🡪 Q5.12  9 = Don’t know 🡪 Q5.12 | \|__\| | \|__\| | \|__\| | \|__\| | \|__\| | \|__\| | \|__\| |
|  | Was there any blood in the stools? | 0 = No  1 = Yes  9 = Don’t know | \|__\| | \|__\| | \|__\| | \|__\| | \|__\| | \|__\| | \|__\| |
|  | Did you seek advice or treatment for the diarrhoea from any source? | 0 = No 🡪 Q 5.7  1 = Yes | \|__\| | \|__\| | \|__\| | \|__\| | \|__\| | \|__\| | \|__\| |
|  | From where did you seek advice or treatment?  *Probe:*  Anywhere else?  *Circle all providers mentioned, but do NOT prompt with any suggestions. Probe to identify each type of source.* | 1 = Govt. hospital  2 = PMC/polyclinic  3 = Commune health station  4 = Private hospital/clinic  5 = Pharmacy/drug store  6 = Shop  7 = Traditional practitioner  8 = Relative/friend  9 = Other (specify________________________) | 1  2  3  4  5  6  7  8  9  (______________) | 1  2  3  4  5  6  7  8  9  (______________) | 1  2  3  4  5  6  7  8  9  (______________) | 1  2  3  4  5  6  7  8  9  (______________) | 1  2  3  4  5  6  7  8  9  (______________) | 1  2  3  4  5  6  7  8  9  (______________) | 1  2  3  4  5  6  7  8  9  (______________) |
|  | *If more than one place mentioned:*  Where did you first seek advice or treatment? | \|__\| Code from above | \|__\| | \|__\| | \|__\| | \|__\| | \|__\| | \|__\| | \|__\| |
|  | How many days after the diarrhoea began did you first seek advice or treatment for (NAME)? | \|__\| Days 🡪 Q5.8 | \|__\| | \|__\| | \|__\| | \|__\| | \|__\| | \|__\| | \|__\| |

|  | Why did you not seek advice or treatment?  *Circle all mentioned* | 1 = Still waiting to see if it gets worse  2 = Episode was not serious  3 = Too far/no transport  4 = Too expensive/didn’t have money  5 = No one to look after other children  6 = Used home remedies  7 = No reason  8 = Other (specify ________________________) | 1  2  3  4  5  6  7  8 (______________) | 1  2  3  4  5  6  7  8 (______________) | 1  2  3  4  5  6  7  8 (______________) | 1  2  3  4  5  6  7  8 (______________) | 1  2  3  4  5  6  7  8 (______________) | 1  2  3  4  5  6  7  8 (______________) | 1  2  3  4  5  6  7  8 (______________) |
| --- | --- | --- | --- | --- | --- | --- | --- | --- | --- |
|  | At any time during the illness, was anything given to treat the diarrhoea? If yes, name all the treatments that were given.  *(Circle all mentioned)* | 0 = No, nothing was given 🡪 Q 5.10  *ORS:*  1 = ORS from a packet 🡪 Q 5.10  2 = Homemade sugar-salt solution 🡪 Q 5.10  3 = Other homemade fluid 🡪 Q 5.10  *Pill or syrup:*  4 = Antibiotic  5 = Antimotility 🡪 Q 5.10  6 = Zinc tablet 🡪 Q 5.10  7 = Other 🡪 Q 5.10  8 = Unknown pill or syrup 🡪 Q 5.10  *Injection:*  9 = Antibiotic  10 = Non-antibiotic 🡪 Q 5.10  11 = Unknown injection 🡪 Q 5.10  12 = IV fluids 🡪 Q 5.10  *Other:*  13=Home remedy/herbal medicine🡪Q 5.10  14 = Other (specify ______________________)🡪 Q 5.10 | 0  1  2  3  4  5  6  7  8  9  10  11  12  13  14  (______________) | 0  1  2  3  4  5  6  7  8  9  10  11  12  13  14  (______________) | 0  1  2  3  4  5  6  7  8  9  10  11  12  13  14  (______________) | 0  1  2  3  4  5  6  7  8  9  10  11  12  13  14  (______________) | 0  1  2  3  4  5  6  7  8  9  10  11  12  13  14  (______________) | 0  1  2  3  4  5  6  7  8  9  10  11  12  13  14  (______________) | 0  1  2  3  4  5  6  7  8  9  10  11  12  13  14  (________________) |
|  | *If antibiotic pill, syrup or injection were given:*  Where did you get this treatment from?  *(Circle all mentioned)* | 0 = Antibiotic not given (check 5.8 above)  1 = Govt. hospital  2 = PMC/polyclinic  3 = Commune health station  4 = Private hospital/clinic  5 = Pharmacy/drug store  6 = Shop  7 = Traditional practitioner  8 = Relative/friend  9 = Had it at home  10 = Other (specify_____________________) | 1  2  3  4  5  6  7  8  9  10  (______________) | 1  2  3  4  5  6  7  8  9  10  (______________) | 1  2  3  4  5  6  7  8  9  10  (______________) | 1  2  3  4  5  6  7  8  9  10  (______________) | 1  2  3  4  5  6  7  8  9  10  (______________) | 1  2  3  4  5  6  7  8  9  10  (______________) | 1  2  3  4  5  6  7  8  9  10  (________________) |
|  | Does (NAME) still have diarrhoea? | 0 = No  1 = Yes  9 = Don’t Know | \|__\| | \|__\| | \|__\| | \|__\| | \|__\| | \|__\| | \|__\| |
|  | Did anyone else in the household have diarrhoea in the last 2 weeks? | 0 = No 🡪 Q5.12  1 = Yes 🡪 Go back to Q5.1 for the next person who had diarrhoea | \|__\| | \|__\| | \|__\| | \|__\| | \|__\| | \|__\| | \|__\| |
|  | **Fever, cough, breathing difficulties** |  |  |  |  |  |  |  |  |
|  | Has (NAME) been ill with a fever at any time in the last 2 weeks? | 1 = Yes  0 = No  9 = DK | \|__\| | \|__\| | \|__\| | \|__\| | \|__\| | \|__\| | \|__\| |
|  | Has (NAME) had an illness with a cough at any time in the last 2 weeks? | 1 = Yes  2 = No cough but fever in Q5.12  *(Complete questions for each household member who experienced cough or fever)*  0 = No cough or fever 🡪 Q5.25  9 = Don’t Know 🡪 Q5.25 | \|__\| | \|__\| | \|__\| | \|__\| | \|__\| | \|__\| | \|__\| |
|  | When (NAME) had an illness with a cough, did he/she breathe faster than usual with short, rapid breaths or have difficulty breathing? | 0 = No 🡪 Q5.16  1 = Yes  9 = Don’t Know 🡪 Q5.16 | \|__\| | \|__\| | \|__\| | \|__\| | \|__\| | \|__\| | \|__\| |
|  | Was the fast or difficult breathing due to a problem in the chest or a blocked or runny nose? | 1 = Problem in chest only  2 = Blocked or runny nose only  3 = Both  4 = Other (specify_____________________)  9 = Don’t know | \|__\| | \|__\| | \|__\| | \|__\| | \|__\| | \|__\| | \|__\| |
|  | Did you seek advice or treatment for the illness from any source? | 0 = No 🡪Q5.21  1 = Yes  9 = Don’t Know 🡪 Q5.21 | \|__\| | \|__\| | \|__\| | \|__\| | \|__\| | \|__\| | \|__\| |
|  | From where did you seek advice or treatment?  *Probe:*  Anywhere else?  *Circle all providers mentioned, but do NOT prompt with any suggestions. Probe to identify each type of source.* | 1 = Govt. hospital  2 = PMC/polyclinic  3 = Commune health station  4 = Private hospital/clinic  5 = Pharmacy/drug store  6 = Shop  7 = Traditional practitioner  8 = Relative/friend  9 = Other (specify _____________________) | 1  2  3  4  5  6  7  8  9  (______________) | 1  2  3  4  5  6  7  8  9  (______________) | 1  2  3  4  5  6  7  8  9  (______________) | 1  2  3  4  5  6  7  8  9  (______________) | 1  2  3  4  5  6  7  8  9  (______________) | 1  2  3  4  5  6  7  8  9  (______________) | 1  2  3  4  5  6  7  8  9  (______________) |
|  | Where did you first seek advice or treatment? | Code from above\|__\| | \|__\| | \|__\| | \|__\| | \|__\| | \|__\| | \|__\| | \|__\| |
|  | How many days after the illness began did you first seek advice or treatment for (NAME)? | \|__\|__\| Days  99 = Don’t Know | \|__\|__\| Days | \|__\|__\| Days | \|__\|__\| Days | \|__\|__\| Days | \|__\|__\| Days | \|__\|__\| Days | \|__\|__\| Days |
|  | At any time during the illness, did (NAME) have blood taken for testing? | 0 = No  1 = Yes  9 = Don’t Know | \|__\| | \|__\| | \|__\| | \|__\| | \|__\| | \|__\| | \|__\| |
|  | Why did you not seek advice or treatment?  *Circle all mentioned* | 1 = Still waiting to see if it gets worse  2 = Episode was not serious  3 = Too far/no transport  4 = Too expensive/didn’t have money  5 = No one to look after other children  6 = Used home remedies  7 = No reason  8 = Other (specify ________________________) | 1  2  3  4  5  6  7  8 (______________) | 1  2  3  4  5  6  7  8 (______________) | 1  2  3  4  5  6  7  8 (______________) | 1  2  3  4  5  6  7  8 (______________) | 1  2  3  4  5  6  7  8 (______________) | 1  2  3  4  5  6  7  8 (______________) | 1  2  3  4  5  6  7  8 (______________) |
|  | At any time during the illness, was anything given to treat the illness? If yes, name all the treatments that were given.  *Circle all mentioned* | 0 = No, nothing was given 🡪 Q5.25  *Antibiotic drugs:*  1 = Pill/syrup  2= Injection  *Other drugs:*  3 = Aspirin 🡪 Q5.25  4 = Acetamiophen/paracetamol/Panadol  /Tylenol 🡪 Q5.25  5 = Ibuprofen 🡪 Q5.25  6 = Unknown pill or syrup 🡪 Q5.25  7 = Unknown injection 🡪 Q5.25  8 = Home remedy/herbal medicine 🡪 Q5.25  9 = Other (specify ______________)🡪Q5.25  10 = Don’t know 🡪 Q5.25 | 0  1  2  3  4  5  6  7  8  9  (_______)  10 | 0  1  2  3  4  5  6  7  8  9  (_______)  10 | 0  1  2  3  4  5  6  7  8  9  (_______)  10 | 0  1  2  3  4  5  6  7  8  9  (_______)  10 | 0  1  2  3  4  5  6  7  8  9  (_______)  10 | 0  1  2  3  4  5  6  7  8  9  (_______)  10 | 0  1  2  3  4  5  6  7  8  9  (_______)  10 |
|  | *If antibiotic pill, syrup or injection were given:*  Where did you get this treatment from?  *(Circle all mentioned)* | 0 = Antibiotic not given (check 5.22 above)  1 = Govt. hospital  2 = PMC/polyclinic  3 = Commune health station  4 = Private hospital/clinic  5 = Pharmacy/drug store  6 = Shop  7 = Traditional practitioner  8 = Relative/friend  9 = Had it at home  10 = Other (specify ___________________) | 0  1  2  3  4  5  6  7  8  9  10  (_______) | 0  1  2  3  4  5  6  7  8  9  10  (_______) | 0  1  2  3  4  5  6  7  8  9  10  (_______) | 0  1  2  3  4  5  6  7  8  9  10  (_______) | 0  1  2  3  4  5  6  7  8  9  10  (_______) | 0  1  2  3  4  5  6  7  8  9  10  (_______) | 0  1  2  3  4  5  6  7  8  9  10  (_______) |
|  | Is (NAME) still sick with a (fever/cough)? | 0 = No, neither  1 = Fever only  2 = Cough only  3 = Both fever and cough  9 = Don’t know | \|__\| | \|__\| | \|__\| | \|__\| | \|__\| | \|__\| | \|__\| |
|  | Has anyone else in the household had an illness with a cough at any time in the last 2 weeks? | 0 = No 🡪 Q5.26  1 = Yes 🡪 Go back to Q5.12 for the next person who had an illness with a cough | \|__\| | \|__\| | \|__\| | \|__\| | \|__\| | \|__\| | \|__\| |
|  | **Other illnesses and antibiotic use** | **(Complete all questions for each household member)** |  |  |  |  |  |  |  |
|  | Including the illnesses mentioned above, when was the last time (NAME) took antibiotics? | 1 = Currently taking  2 = Within the last 24 hours  3 = Within the last week  4 = Within the last 2 weeks  5 = Within the last month  6 = 2-3 months ago 🡪 Q5.31  7 = 4-5 months ago 🡪 Q5.31  8 = 6-11 months ago 🡪 Q5.32  9 = More than 12 months ago 🡪 Q5.32  0 = Never 🡪 Q5.32 | \|__\| | \|__\| | \|__\| | \|__\| | \|__\| | \|__\| | \|__\| |
|  | Did (NAME) ask for an antibiotic? If not, who recommended or prescribed that (NAME) take an antibiotic? | 0 = Yes, (NAME) asked for it  *Health worker at:*  1 = Hospital  2 = PMC/polyclinic  3 = Commune health station  *Other:*  4 = Pharmacist/chemist  5 = General store worker  6 = Drug store worker  7 = Market stall worker  8 = Traditional healer  9 = Friend/neighbour  10 = Relative  11 = Other  99 = Don’t Know | \|__\| | \|__\| | \|__\| | \|__\| | \|__\| | \|__\| | \|__\| |
|  | Where did (NAME) get the antibiotic?  *(Circle all mentioned)* | 1 = Govt. hospital  2 = PMC/polyclinic  3 = Commune health station  4 = Private hospital/clinic  5 = Pharmacy/drug store  6 = Shop  7 = Traditional practitioner  8 = Relative/friend  9 = Had it at home  10 = Other (specify_______________________) | 0  1  2  3  4  5  6  7  8  9  10  (_______) | 0  1  2  3  4  5  6  7  8  9  10  (_______) | 0  1  2  3  4  5  6  7  8  9  10  (_______) | 0  1  2  3  4  5  6  7  8  9  10  (_______) | 0  1  2  3  4  5  6  7  8  9  10  (_______) | 0  1  2  3  4  5  6  7  8  9  10  (_______) | 0  1  2  3  4  5  6  7  8  9  10  (_______) |
|  | How many days did (NAME) take it for? | \|__\|__\| Days  99 = Don’t know | \|__\|__\| Days | \|__\|__\| Days | \|__\|__\| Days | \|__\|__\| Days | \|__\|__\| Days | \|__\|__\| Days | \|__\|__\| Days |
|  | Why did (NAME) stop? | 1 = Completed the course  2 = Didn’t have enough money to buy the entire course  3 = The health worker/pharmacist/drug seller didn’t give me enough  4 = Didn’t buy enough or have enough at home  5 = Was told by a heath professional to stop  6 = Condition did not improve  7 = Condition improved  8 = There were side effects/medicine made him/her sick  9 = Don’t like to take medicines  10 = Didn’t think it was working  11 = Other (specify______________________)  12 = Don’t know | \|__\|__\| | \|__\|__\| | \|__\|__\| | \|__\|__\| | \|__\|__\| | \|__\|__\| | \|__\|__\| |
|  | How many times in the last 6 months has (NAME) taken antibiotics? | \|__\|__\| times | \|__\|__\| times | \|__\|__\| times | \|__\|__\| times | \|__\|__\| times | \|__\|__\| times | \|__\|__\| times | \|__\|__\| times |
|  | How many days has (NAME) been sick in the last month? | \|__\|__\| days | \|__\|__\| Days | \|__\|__\| Days | \|__\|__\| Days | \|__\|__\| Days | \|__\|__\| Days | \|__\|__\| Days | \|__\|__\| Days |
|  | How many days has (NAME) been absent from school or work because of this illness? | \|__\|__\| days | \|__\|__\| Days | \|__\|__\| Days | \|__\|__\| Days | \|__\|__\| Days | \|__\|__\| Days | \|__\|__\| Days | \|__\|__\| Days |
|  | In the last month did (NAME) go to or spend any time in a hospital for any reason? | 0 = No  1 = Yes, was admitted  2 = Yes, was the main carer for a patient  3 = Yes, visited a patient  4 = Yes, work in a hospital | 0  1  2  3  4 | 0  1  2  3  4 | 0  1  2  3  4 | 0  1  2  3  4 | 0  1  2  3  4 | 0  1  2  3  4 | 0  1  2  3  4 |
|  | Go back to Q5.26 and complete other illness questions for each household member |  |  |  |  |  |  |  |  |

**6. Access to health care and knowledge about antibiotics and antibiotic resistance**

Now I would like to ask you some questions about how your family accesses healthcare, and what you know about different drugs

| **#** | **Question** | **Responses** | | | |
| --- | --- | --- | --- | --- | --- |
| **6.1** | Which is your closest health care provider? | ____________________________________________Name | | | |
|  | Which of these healthcare providers do members of your household mostly go to if they are *mildly* ill, for example with a cough or cold?  *(Circle all mentioned)* | 0 = Don’t usually seek care for mild illness 🡪Q6.3  1 = Govt. hospital  2 = PMC/polyclinic  3 = Commune health station  4 = Private hospital/clinic  5 = Pharmacy/drug store  6 = Shop  7 = Traditional practitioner  8 = Relative/friend  9 = Other(specify_____________________________________________) | | | |
| **6.2** | Which of these do you usually go to first? | \|__\| code from above | | | |
| **6.3** | How do you usually travel to get to your commune health station? | 1 = Walking  2 = Bicycle  3 = Scooter/motorbike  4 = Car  5 = Bus  6 = Other (specify ____________________________________________) | | | |
| **6.4** | How long does it take to get to the commune heath station using this transport? | \|__\|__\| minutes | | | |
| **6.5** | Do you know what bacteria are? If yes, do you know any illnesses that are usually caused by bacteria?  *(Circle all mentioned)* | 0 = No, don’t know what bacteria are  1 = Pneumonia  2 = Food poisoning  3 = Dysentery/bloody diarrhoea  4 = Urinary tract infection  5 = Coughs and colds  6 = Flu  7 = Know what bacteria are, but don’t know any illnesses caused by them  8 = Other (specify ____________________________________________) | | | |
| **6.6** | Do you think there are any negative effects of taking different types of antibiotics? If yes, what are they? | 0 = No, don’t think there are any negative effects  1 = Side effects (e.g. diarrhoea, skin sensitivity, organ damage)  2 = Can contribute to development of antibiotic resistance  3 = Can kill off “good bacteria” and have long term effects on health  4 = Other (specify ____________________________________________)  5 = There are negative effects but don’t know what they are | | | |
| **6.7** | Some antibiotic medicines that used to work in fighting infections no longer work. This problem is called antibiotic resistance. Have you heard of this problem before? | 0 = No 🡪End  1 = Yes  9 = Don’t know 🡪End | | | |
| **6.8** | Where did you learn about antibiotic resistance?  *(Circle all mentioned)* | 1 = Health worker  2 = Radio  3 = TV  4 = Newspaper/magazine  5 = Internet  6 = Social media  7 = Community organisation(e.g. women’s union, farmer’s union)  8 = Other community group  9 = Friend/relative  10 = Other (specify ___________________________________________) | | | |
| **6.9** | What could the consequences of getting an antibiotic resistant infection be?  *(Circle all mentioned)* | 1 = Be sick for longer  2 = May have to visit doctor more or be treated in hospital  3 = May need more expensive medicine that may cause side-effects  4 = Other (specify __________________________________) | | | |
| **6.10** | Can you think of any ways of reducing the problem of antibiotic resistance?  *(Tick those spontaneously mentioned in the first column)*  Now I am going to mention some behaviours and I want you to say whether they are good or bad ways of reducing the problem of antibiotic resistance.  *(Tick those recognised after prompting in the GOOD or BAD columns)* | Mentioned spontaneously | Recognised as a GOOD way of reducing antibiotic resistance after prompting | Recognised as a BAD way of reducing antibiotic resistance after prompting | Don’t know after prompting |
|  | 1 = Don’t take antibiotics when they are not needed (e.g. for colds and sore throats) |  |  |  |  |
|  | 2 = Don’t demand antibiotics from health workers or drug suppliers |  |  |  |  |
|  | 3 = Make sure antibiotics are good quality and within expiry date |  |  |  |  |
|  | 4 = Complete the course as recommended by a health worker |  |  |  |  |
|  | 5 = Don’t use antibiotics prescribed for someone else |  |  |  |  |
|  | 6 = Make sure you use the right antibiotic for the right infection |  |  |  |  |
|  | 7 = Make sure you take antibiotics as soon as you feel sick |  |  |  |  |
|  | 8 = Make sure you take a very strong antibiotic to kill the infection |  |  |  |  |
|  | 9 = Take several different antibiotics to make sure the infection is killed |  |  |  |  |
|  | 10 = Don’t use antibiotics in animal feed as a growth promoter |  |  |  |  |
|  | 11 = Washing hands after contact with a live animal, slaughtering animals or preparing meat |  |  |  |  |
|  | 12 = Washing hands after contact with someone or something that has been touched by a person who has an antibiotic-resistant infection |  |  |  |  |

Time of finishing interview |__|__|:|__|__|

**Supplementary file 2**: List of curated variables

| **Variable** | **Levels** | **Description** |
| --- | --- | --- |
| Carbapenem resistant Enterobacterales | No  Yes | Colonisation with CRE and C3GRE was defined as the growth of lactose-fermenting pink large smooth colonies on the meropenem (0.5 mg/L) or ceftazidime (2.0 mg/L) supplemented MacConkey agar plates, respectively. |
| Third generation cephalosporin resistant Enterobacterales | No  Yes |  |
| Age | - | Age in years based on the difference between the date of the household interview (see section 1 of the survey; supplementary file 1) and the birth date (see question 2.2 of the survey; supplementary file 1). |
| Sex | Female  Male | Sex is based on the self-reported sex (see question 2.2 of the survey; supplementary file 1). Representatives of each household were asked to answer this question for all households. Variable was only analysed for individuals > 16 years-of-age. |
| Education | College/university  Professional school  Upper secondary  Lower secondary  Primary and lower  No education  Unknown | Educational level based on the self-reported, highest level of education (see question 2.5 of the survey; supplementary file 1). Representatives of each household were asked to answer this question for all households. Variable was only analysed for individuals > 16 years-of-age. |
| Occupation | Farmer  Labourer  Factory worker  Office worker  Shop/retail/hospitality  Other work  Student  No work/unknown | Occupational level based on the self-reported current occupation (see question 2.9 of the survey; supplementary file 1). Representatives of each household were asked to answer this question for all households. Variable was only analysed for individuals > 16 years-of-age. |
| Socio-economic status (SES) | Low income  Middle income  High income | Cumulative score of principal component analysis (PCA) of direct observations of general assets and house construction (i.e.. flooring material, roofing material, wall material, crowding (number of people per sleeping room), ownership of electricity, radio, television, telephone, mobile phone, refrigerator, bed, table and chair set, sofa, computer, tablet or iPad, fan, air conditioner, gas cooker, electric cooker, washing machine, bicycle, motorcycle, tractor, car or truck, ship or boat; see section 3 of the survey; supplementary file 1). Factors that may have had a direct effect on colonisation with resistant bacteria were excluded from the PCA (i.e.., handwashing places, availability of soap, type of toilet, water source, ownership of animals). Scores were subdivided into three wealth terciles. |
| Illness ^A^ | No and unknown  Yes | “Yes” for participants that reported to suffer from diarrhoea, cough and/or fever in the two weeks prior or during the survey interview. “No and unknown” for all other (see section 5 of the survey; supplementary file 1) |
| Antibiotic use 4 weeks ^A^ | No and unknown  Yes | “Yes” for participants that reported using antibiotics 4 weeks before or during the survey interview. “No and unknown” for all other (see question 5.26 of the survey; supplementary file1). |
| Sampling period | Period 1 (Jul-Sep)  Period 2 (Nov-Dec  Period 3 (Mar-Apr) | Sampling throughout the study was subdivided into three distinct periods: (1) Period 1 between July and September 2018, (2) Period 2 between November and December 2018, and (3) Period 3 between March and April 2019. There was no sampling for >1 month between each sampling period. |
| Owning animals | No  Yes | “Yes” for participants that reported to have at least one animal (i.e., cattle, horses, donkeys, mules, goats, chicken, quails, pigs, ducks, geese, swans, fish, and/or pet animals) (see question 3.16 of the survey; supplementary file 1). |
| Antibiotic use in animals | No animals  Animals and antibiotic use  Animals and no antibiotic use | Based on “owning animals” and self-reported use of antibiotics in animals (see questions 3.16 and 3.17 of the survey; supplementary file 1). |
| Owning livestock | No livestock  1 species  2 species or more | Based on the “owning animals” variables with cattle, horses, donkeys, mules, goats, chicken, quails, pigs, ducks, geese, swans, and fish considered as livestock. |
| Beef consumption | Less than once per week  Once per week  More than once per week | Food consumption was based on the self-reported consumption of selected food type (see question 3.30 of the survey; supplementary file 1). Representatives of each household were asked to indicate how frequently they ate each subtype with the following 5 answer options: never, less than once per week, once per week, more than once per week, or every day. |
| Chicken consumption | Less than once per week  Once per week  More than once per week |  |
| Tofu consumption | Once per week and less  More than once per week |  |
| Fermented food consumption | Once per week and less  More than once per week |  |
| Drinking water source | Improved source  Rainwater | “Rainwater” for households that reported exclusive use of rainwater as a main drinking water source, “Improved” for households using any additional/other source (see question 3.19 of the survey; supplementary file 1). |
| Water Sanitation and Hygiene (WASH) | Worse  Middle  Better | The WASH condition was based on three factors: (1) using rainwater as a source of drinking water, (2) boiling water before consumption, (3) having a flushed toilet. “Better” were all households that met these three criteria, “middle” when one was missing, and “worse” for all other households. |
| Handwashing | Often  Not often | The handwashing behaviour was determined for several activities and scored as never (0 points), sometimes (1 point), most of the time (2 points), always (3 points). The mean score was calculated for 7 activities (i.e., after toilet, before cooking, after cooking, before eating, after eating, after handling animals and after sneezing or coughing). Mean scores equal and higher than 1.5 were considered as “often”. |
| Hospital exposure ^A^ | No  Yes | “Yes” for participants that reported to have been admitted, visited, or worked at a hospital within the last month before the survey interview. |
| Delivery | Natural delivery  Caesarean section | “Caesarean section” for participants that reported a child was delivered through caesarean section (see question 4.1 of the survey; supplementary file 1), “Natural delivery” for the remaining participants under the age of 5. |
| Hospitalisation | Less than 1 day  One day or more | Based on questions 4.3 and 4.4 (see supplementary file 1). |
| Early breastfed | Yes  No | “Yes” for children under 5 years-of-age who were breastfed within the first hour after delivery and “No” for all other children. This cut-off was based on WHO’s guidelines for early initiation of breastfeeding. |
| Exclusive breastfed 4 months | Yes  No | “Yes” for children under 5 years-of-age who were exclusively breastfed with milk until the age of 4 months, and “No” for all other children. |
| Liquids first 3 days | Only mother milk  Other milk / instant formula | “Yes” for children under 5 years-of-age who were exclusively breastfed with milk during the first 3 days, and “No” for all other children. |
| Fully vaccinated | Yes  No | As per schedule of the national expanded program on immunisation (EPI) in Vietnam. Children were considered fully vaccinated when:   - Younger than 2 months: BCG + HepB - Between 2 and 3 months: above + 1 OPV + 1 pentavalent - Between 3 and 4 months: above + 1 OPV + 1 pentavalent - Between 4 and 9 months: above + 1 OPV + 1 pentavalent - Between 9 and 18 months: above + 1 MMR - Older than 18 months: above + 1 MMR   Fully vaccinated children (at the age of 18 months) hence received 1 BCG, 1 HepB, 3 OPV, 3 pentavalent and 2 MMR vaccines. |

^A^ The CRE analysis was only performed on a household level due to the low number of CRE carriers. If one of the household members was positive for these factors, the household was considered as suffering from illness and/or using antibiotics.

**Supplementary file 3**: Overview of CRE and C3GRE carriage rates stratified by village

|  |  | CRE | | | C3GRE | | |
| --- | --- | --- | --- | --- | --- | --- | --- |
| Village | N | N (missing) | Absolute | Percentage | N (missing) | Absolute | Percentage |
| 001 | 53 | 53 (0) | 1 | 1.9% | 53 (0) | 51 | 96.2% |
| 002 | 57 | 57 (0) | 0 | 0.0% | 57 (0) | 55 | 96.5% |
| 003 | 49 | 22 (27) | 1 | 4.6% | 48 (1) | 48 | 100.0% |
| 004 | 129 | 79 (50) | 0 | 0.0% | 92 (37) | 85 | 92.4% |
| 005 | 152 | 152 (0) | 0 | 0.0% | 152 (0) | 150 | 98.7% |
| 006 | 43 | 43 (0) | 0 | 0.0% | 43 (0) | 43 | 100.0% |
| 007 | 67 | 21 (46) | 0 | 0.0% | 58 (9) | 54 | 93.1% |
| 008 | 101 | 77 (24) | 1 | 1.3% | 77 (24) | 69 | 89.6% |
| 011 | 137 | 137 (0) | 0 | 0.0% | 137 (0) | 128 | 93.4% |
| 012 | 69 | 69 (0) | 0 | 0.0% | 69 (0) | 64 | 92.8% |
| 013 | 124 | 100 (24) | 2 | 2.0% | 117 (7) | 107 | 91.5% |
| 014 | 71 | 66 (5) | 0 | 0.0% | 66 (5) | 62 | 94.0% |
| 015 | 70 | 70 (0) | 0 | 0.0% | 70 (0) | 59 | 84.3% |
| 016 | 80 | 62 (18) | 2 | 3.2% | 78 (2) | 70 | 89.7% |
| 017 | 107 | 82 (25) | 10 | 12.2% * | 103 (4) | 101 | 98.1% |
| 018 | 28 | 28 (0) | 0 | 0.0% | 28 (0) | 27 | 96.4% |
| 019 | 26 | 0 (26) | 0 | 0.0% | 22 (4) | 21 | 95.5% |
| 020 | 38 | 0 (38) | 0 | 0.0% | 31 (7) | 27 | 87.1% |
| 021 | 97 | 17 (80) | 0 | 0.0% | 17 (80) | 12 | 70.6% |
| **Total** | **1498** | **1135 (363)** | **17** | **1.5%** | **1318 (180)** | **1233** | **93.6%** |

* The CRE proportion was significantly higher in V017 than in V001 (Fisher exact, p = 0.049), V008 (p = 0.010), and V013 (p = 0.007). The proportion in V017 was not statistically different from the CRE proportion in V003 (p = 0.450) and V016 (p = 0.069).

**Supplementary file 4**: Complete list of the CRE household analyses (weighted)

Model 1 – Weighted crude model (OR)

Model 2 – Weighted crude model + wealth (aOR)

|  | Total | | CRE carrier | | MODEL 1 | | MODEL 2 | |
| --- | --- | --- | --- | --- | --- | --- | --- | --- |
|  | N | % | N | % | OR  [95% CI] | p-value | OR  [95% CI] | p-value |
| **Economic status** |  |  |  |  |  |  |  |  |
| Low- and middle-income | 161 | 65.7% | 3 | 1.9% |  |  |  |  |
| High-income | 84 | 34.3% | 9 | 10.7% | 3.93 [0.76 - 20.35] | 0.105 |  |  |
| **Household AMU 4 weeks** |  |  |  |  |  |  |  |  |
| No | 162 | 66.1% | 9 | 5.6% |  |  |  |  |
| Yes | 83 | 33.9% | 3 | 3.6% | 0.54 [0.09 - 3.37] | 0.506 | 0.60 [0.09 - 4.11] | 0.604 |
| **Household illness** |  |  |  |  |  |  |  |  |
| No | 183 | 74.7% | 10 | 5.5% |  |  |  |  |
| Yes | 62 | 25.3% | 2 | 3.2% | 0.79 [0.11 - 5.75] | 0.812 | 1.11 [0.13 - 9.20] | 0.924 |
| **Hospital exposure ^c^** |  |  |  |  |  |  |  |  |
| No | 242 | 98.8% | 12 | 5.0% |  |  |  |  |
| Yes | 3 | 1.2% | 0 | 0.0% | null | **<0.001 ***** | null | **<0.001 ***** |
| **Seasonality** |  |  |  |  |  |  |  |  |
| Spring and summer 1 | 207 | 84.5% | 2 | 1.0% |  |  |  |  |
| Autumn and winter 2 | 38 | 15.5% | 10 | 26.3% | 29.47 [3.92 - 221.60] | 0.001 | 27.77 [3.43 - 255.01] | 0.002 |
| **Period of sampling** |  |  |  |  |  |  |  |  |
| Period 1 | 122 | 49.8% | 10 | 8.2% |  |  |  |  |
| Period 2 | 22 | 9.0% | 1 | 4.5% | 0.13 [0.01 - 1.14] | 0.067 | 0.11 [0.01 - 0.99] | 0.050 |
| Period 3 | 101 | 41.2% | 1 | 1.0% | 0.25 [0.03 - 2.18] | 0.210 | 0.29 [0.03 - 2.51] | 0.265 |
| **Abx use in animals** |  |  |  |  |  |  |  |  |
| No animals | 108 | 44.1% | 1 | 0.9% |  |  |  |  |
| Animals and antibiotic | 95 | 38.8% | 6 | 6.3% | 2.08 [0.20 - 21.93] | 0.544 | 2.45 [0.22 - 27.14] | 0.465 |
| Animals and no antibiotic | 42 | 17.1% | 5 | 11.9% | 11.34 [1.14 - 112.91] | 0.040 | 12.45 [1.19 - 129.88] | 0.036 |
| **Owning animals** |  |  |  |  |  |  |  |  |
| No | 108 | 44.1% | 1 | 0.9% |  |  |  |  |
| Yes | 137 | 55.9% | 11 | 8.0% | 4.52 [0.52 - 39.32] | 0.172 | 5.31 [0.56 - 50.06] | 0.146 |
| **Owning livestock** |  |  |  |  |  |  |  |  |
| No | 122 | 49.8% | 2 | 1.6% |  |  |  |  |
| Yes | 123 | 50.2% | 10 | 8.1% | 2.31 [0.43 - 14.50] | 0.333 | 3.03 [0.50 - 18.22] | 0.228 |
| **Owning dogs** |  |  |  |  |  |  |  |  |
| No | 158 | 64.5% | 3 | 1.9% |  |  |  |  |
| Yes | 87 | 35.5% | 9 | 10.3% | 1.81 [0.38 - 8.47] | 0.455 | 1.76 [0.40 - 7.79] | 0.456 |
| **Owning pigs** |  |  |  |  |  |  |  |  |
| No | 204 | 83.3% | 10 | 4.9% |  |  |  |  |
| Yes | 41 | 16.7% | 2 | 4.9% | 0.25 [0.05 - 1.31] | 0.102 | 0.29 [0.05 - 1.61] | 0.158 |
| **Owning chickens** |  |  |  |  |  |  |  |  |
| No | 135 | 55.1% | 4 | 3.0% |  |  |  |  |
| Yes | 110 | 44.9% | 8 | 7.3% | 1.24 [0.27 - 5.78] | 0.785 | 1.44 [0.28 - 7.52] | 0.665 |
| **Owning ducks** |  |  |  |  |  |  |  |  |
| No | 227 | 92.7% | 10 | 4.4% |  |  |  |  |
| Yes | 18 | 7.3% | 2 | 11.1% | 0.67 [0.12 - 3.81] | 0.653 | 1.42 [0.21 - 9.51] | 0.720 |
| **Owning fish** |  |  |  |  |  |  |  |  |
| No | 239 | 97.6% | 11 | 4.6% |  |  |  |  |
| Yes | 6 | 2.4% | 1 | 16.7% | 0.92 [0.08 - 10.29] | 0.948 | 0.86 [0.05 - 13.95] | 0.913 |
| **Livestock owned 2** |  |  |  |  |  |  |  |  |
| No livestock | 122 | 49.8% | 2 | 1.6% |  |  |  |  |
| One species | 75 | 30.6% | 7 | 9.3% | 3.56 [0.62 - 20.60] | 0.157 | 4.22 [0.67 - 26.69] | 0.127 |
| 2 or more species | 48 | 19.6% | 3 | 6.3% | 0.56 [0.09 - 3.54] | 0.539 | 0.86 [0.12 - 6.18] | 0.878 |
| **Chicken consumption** |  |  |  |  |  |  |  |  |
| Less than once per week | 150 | 61.2% | 5 | 3.3% |  |  |  |  |
| Once per week | 35 | 14.3% | 1 | 2.9% | 1.62 [0.16 - 16.13] | 0.681 | 1.50 [0.15 - 14.57] | 0.729 |
| More than once per week | 60 | 24.5% | 6 | 10.0% | 1.58 [0.29 - 8.48] | 0.594 | 1.99 [0.35 - 11.21] | 0.434 |
| **Pork consumption** |  |  |  |  |  |  |  |  |
| Once per week and less | 78 | 31.8% | 1 | 1.3% |  |  |  |  |
| More than once per week | 167 | 68.2% | 11 | 6.6% | 3.09 [0.35 - 26.92] | 0.308 | 2.47 [0.29 - 20.75] | 0.405 |
| **Beef consumption** |  |  |  |  |  |  |  |  |
| Less than once per week | 163 | 66.5% | 7 | 4.3% |  |  |  |  |
| Once per week | 37 | 15.1% | 2 | 5.4% | 2.01 [0.5 - 16.11] | 0.510 | 1.61 [0.22 - 11.83] | 0.638 |
| More than once per week | 45 | 18.4% | 3 | 6.7% | 1.83 [0.27 - 12.56] | 0.540 | 2.06 [0.31 - 13.55] | 0.455 |
| **Other type of meat** |  |  |  |  |  |  |  |  |
| Less than once per week | 198 | 80.8% | 9 | 4.5% |  |  |  |  |
| Once a week or more | 47 | 19.2% | 3 | 6.4% | 0.39 [0.09 - 1.67] | 0.207 | 0.65 [0.13 - 3.16] | 0.590 |
| **Shrimp consumption** |  |  |  |  |  |  |  |  |
| Less than once per week | 116 | 47.3% | 8 | 6.9% |  |  |  |  |
| Once per week | 87 | 35.5% | 1 | 1.1% | 0.52 [0.05 - 4.91] | 0.565 | 0.59 [0.05 - 6.82] | 0.670 |
| More than once per week | 42 | 17.1% | 3 | 7.1% | 2.40 [0.42 - 13.66] | 0.326 | 3.97 [0.72 - 21.97] | 0.116 |
| **Seafood consumption** |  |  |  |  |  |  |  |  |
| Less than once per week | 123 | 50.2% | 7 | 5.7% |  |  |  |  |
| Once per week | 64 | 26.1% | 2 | 3.1% | 2.21 [0.35 - 14.16] | 0.403 | 1.79 [0.25 - 14.62] | 0.562 |
| More than once per week | 58 | 23.7% | 3 | 5.2% | 2.37 [0.39 - 14.31] | 0.349 | 2.36 [0.42 - 13.08] | 0.328 |
| **Tofu consumption** |  |  |  |  |  |  |  |  |
| Once per week and less | 60 | 24.5% | 5 | 8.3% |  |  |  |  |
| More than once per week | 185 | 75.5% | 7 | 3.8% | 0.42 [0.09 - 2.08] | 0.291 | 0.39 [0.08 - 2.02] | 0.264 |
| **Dairy consumption** |  |  |  |  |  |  |  |  |
| Not every day | 96 | 39.2% | 4 | 4.2% |  |  |  |  |
| Everyday | 149 | 60.8% | 8 | 5.4% | 0.84 [0.18 - 3.91] | 0.824 | 0.65 [0.15 - 2.90] | 0.576 |
| **Fermented food** |  |  |  |  |  |  |  |  |
| Once per week and less | 169 | 69.0% | 10 | 5.9% |  |  |  |  |
| More than once per week | 76 | 31.0% | 2 | 2.6% | 0.59 [0.08 - 4.31] | 0.607 | 0.61 [0.09 - 4.28] | 0.619 |
| **Indoor smoking** |  |  |  |  |  |  |  |  |
| No | 170 | 69.4% | 6 | 3.5% |  |  |  |  |
| Yes | 75 | 30.6% | 6 | 8.0% | 0.86 [0.18 - 4.21] | 0.851 | 1.17 [0.22 - 6.18] | 0.857 |
| **Drinking water** |  |  |  |  |  |  |  |  |
| Improved source | 157 | 64.1% | 7 | 4.5% |  |  |  |  |
| Rainwater only | 88 | 35.9% | 5 | 5.7% | 3.96 [0.85 - 18.47] | 0.081 | 4.02 [0.85 - 19.14] | 0.082 |
| **Drinking water 2** |  |  |  |  |  |  |  |  |
| Improved source | 94 | 38.4% | 5 | 5.3% |  |  |  |  |
| Piped supply | 151 | 61.6% | 7 | 4.6% | 0.29 [0.06 - 1.36] | 0.118 | 0.28 [0.06 - 1.30] | 0.105 |
| **Water treatment** |  |  |  |  |  |  |  |  |
| Additional treatments | 40 | 16.3% | 2 | 5.0% |  |  |  |  |
| Boiling only | 205 | 83.7% | 10 | 4.9% | 3.69 [0.70 - 19.35] | 0.124 | 5.07 [0.93 - 27.57] | 0.061 |
| **WASH combined ternary** |  |  |  |  |  |  |  |  |
| Better condition | 86 | 35.1% | 5 | 5.8% |  |  |  |  |
| Middle condition | 107 | 43.7% | 5 | 4.7% | 0.27 [0.05 - 1.47] | 0.132 | 0.27 [0.05 - 1.47] | 0.132 |
| Worse condition | 52 | 21.2% | 2 | 3.8% | 0.12 [0.02 - 0.71] | 0.020 | 0.10 [0.02 - 0.61] | 0.013 |
| **WASH combined binary** |  |  |  |  |  |  |  |  |
| Better condition | 86 | 35.1% | 5 | 5.8% |  |  |  |  |
| Middle and worse condition | 159 | 64.9% | 7 | 4.4% | 0.23 [0.05 - 1.08] | 0.063 | 0.22 [0.05 - 1.03] | 0.056 |
| **Washing hands** |  |  |  |  |  |  |  |  |
| Rarely (< 1) | 42 | 17.1% | 2 | 4.8% |  |  |  |  |
| Sometimes (> 1) | 86 | 35.1% | 2 | 2.3% | 1.02 [0.10 - 10.01] | 0.989 | 0.90 [0.10 - 7.86] | 0.928 |
| Most of the times (> 1.5) | 117 | 47.8% | 8 | 6.8% | 1.25 [0.15 - 10.08] | 0.835 | 0.73 [0.08 - 6.74] | 0.780 |
| **Washing hands 2** |  |  |  |  |  |  |  |  |
| Not often | 128 | 52.2% | 4 | 3.1% |  |  |  |  |
| Often | 117 | 47.8% | 8 | 6.8% | 1.24 [0.27 - 5.76] | 0.787 | 0.83 [0.14 - 5.09] | 0.782 |
| **After toilet** |  |  |  |  |  |  |  |  |
| Not often | 94 | 38.4% | 4 | 4.3% |  |  |  |  |
| Often | 151 | 61.6% | 8 | 5.3% | 0.62 [0.13 - 2.91] | 0.550 | 0.39 [0.06 - 2.28] | 0.295 |
| **Before cooking** |  |  |  |  |  |  |  |  |
| Not often | 135 | 55.1% | 4 | 3.0% |  |  |  |  |
| Often | 110 | 44.9% | 8 | 7.3% | 1.38 [0.30 - 6.45] | 0.680 | 1.09 [0.21 - 5.82] | 0.918 |
| **Before eating** |  |  |  |  |  |  |  |  |
| Not often | 137 | 55.9% | 4 | 2.9% |  |  |  |  |
| Often | 108 | 44.1% | 8 | 7.4% | 1.56 [0.34 - 7.30] | 0.570 | 1.13 [0.19 - 6.86] | 0.893 |
| **After eating** |  |  |  |  |  |  |  |  |
| Not often | 128 | 52.2% | 4 | 3.1% |  |  |  |  |
| Often | 117 | 47.8% | 8 | 6.8% | 1.24 [0.27 - 5.76] | 0.787 | 0.96 [0.19 - 4.91] | 0.957 |
| **After cleaning baby** |  |  |  |  |  |  |  |  |
| Not often | 49 | 20.0% | 1 | 2.0% |  |  |  |  |
| Often | 153 | 62.4% | 7 | 4.6% | 8.18 [0.81 - 82.58] | 0.076 | 4.38 [0.39 - 49.66] | 0.234 |
| No baby / unknown | 43 | 17.6% | 4 | 9.3% | 24.48 [2.60 - 230.83] | 0.006 | 14.48 [1.28 - 163.46] | 0.032 |
| **Before feeding baby** |  |  |  |  |  |  |  |  |
| Not often | 80 | 32.7% | 1 | 1.3% |  |  |  |  |
| Often | 120 | 49.0% | 7 | 5.8% | 14.52 [1.44 - 146.05] | 0.024 | 9.97 [0.99 - 100.33] | 0.052 |
| No baby / unknown | 45 | 18.4% | 4 | 8.9% | 30.65 [3.28 - 286.56] | 0.003 | 21.22 [2.09 - 215.68] | 0.010 |
| **After handling animals** |  |  |  |  |  |  |  |  |
| Not often | 80 | 32.7% | 3 | 3.8% |  |  |  |  |
| Often | 165 | 67.3% | 9 | 5.5% | 2.06 [0.33 - 12.96] | 0.441 | 1.38 [0.16 - 11.59] | 0.767 |
| **After sneezing** |  |  |  |  |  |  |  |  |
| Not often | 222 | 90.6% | 10 | 4.5% |  |  |  |  |
| Often | 23 | 9.4% | 2 | 8.7% | 2.39 [0.31 - 23.49] | 0.403 | 2.70 [0.31 - 23.49] | 0.369 |
| **Family strata** |  |  |  |  |  |  |  |  |
| O5 household | 91 | 37.1% | 5 | 5.5% |  |  |  |  |
| U5 household | 154 | 62.9% | 7 | 4.5% | 0.76 [0.16 - 3.52] | 0.722 | 0.70 [0.16 - 3.18] | 0.649 |

^c^ Households were considered hospital exposed when at least one member visited. was admitted. or worked at a hospital in the last month.

**Supplementary file 5**: Complete list of the C3GRE individual analyses (weighted)

Model 1 – Weighted crude model (OR)

Model 2 – Weighted crude model + wealth + age category (aOR)

Model 3 – Weighted crude model + wealth + age category + AMU (mediating effect)

Model 4 – Weighted crude model + wealth + age category + illness (mediating effect)

Model 5 – Weighted crude model + wealth + age category + WASH combined (mediating effect)

|  | Total | | C3GRE carrier | | MODEL 1 | | MODEL 2 | | MODEL 3 | | MODEL 4 | | MODEL 5 | |
| --- | --- | --- | --- | --- | --- | --- | --- | --- | --- | --- | --- | --- | --- | --- |
|  | N | % | N | % | OR  95% CI | p-value | OR  95% CI | p-value | OR  95% CI | p-value | *OR  95% CI* | *p-value* | OR  95% CI | p-value |
| **Economic status** |  |  |  |  |  |  |  |  |  |  |  |  |  |  |
| Low income | 453 | 34.4% | 434 | 95.8% |  |  |  |  |  |  |  |  |  |  |
| Middle class | 423 | 32.1% | 389 | 92.0% | 0.46 [0.20 - 1.05] | 0.066 |  |  |  |  |  |  |  |  |
| Wealthy | 442 | 33.5% | 410 | 92.8% | 0.29 [0.12 - 0.74] | 0.010 |  |  |  |  |  |  |  |  |
| **Age category** |  |  |  |  |  |  |  |  |  |  |  |  |  |  |
| Over 5 years-of-age | 1107 | 84.0% | 1046 | 94.5% |  |  |  |  |  |  |  |  |  |  |
| Under 5 years-of-age | 211 | 16.0% | 187 | 88.6% | 0.69 [0.37 - 1.28] | 0.240 |  |  |  |  |  |  |  |  |
| **Ilness** |  |  |  |  |  |  |  |  |  |  |  |  |  |  |
| No and unknown | 1215 | 92.2% | 1135 | 93.4% |  |  |  |  |  |  |  |  |  |  |
| Yes | 103 | 7.8% | 98 | 95.1% | 1.45 [0.34 - 6.21] | 0.616 | 1.48 [0.34 - 6.51] | 0.603 | 1.43 [0.33 - 6.17] | 0.631 |  |  | 1.53 [0.33 - 7.02] | 0.583 |
| **Antibiotic use 2 weeks** |  |  |  |  |  |  |  |  |  |  |  |  |  |  |
| No and unknown | 1209 | 91.7% | 1129 | 93.4% |  |  |  |  |  |  |  |  |  |  |
| Yes | 109 | 8.3% | 104 | 95.4% | 1.10 [0.32 – 3.74] | 0.880 | 1.13 [0.33 – 3.95] | 0.843 |  |  | 0.89 [0.24 – 3.35] | 0.865 | 1.21 [0.31 – 4.79] | 0.785 |
| **Antibiotic use 4 weeks** |  |  |  |  |  |  |  |  |  |  |  |  |  |  |
| No and unknown | 1150 | 87.3% | 1076 | 93.6% |  |  |  |  |  |  |  |  |  |  |
| Yes | 168 | 12.7% | 157 | 93.5% | 1.15 [0.44 - 3.02] | 0.774 | 1.22 [0.45 - 3.31] | 0.696 |  |  | 1.05 [0.41 - 2.71] | 0.919 | 1.42 [0.48 - 4.22] | 0.531 |
| **Hospital exposure** |  |  |  |  |  |  |  |  |  |  |  |  |  |  |
| No and unknown | 1313 | 99.6% | 1228 | 93.4% |  |  |  |  |  |  |  |  |  |  |
| Yes | 5 | 0.4% | 5 | 100% | Inf | <0.001 | Inf | <0.001 | Inf | <0.001 | Inf | <0.001 | Inf | <0.001 |
| **Seasonality** |  |  |  |  |  |  |  |  |  |  |  |  |  |  |
| Spring and summer 1 | 962 | 73.0% | 889 | 92.4% |  |  |  |  |  |  |  |  |  |  |
| Autumn and winter 2 | 356 | 27.0% | 344 | 96.6% | 2.87 [1.11 - 7.42] | 0.031 | 3.17 [1.12 - 8.99] | 0.031 | 3.15 [1.10 - 9.07] | 0.034 | 3.16 [1.12 - 8.95] | 0.031 | 3.45 [1.26 - 9.41] | 0.016 |
| **Period of sampling** |  |  |  |  |  |  |  |  |  |  |  |  |  |  |
| Period 1 | 634 | 48.1% | 590 | 93.1% |  |  |  |  |  |  |  |  |  |  |
| Period 2 | 201 | 15.3% | 190 | 94.5% | 1.59 [0.56 - 4.47] | 0.385 | 1.50 [0.51 - 4.38] | 0.462 | 1.48 [0.50 - 4.41] | 0.481 | 1.49 [0.51 - 4.36] | 0.465 | 1.60 [0.56 - 4.51] | 0.379 |
| Period 3 | 483 | 36.6% | 453 | 93.8% | 0.85 [0.37 - 1.97] | 0.706 | 0.79 [0.34 - 1.84] | 0.589 | 0.78 [0.33 1.86] | 0.580 | 0.78 [0.34 - 1.83] | 0.574 | 0.85 [0.35 - 2.05] | 0.723 |
| **Abx use in animals** |  |  |  |  |  |  |  |  |  |  |  |  |  |  |
| No animals | 528 | 40.1% | 506 | 95.8% |  |  |  |  |  |  |  |  |  |  |
| Animals and antibiotic | 546 | 41.4% | 498 | 91.2% | 0.67 [0.27 - 1.62] | 0.372 | 0.70 [0.30 - 1.66] | 0.423 | 0.71 [0.29 - 1.72] | 0.449 | 0.78 [0.33 - 1.84] | 0.573 |  |  |
| Animals and no antibiotic | 244 | 18.5% | 229 | 93.9% | 1.53 [0.51 - 4.56] | 0.451 | 1.53 [0.51 - 4.56] | 0.451 | 1.75 [0.53 - 5.79] | 0.357 | 1.48 [0.43 - 5.14] | 0.535 |  |  |
| **Owning animals** |  |  |  |  |  |  |  |  |  |  |  |  |  |  |
| No | 528 | 40.1% | 506 | 95.8% |  |  |  |  |  |  |  |  |  |  |
| Yes | 790 | 59.9% | 727 | 92.0% | 0.79 [0.33 - 1.87] | 0.596 | 0.86 [0.37 - 2.00] | 0.729 | 0.87 [0.36 - 2.08] | 0.758 | 0.87 [0.37 - 2.05] | 0.757 | 0.90 [0.39 - 2.05] | 0.794 |
| **Owning livestock** |  |  |  |  |  |  |  |  |  |  |  |  |  |  |
| No | 617 | 46.8% | 591 | 95.8% |  |  |  |  |  |  |  |  |  |  |
| Yes | 701 | 53.2% | 642 | 91.6% | 0.90 [0.40 - 2.03] | 0.793 | 0.91 [0.41 - 2.01] | 0.818 | 0.92 [0.42 - 2.03] | 0.838 | 0.92 [0.41 - 2.06] | 0.847 | 1.02 [0.49 - 2.14] | 0.948 |
| **Owning dogs** |  |  |  |  |  |  |  |  |  |  |  |  |  |  |
| No | 797 | 60.5% | 749 | 94.0% |  |  |  |  |  |  |  |  |  |  |
| Yes | 521 | 39.5% | 485 | 93.1% | 1.03 [0.46 - 2.32] | 0.943 | 1.10 [0.50 - 2.43] | 0.806 | 1.11 [0.50 - 2.50] | 0.795 | 1.11 [0.50 - 2.46] | 0.790 | 1.08 [0.49 - 2.40] | 0.852 |
| **Owning pigs** |  |  |  |  |  |  |  |  |  |  |  |  |  |  |
| No | 1078 | 81.8% | 1022 | 94.8% |  |  |  |  |  |  |  |  |  |  |
| Yes | 240 | 18.2% | 211 | 87.9% | 0.85 [0.41 - 1.76] | 0.668 | 0.83 [0.39 - 1.74] | 0.616 | 0.83 [0.40 - 1.75] | 0.632 | 0.84 [0.40 - 1.78] | 0.648 | 0.98 [0.49 - 1.96] | 0.947 |
| **Owning chickens** |  |  |  |  |  |  |  |  |  |  |  |  |  |  |
| No | 679 | 51.5% | 647 | 95.3% |  |  |  |  |  |  |  |  |  |  |
| Yes | 639 | 48.5% | 586 | 91.7% | 0.85 [0.38 - 1.88] | 0.688 | 0.90 [0.41 - 1.97] | 0.798 | 0.91 [0.42 - 1.99] | 0.816 | 0.92 [0.41 - 2.03] | 0.828 | 1.03 [0.50 - 2.14] | 0.930 |
| **Owning ducks** |  |  |  |  |  |  |  |  |  |  |  |  |  |  |
| No | 1201 | 91.1% | 1130 | 94.1% |  |  |  |  |  |  |  |  |  |  |
| Yes | 117 | 8.9% | 103 | 88.0% | 0.90 [0.36 - 2.27] | 0.831 | 0.76 [0.29 - 1.99] | 0.572 | 0.77 [0.29 - 2.02] | 0.590 | 0.76 [0.29 - 2.01] | 0.586 | 0.63 [0.27 - 1.45] | 0.276 |
| **Owning fish** |  |  |  |  |  |  |  |  |  |  |  |  |  |  |
| No | 1283 | 97.3% | 1199 | 93.5% |  |  |  |  |  |  |  |  |  |  |
| Yes | 35 | 2.7% | 34 | 97.1% | 6.94 [0.77 - 62.97] | 0.086 | 9.03 [0.87 - 93.76] | 0.066 | 9.28 [0.89 - 96.66] | 0.064 | 9.20 [0.88 - 96.24] | 0.065 | 6.70 [0.62 - 72.47] | 0.118 |
| **Livestock owned 2** |  |  |  |  |  |  |  |  |  |  |  |  |  |  |
| No livestock | 617 | 46.8% | 591 | 95.8% |  |  |  |  |  |  |  |  |  |  |
| One species | 411 | 31.2% | 385 | 93.7% | 0.87 [0.34 - 2.20] | 0.769 | 0.90 [0.36 - 2.24] | 0.825 | 0.91 [0.37 - 2.26] | 0.838 | 0.91 [0.36 - 2.28] | 0.844 | 1.01 [0.42 - 2.43] | 0.985 |
| 2 or more species | 290 | 22.0% | 257 | 88.6% | 0.93 [0.39 - 2.25] | 0.877 | 0.92 [0.39 - 2.18] | 0.858 | 0.94 [0.40 - 2.20] | 0.881 | 0.94 [0.40 - 2.24] | 0.893 | 1.05 [0.48 - 2.29] | 0.906 |
| **Chicken consumption** |  |  |  |  |  |  |  |  |  |  |  |  |  |  |
| Less than once per week | 802 | 60.8% | 741 | 92.4% |  |  |  |  |  |  |  |  |  |  |
| Once per week | 167 | 12.7% | 152 | 91.0% | 0.65 [0.19 - 2.23] | 0.494 | 0.62 [0.19 - 2.00] | 0.422 | 0.62 [0.19 - 2.00] | 0.422 | 0.61 [0.19 - 1.98] | 0.414 | 0.55 [0.16 - 1.84] | 0.333 |
| More than once per week | 349 | 26.5% | 340 | 97.4% | 2.60 [0.95 - 7.11] | 0.064 | 2.74 [0.95 - 7.90] | 0.063 | 2.75 [0.96 - 7.92] | 0.061 | 2.73 [0.95 - 7.84] | 0.064 | 2.18 [0.74 - 6.45] | 0.159 |
| **Pork consumption** |  |  |  |  |  |  |  |  |  |  |  |  |  |  |
| Less than once per week | 144 | 10.9% | 134 | 93.1% |  |  |  |  |  |  |  |  |  |  |
| Once per week | 277 | 21.0% | 261 | 94.2% | 0.57 [0.18 - 1.78] | 0.336 | 0.55 [0.18 - 1.65] | 0.286 | 0.54 [0.18 - 1.62] | 0.272 | 0.54 [0.18 - 1.61] | 0.268 | 0.56 [0.20 - 1.61] | 0.286 |
| More than once per week | 897 | 68.1% | 838 | 93.4% | 0.65 [0.21 - 2.02] | 0.454 | 0.64 [0.21 - 1.91] | 0.422 | 0.63 [0.21 - 1.90] | 0.415 | 0.64 [0.21 - 1.91] | 0.419 | 0.60 [0.21 - 1.72] | 0.339 |
| **Beef consumption** |  |  |  |  |  |  |  |  |  |  |  |  |  |  |
| Less than once per week | 872 | 66.2% | 809 | 92.8% |  |  |  |  |  |  |  |  |  |  |
| Once per week | 193 | 14.6% | 180 | 93.3% | 0.92 [0.24 - 3.49] | 0.903 | 1.28 [0.36 - 4.48] | 0.703 | 1.27 [0.37 - 4.35] | 0.710 | 1.27 [0.36 - 4.42] | 0.712 | 1.15 [0.32 - 4.07] | 0.832 |
| More than once per week | 253 | 19.2% | 244 | 96.4% | 4.36 [1.53 - 12.43] | 0.006 | 6.56 [2.16 - 19.98] | 0.001 | 6.58 [2.16 - 19.99] | 0.001 | 6.50 [2.13 - 19.84] | 0.001 | 5.48 [1.66 - 18.06] | 0.006 |
| **Other type of meat** |  |  |  |  |  |  |  |  |  |  |  |  |  |  |
| Less than once per week | 1027 | 77.9% | 952 | 92.7% |  |  |  |  |  |  |  |  |  |  |
| Once a week or more | 291 | 22.1% | 281 | 96.6% | 1.61 [0.60 - 4.27] | 0.344 | 1.84 [0.72 - 4.69] | 0.202 | 1.83 [0.73 - 4.62] | 0.200 | 1.81 [0.73 - 4.62] | 0.200 | 1.50 [0.56 - 4.02] | 0.417 |
| **Shrimp consumption** |  |  |  |  |  |  |  |  |  |  |  |  |  |  |
| Less than once per week | 620 | 47.0% | 581 | 93.7% |  |  |  |  |  |  |  |  |  |  |
| Once per week | 457 | 34.7% | 422 | 92.3% | 0.58 [0.27 - 1.26] | 0.168 | 0.61 [0.28 - 1.30] | 0.201 | 0.60 [0.27 - 1.31] | 0.199 | 0.59 [0.27 - 1.28] | 0.182 | 0.69 [0.33 - 1.41] | 0.306 |
| More than once per week | 241 | 18.3% | 230 | 95.4% | 0.78 [0.19 - 3.17] | 0.773 | 0.78 [0.19 - 3.26] | 0.731 | 0.78 [0.19 - 3.28] | 0.735 | 0.77 [0.18 - 3.20] | 0.716 | 0.67 [0.16 - 2.88] | 0.594 |
| **Seafood consumption** |  |  |  |  |  |  |  |  |  |  |  |  |  |  |
| Less than once per week | 655 | 49.7% | 617 | 94.2% |  |  |  |  |  |  |  |  |  |  |
| Once per week | 345 | 26.2% | 307 | 89.0% | 0.66 [0.30 - 1.47] | 0.309 | 0.72 [0.31 - 1.66] | 0.442 | 0.72 [0.31 - 1.66] | 0.439 | 0.72 [0.31 - 1.66] | 0.439 | 0.71 [0.31 - 1.65] | 0.432 |
| More than once per week | 318 | 24.1% | 309 | 97.2% | 2.83 [0.92 - 8.65] | 0.070 | 3.00 [0.97 - 9.23] | 0.057 | 2.98 [0.98 - 9.11] | 0.056 | 2.98 [0.98 - 9.11] | 0.056 | 2.96 [0.99 - 8.89] | 0.054 |
| **Tofu consumption** |  |  |  |  |  |  |  |  |  |  |  |  |  |  |
| Once per week and less | 313 | 23.7% | 301 | 96.2% |  |  |  |  |  |  |  |  |  |  |
| More than once per week | 1005 | 76.3% | 932 | 92.7% | 0.58 [0.23 - 1.39] | 0.222 | 0.60 [0.24 - 1.50] | 0.277 | 0.60 [0.24 - 1.51] | 0.284 | 0.61 [0.24 - 1.52] | 0.291 | 0.69 [0.27 - 1.74] | 0.431 |
| **Dairy consumption** |  |  |  |  |  |  |  |  |  |  |  |  |  |  |
| Not every day | 491 | 37.3% | 464 | 94.5% |  |  |  |  |  |  |  |  |  |  |
| Everyday | 827 | 62.7% | 769 | 93.0% | 0.66 [0.30 - 1.43] | 0.291 | 0.70 [0.32 - 1.52] | 0.373 | 0.70 [0.32 - 1.52] | 0.371 | 0.70 [0.32 - 1.51] | 0.362 | 0.56 [0.29 - 1.09] | 0.092 |
| **Fermented food** |  |  |  |  |  |  |  |  |  |  |  |  |  |  |
| Once per week and less | 886 | 67.2% | 834 | 94.1% |  |  |  |  |  |  |  |  |  |  |
| More than once per week | 432 | 32.8% | 399 | 92.4% | 1.01 [0.49 - 2.09] | 0.972 | 0.96 [0.49 - 1.89] | 0.901 | 0.95 [0.48 - 1.89] | 0.889 | 0.94 [0.47 - 1.88] | 0.858 | 1.12 [0.58 - 2.15] | 0.741 |
| **Indoor smoking** |  |  |  |  |  |  |  |  |  |  |  |  |  |  |
| No | 896 | 68.0% | 841 | 93.9% |  |  |  |  |  |  |  |  |  |  |
| Yes | 422 | 32.0% | 392 | 92.9% | 1.00 [0.46 - 2.19] | 0.993 | 0.81 [0.38 - 1.74] | 0.589 | 0.80 [0.36 - 1.75] | 0.570 | 0.78 [0.35 - 1.73] | 0.540 | 0.93 [0.46 - 1.86] | 0.828 |
| **Flush toilet** |  |  |  |  |  |  |  |  |  |  |  |  |  |  |
| Flush toilet | 1238 | 93.9% | 1154 | 93.2% |  |  |  |  |  |  |  |  |  |  |
| Others / unknown | 80 | 6.1% | 79 | 98.8% | 12.35 [1.56 - 97.85] | 0.018 | 6.50 [0.78 - 54.16] | 0.084 | 6.55 [0.79 - 54.37] | 0.083 | 6.31 [0.76 - 52.37] | 0.089 |  |  |
| **Septic tank** |  |  |  |  |  |  |  |  |  |  |  |  |  |  |
| Septic tank | 1228 | 93.2% | 1144 | 93.2% |  |  |  |  |  |  |  |  |  |  |
| Others / unknown | 90 | 6.8% | 89 | 98.9% | 14.94 [1.91 - 117.12] | 0.011 | 7.94 [0.96 - 65.71] | 0.056 | 7.91 [0.96 - 65.25] | 0.056 | 7.76 [0.94 - 64.04] | 0.058 |  |  |
| **Drinking water** |  |  |  |  |  |  |  |  |  |  |  |  |  |  |
| Improved source | 789 | 59.9% | 751 | 95.2% |  |  |  |  |  |  |  |  |  |  |
| Rainwater only | 529 | 40.1% | 482 | 91.1% | 0.59 [0.26 - 1.33] | 0.201 | 0.52 [0.24 - 1.15] | 0.107 | 0.51 [0.23 - 1.16] | 0.109 | 0.52 [0.24 - 1.14] | 0.103 |  |  |
| **Drinking water 2** |  |  |  |  |  |  |  |  |  |  |  |  |  |  |
| Piped supply | 768 | 58.3% | 730 | 95.1% |  |  |  |  |  |  |  |  |  |  |
| Improved source | 550 | 41.7% | 503 | 91.5% | 0.64 [0.28 - 1.46] | 0.291 | 0.55 [0.25 - 1.20] | 0.133 | 0.53 [0.24 - 1.21] | 0.134 | 0.54 [0.25 - 1.19] | 0.128 |  |  |
| **Water treatment** |  |  |  |  |  |  |  |  |  |  |  |  |  |  |
| Boiling only | 1121 | 85.1% | 1042 | 93.0% |  |  |  |  |  |  |  |  |  |  |
| Other treatments | 197 | 14.9% | 191 | 97.0% | 2.77 [0.75 - 10.18] | 0.127 | 3.41 [0.83 - 14.02] | 0.090 | 3.40 [0.83 - 13.97] | 0.091 | 3.35 [0.81 - 13.81] | 0.095 |  |  |
| **WASH combined ternary** |  |  |  |  |  |  |  |  |  |  |  |  |  |  |
| Better condition | 514 | 39.0% | 467 | 90.9% |  |  |  |  |  |  |  |  |  |  |
| Middle condition | 557 | 42.3% | 525 | 94.3% | 1.51 [0.63 - 3.67] | 0.358 | 1.78 [0.77 - 4.12] | 0.180 | 1.83 [0.76 - 4.44] | 0.181 | 1.80 [0.77 - 4.19] | 0.175 |  |  |
| Worse condition | 247 | 18.7% | 241 | 97.6% | 3.96 [1.07 - 14.61] | 0.040 | 4.35 [1.07 - 17.43] | 0.039 | 4.40 [1.10 - 17.56] | 0.037 | 4.29 [1.07 - 17.15] | 0.040 |  |  |
| **WASH combined binary** |  |  |  |  |  |  |  |  |  |  |  |  |  |  |
| Better condition | 514 | 39.0% | 467 | 90.9% |  |  |  |  |  |  |  |  |  |  |
| Middle and worse condition | 804 | 61.0% | 766 | 95.3% | 1.83 [0.81 - 4.14] | 0.147 | 2.11 [0.97 - 4.62] | 0.062 | 2.17 [0.96 - 4.91] | 0.064 | 2.12 [0.97 - 4.64] | 0.061 |  |  |
| **Washing hands** |  |  |  |  |  |  |  |  |  |  |  |  |  |  |
| Rarely (< 1) | 239 | 18.1% | 229 | 95.8% |  |  |  |  |  |  |  |  |  |  |
| Sometimes (> 1) | 450 | 34.1% | 409 | 90.9% | 0.74 [0.31 - 1.77] | 0.500 | 0.92 ]0.38 - 2.21] | 0.848 | 0.89 [0.36 - 2.17] | 0.796 | 0.89 [0.37 - 2.15] | 0.789 | 0.99 [0.40 - 2.24] | 0.912 |
| Most of the times (> 1.5) | 629 | 47.7% | 595 | 94.6% | 0.79 [0.29 - 2.17] | 0.654 | 1.29 [0.50 - 3.35] | 0.597 | 1.30 [0.50 - 3.41] | 0.593 | 1.30 [0.40 - 3.39] | 0.586 | 0.99 [0.40 - 2.41] | 0.975 |
| **Washing hands 2** |  |  |  |  |  |  |  |  |  |  |  |  |  |  |
| Not often | 689 | 52.3% | 638 | 92.6% |  |  |  |  |  |  |  |  |  |  |
| Often | 629 | 47.7% | 595 | 94.6% | 0.96 [0.42 - 2.21] | 0.926 | 1.37 [0.62 - 3.07] | 0.439 | 1.41 [0.60 - 3.30] | 0.430 | 1.42 [0.62 - 3.25] | 0.412 | 1.02 [0.49 - 2.11] | 0.960 |
| **After toilet** |  |  |  |  |  |  |  |  |  |  |  |  |  |  |
| Not often | 518 | 39.3% | 480 | 92.7% |  |  |  |  |  |  |  |  |  |  |
| Often | 800 | 60.7% | 753 | 94.1% | 1.10 [0.52 - 2.38] | 0.785 | 1.56 [0.73 - 3.33] | 0.250 | 1.55 [0.73 - 3.29] | 0.251 | 1.57 [0.74 - 3.37] | 0.244 | 1.23 [0.62 - 2.45] | 0.549 |
| **Before cooking** |  |  |  |  |  |  |  |  |  |  |  |  |  |  |
| Not often | 732 | 55.5% | 676 | 92.3% |  |  |  |  |  |  |  |  |  |  |
| Often | 586 | 44.5% | 557 | 95.1% | 1.42 [0.58 - 3.43] | 0.441 | 1.94 [0.76 - 4.93] | 0.167 | 1.94 [0.76 - 4.94] | 0.164 | 1.93 [0.76 - 4.89] | 0.167 | 1.51 [0.54 - 4.24] | 0.431 |
| **Before eating** |  |  |  |  |  |  |  |  |  |  |  |  |  |  |
| Not often | 732 | 55.5% | 675 | 92.2% |  |  |  |  |  |  |  |  |  |  |
| Often | 586 | 44.5% | 558 | 95.2% | 0.93 [0.39 - 2.19] | 0.865 | 1.31 [0.56 - 3.05] | 0.539 | 1.33 [0.55 - 3.23] | 0.527 | 1.32 [0.56 - 3.13] | 0.522 | 0.89 [0.41 - 1.92] | 0.758 |
| **After eating** |  |  |  |  |  |  |  |  |  |  |  |  |  |  |
| Not often | 695 | 52.7% | 635 | 91.4% |  |  |  |  |  |  |  |  |  |  |
| Often | 623 | 47.3% | 598 | 96.0% | 1.73 [0.69 - 4.32] | 0.242 | 2.27 [0.88 - 5.90] | 0.093 | 2.32 [0.89 - 6.05] | 0.087 | 2.32 [0.88 - 6.10] | 0.090 | 1.85 [0.68 - 5.06] | 0.233 |
| **After cleaning baby** |  |  |  |  |  |  |  |  |  |  |  |  |  |  |
| Not often | 271 | 20.6% | 255 | 94.1% |  |  |  |  |  |  |  |  |  |  |
| Often | 903 | 68.5% | 846 | 93.7% | 0.86 [0.38 - 1.94] | 0.718 | 1.38 [0.60 - 3.16] | 0.450 | 1.38 [0.59 - 3.22] | 0.451 | 1.39 [0.60 - 3.22] | 0.443 | 1.25 [0.54 - 2.90] | 0.611 |
| No baby / unknown | 144 | 10.9% | 132 | 91.7% | 0.57 [0.20 - 1.67] | 0.309 | 0.73 [0.27 - 1.95] | 0.531 | 0.74 [0.28 - 1.98] | 0.547 | 0.74 [0.28 - 1.97] | 0.545 | 0.71 [0.26 - 1.93] | 0.501 |
| **Before feeding baby** |  |  |  |  |  |  |  |  |  |  |  |  |  |  |
| Not often | 480 | 36.4% | 451 | 94.0% |  |  |  |  |  |  |  |  |  |  |
| Often | 685 | 52.0% | 641 | 93.6% | 1.39 [0.65 - 2.98] | 0.398 | 2.00 [0.83 - 4.82] | 0.121 | 2.04 [0.84 - 4.93] | 0.114 | 2.06 [0.83 - 5.10] | 0.121 | 1.73 [0.61 - 4.89] | 0.302 |
| No baby / unknown | 153 | 11.6% | 141 | 92.2% | 0.70 [0.26 - 1.91] | 0.487 | 0.81 [0.31 - 2.11] | 0.664 | 0.83 [0.32 - 2.13] | 0.693 | 0.83 [0.32 - 2.16] | 0.698 | 0.78 [0.28 - 2.18] | 0.643 |
| **After handling animals** |  |  |  |  |  |  |  |  |  |  |  |  |  |  |
| Not often | 198 | 15.0% | 187 | 94.4% |  |  |  |  |  |  |  |  |  |  |
| Often | 592 | 44.9% | 540 | 91.2% | 0.53 [0.18 - 1.58] | 0.256 | 0.62 [0.22 - 1.78] | 0.377 | 0.62 [0.21 - 1.79] | 0.377 | 0.62 [0.22 - 1.80] | 0.385 | 0.69 [0.25 - 1.89] | 0.472 |
| Not owning animals | 528 | 40.1% | 506 | 95.8% | 0.73 [0.22 - 2.43] | 0.609 | 0.75 [0.24 - 2.33] | 0.621 | 0.76 [0.24 - 2.37] | 0.632 | 0.75 [0.24 - 2.33] | 0.621 | 0.80 [0.27 - 2.40] | 0.696 |
| **After sneezing** |  |  |  |  |  |  |  |  |  |  |  |  |  |  |
| Not often | 1198 | 90.9% | 1126 | 94.0% |  |  |  |  |  |  |  |  |  |  |
| Often | 120 | 9.1% | 107 | 89.2% | 0.65 [0.27 - 1.58] | 0.346 | 0.62 [0.30 - 1.29] | 0.203 | 0.62 [0.30 - 1.30] | 0.210 | 0.62 [0.30 - 1.31] | 0.212 | 0.76 [0.38 - 1.54] | 0.452 |
| **Distance to healthcare** |  |  |  |  |  |  |  |  |  |  |  |  |  |  |
| Equal or less than 10 minutes | 984 | 74.7% | 920 | 93.5% |  |  |  |  |  |  |  |  |  |  |
| More than 10 minutes | 334 | 25.3% | 313 | 93.7% | 1.51 [0.68 - 3.32] | 0.311 | 1.31 [0.62 - 2.80] | 0.479 | 1.31 [0.62 - 2.79] | 0.484 | 1.30 [0.61 - 2.78] | 0.497 | 1.54 [0.78 - 3.05] | 0.218 |
| **Household AMU 4 weeks** |  |  |  |  |  |  |  |  |  |  |  |  |  |  |
| No | 778 | 59.0% | 723 | 92.9% |  |  |  |  |  |  |  |  |  |  |
| Yes | 540 | 41.0% | 510 | 94.4% | 1.31 [0.58 - 2.98] | 0.517 | 1.24 [0.56 - 2.73] | 0.600 | 1.22 [0.53 - 2.81] | 0.646 | 1.19 [0.52 - 2.74] | 0.681 | 1.39 [0.58 - 3.31] | 0.459 |
| **Overcrowding (2 person per room)** |  |  |  |  |  |  |  |  |  |  |  |  |  |  |
| No | 965 | 73.2% | 909 | 94.2% |  |  |  |  |  |  |  |  |  |  |
| Yes | 343 | 26.0% | 314 | 91.6% | 1.30 [0.62 – 2.73] | 0.480 | 0.99 [0.46 – 2.13] | 0.985 | 1.00 [0.46 – 2.14] | 0.991 | 1.00 [0.46 – 2.14] | 0.994 | 0.97 [0.46 – 2.02] | 0.927 |
| Unknown | 10 | 0.8% | 10 | 100% |  |  |  |  |  |  |  |  |  |  |

**Supplementary file 6**: Complete list of stratified C3GRE analyses (unweighted)

Model 1 strata 1 – Unweighted crude model of O5 individuals (OR)

Model 2 strata 1 – Unweighted crude model of O5 individuals + wealth (aOR)

Model 1 strata 2 – Unweighted crude model of U5 individuals (OR)

Model 2 strata 2 – Unweighted crude model of U5 individuals + wealth (aOR)

|  | STRATA 1 (PARTICIPANTS OVER 5) | | | | | | | | STRATA 2 (PARTICIPANTS UNDER 5) | | | | | | | |
| --- | --- | --- | --- | --- | --- | --- | --- | --- | --- | --- | --- | --- | --- | --- | --- | --- |
|  | Total | | CRE carrier | | MODELS STRATA 1 | | | | Total | | CRE carrier | | MODEL STRATA 2 | | | |
|  | N | % | N | % | OR 95% CI | p-value | aOR 95% CI | p-value | N | % | N | % | OR 95% CI | p-value | aOR 95% CI | p-value |
| **All** | 1107 | 84.0% | 1046 | 94.5% |  |  |  |  | 211 | 16.0% |  | 0.0% |  |  |  |  |
| **Economic status** |  |  |  |  |  |  |  |  |  |  |  |  |  |  |  |  |
| Low income | 386 | 34.9% | 371 | 96.1% |  |  |  |  | 67 | 31.8% | 63 | 94.0% |  |  |  |  |
| Middle class | 355 | 32.1% | 331 | 93.2% | 0.56 [0.27 - 1.14] | 0.110 |  |  | 68 | 32.2% | 58 | 85.3% | 0.37 [0.11 - 1.23] | 0.107 |  |  |
| Wealthy | 366 | 33.1% | 344 | 94.0% | 0.63 [0.30 - 1.35] | 0.237 |  |  | 76 | 36.0% | 66 | 86.8% | 0.42 [0.12 - 1.49] | 0.181 |  |  |
| **Illness** |  |  |  |  |  |  |  |  |  |  |  |  |  |  |  |  |
| No and unknown | 1040 | 93.9% | 983 | 94.5% |  |  |  |  | 175 | 82.9% | 152 | 86.9% |  |  |  |  |
| Yes | 67 | 6.1% | 63 | 94.0% | 0.91 [0.32 - 2.57] | 0.864 | 0.92 [0.34 - 2.55] | 0.880 | 36 | 17.1% | 35 | 97.2% | 5.30 [0.72 - 38.91] | 0.103 | 5.32 [0.67 - 42.01] | 0.115 |
| **Antibiotic use 4 weeks** |  |  |  |  |  |  |  |  |  |  |  |  |  |  |  |  |
| No and unknown | 1003 | 90.6% | 946 | 94.3% |  |  |  |  | 147 | 69.7% | 130 | 88.4% |  |  |  |  |
| Yes | 104 | 9.4% | 100 | 96.2% | 1.51 [0.53 - 4.25] | 0.440 | 1.50 [0.54 - 4.21] | 0.441 | 64 | 30.3% | 57 | 89.1% | 1.06 [0.45 - 2.50] | 0.886 | 1.11 [0.46 - 2.68] | 0.813 |
| **Seasonality** |  |  |  |  |  |  |  |  |  |  |  |  |  |  |  |  |
| Spring and summer 1 | 805 | 72.7% | 752 | 93.4% |  |  |  |  | 157 | 74.4% | 137 | 87.3% |  |  |  |  |
| Autumn and winter 2 | 302 | 27.3% | 294 | 97.4% | 2.59 [1.01 - 6.67] | 0.050 | 2.68 [1.01 - 7.17] | 0.050 | 54 | 25.6% | 50 | 92.6% | 1.82 [0.60 - 5.57] | 0.292 | 2.13 [0.70 - 6.50] | 0.188 |
| **Period of sampling** |  |  |  |  |  |  |  |  |  |  |  |  |  |  |  |  |
| Period 1 | 524 | 47.3% | 490 | 93.5% |  |  |  |  | 110 | 52.1% | 100 | 90.9% |  |  |  |  |
| Period 2 | 173 | 15.6% | 165 | 95.4% | 1.43 [0.55 - 3.72] | 0.463 | 1.43 [0.55 - 3.75] | 0.469 | 28 | 13.3% | 25 | 89.3% | 0.83 [0.21 - 3.24] | 0.793 | 0.95 [0.25 - 3.57] | 0.935 |
| Period 3 | 410 | 37.0% | 391 | 95.4% | 1.43 [0.76 - 2.67] | 0.264 | 1.47 [0.77 - 2.78] | 0.240 | 73 | 34.6% | 62 | 84.9% | 0.56 [0.21 - 1.51] | 0.257 | 0.56 [0.20 - 1.54] | 0.262 |
| **Abx use in animals** |  |  |  |  |  |  |  |  |  |  |  |  |  |  |  |  |
| No animals | 441 | 39.8% | 423 | 95.9% |  |  |  |  | 87 | 41.2% | 83 | 95.4% |  |  |  |  |
| Animals and antibiotic | 460 | 41.6% | 424 | 92.2% | 0.50 [0.25 - 0.99] | 0.048 | 0.52 [0.26 - 1.00] | 0.053 | 86 | 40.8% | 74 | 86.0% | 0.30 [0.09 - 0.95] | 0.043 | 0.31 [0.10 - 0.99] | 0.051 |
| Animals and no antibiotic | 206 | 18.6% | 199 | 96.6% | 1.21 [0.44 - 3.29] | 0.710 | 1.31 [0.48 - 3.60] | 0.602 | 38 | 18.0% | 30 | 78.9% | 0.18 [0.05 - 0.72] | 0.016 | 0.20 [0.05 - 0.82] | 0.027 |
| **Owning animals** |  |  |  |  |  |  |  |  |  |  |  |  |  |  |  |  |
| No | 441 | 39.8% | 423 | 95.9% |  |  |  |  | 87 | 41.2% | 83 | 95.4% |  |  |  |  |
| Yes | 666 | 60.2% | 623 | 93.5% | 0.62 [0.32 - 1.20] | 0.157 | 0.64 [0.33 - 1.24] | 0.190 | 124 | 58.8% | 104 | 83.9% | 0.25 [0.08 - 0.77] | 0.017 | 0.27 [0.09 - 0.84] | 0.025 |
| **Owning livestock** |  |  |  |  |  |  |  |  |  |  |  |  |  |  |  |  |
| No | 515 | 46.5% | 494 | 95.9% |  |  |  |  | 102 | 48.3% | 97 | 95.1% |  |  |  |  |
| Yes | 592 | 53.5% | 552 | 93.2% | 0.59 [0.31 - 1.11] | 0.100 | 0.61 [0.33 - 1.13] | 0.115 | 109 | 51.7% | 90 | 82.6% | 0.24 [0.09 - 0.69] | 0.009 | 0.26 [0.09 - 0.74] | 0.012 |
| **Owning dogs** |  |  |  |  |  |  |  |  |  |  |  |  |  |  |  |  |
| No | 667 | 60.3% | 631 | 94.6% |  |  |  |  | 130 | 61.6% | 117 | 90.0% |  |  |  |  |
| Yes | 440 | 39.7% | 415 | 94.3% | 0.95 [0.53 - 1.69] | 0.854 | 0.95 [0.54 - 1.70] | 0.869 | 81 | 38.4% | 70 | 86.4% | 0.71 [0.28 - 1.76] | 0.458 | 0.69 [0.28 - 1.71] | 0.427 |
| **Owning pigs** |  |  |  |  |  |  |  |  |  |  |  |  |  |  |  |  |
| No | 902 | 81.5% | 861 | 95.5% |  |  |  |  | 176 | 83.4% | 161 | 91.5% |  |  |  |  |
| Yes | 205 | 18.5% | 185 | 90.2% | 0.44 [0.24 - 0.80] | 0.007 | 0.45 [0.25 - 0.79] | 0.006 | 35 | 16.6% | 26 | 74.3% | 0.27 [0.10 - 0.71] | 0.009 | 0.29 [0.11 - 0.76] | 0.013 |
| **Owning chickens** |  |  |  |  |  |  |  |  |  |  |  |  |  |  |  |  |
| No | 566 | 51.1% | 539 | 95.2% |  |  |  |  | 113 | 53.6% | 108 | 95.6% |  |  |  |  |
| Yes | 541 | 48.9% | 507 | 93.7% | 0.75 [0.41 - 1.35] | 0.332 | 0.79 [0.44 - 1.41] | 0.424 | 98 | 46.4% | 79 | 80.6% | 0.19 [0.07 - 0.55] | 0.002 | 0.21 [0.07 - 0.59] | 0.004 |
| **Owning ducks** |  |  |  |  |  |  |  |  |  |  |  |  |  |  |  |  |
| No | 1011 | 91.3% | 957 | 94.7% |  |  |  |  | 190 | 90.0% | 173 | 91.1% |  |  |  |  |
| Yes | 96 | 8.7% | 89 | 92.7% | 0.72 [0.33 - 1.58] | 0.409 | 0.75 [0.33 - 1.69] | 0.486 | 21 | 10.0% | 14 | 66.7% | 0.20 [0.06 - 0.67] | 0.010 | 0.20 [0.06 - 0.66] | 0.009 |
| **Owning fish** |  |  |  |  |  |  |  |  |  |  |  |  |  |  |  |  |
| No | 1079 | 97.5% | 1019 | 94.4% |  |  |  |  | 204 | 96.7% | 189 | 92.6% |  |  |  |  |
| Yes | 28 | 2.5% | 27 | 2.4% | 1.59 [0.25 - 10.17] | 0.625 | 1.55 [0.21 - 11.25] | 0.667 | 7 | 3.3% | 7 | 100.0% |  | < 0.001 |  | < 0.001 |
| **Livestock owned 2** |  |  |  |  |  |  |  |  |  |  |  |  |  |  |  |  |
| No livestock | 515 | 46.5% | 494 | 95.9% |  |  |  |  | 102 | 48.3% | 97 | 95.1% |  |  |  |  |
| One species | 345 | 31.2% | 325 | 94.2% | 0.69 [0.34 - 1.40] | 0.304 | 0.71 [0.36 - 1.43] | 0.343 | 66 | 31.3% | 60 | 90.9% | 0.52 [0.15 - 1.76] | 0.291 | 0.55 [0.16 - 1.94] | 0.357 |
| 2 or more species | 247 | 22.3% | 227 | 91.9% | 0.48 [0.23 - 0.99] | 0.049 | 0.50 [0.25 - 1.01] | 0.053 | 43 | 20.4% | 30 | 69.8% | 0.12 [0.04 - 0.37] | < 0.001 | 0.12 [0.04 - 0.39] | < 0.001 |
| **Chicken consumption** |  |  |  |  |  |  |  |  |  |  |  |  |  |  |  |  |
| Less than once per week | 680 | 61.4% | 636 | 93.5% |  |  |  |  | 122 | 57.8% | 105 | 86.1% |  |  |  |  |
| Once per week | 138 | 12.5% | 127 | 92.0% | 0.80 [0.35 - 1.80] | 0.588 | 0.79 [0.35 - 1.77] | 0.568 | 29 | 13.7% | 25 | 86.2% | 1.01 [0.31 - 3.31] | 0.984 | 0.94 [0.27 - 3.28] | 0.927 |
| More than once per week | 289 | 26.1% | 283 | 97.9% | 3.26 [1.40 - 7.59] | 0.006 | 3.45 [1.45 - 8.22] | 0.006 | 60 | 28.4% | 57 | 95.0% | 3.08 [0.85 - 11.09] | 0.088 | 3.41 [0.96 - 12.09] | 0.059 |
| **Pork consumption** |  |  |  |  |  |  |  |  |  |  |  |  |  |  |  |  |
| Less than once per week | 119 | 10.7% | 114 | 95.8% |  |  |  |  | 25 | 11.8% | 20 | 80.0% |  |  |  |  |
| Once per week | 241 | 21.8% | 226 | 93.8% | 0.66 [0.24 - 1.80] | 0.418 | 0.66 [0.24 - 1.83] | 0.427 | 36 | 17.1% | 35 | 97.2% | 8.75 [0.89 - 85.79] | 0.064 | 7.96 [0.81 - 78.73] | 0.078 |
| More than once per week | 747 | 67.5% | 706 | 94.5% | 0.76 [0.30 - 1.87] | 0.545 | 0.76 [0.30 - 1.90] | 0.554 | 150 | 71.1% | 132 | 88.0% | 1.83 [0.54 - 6.19] | 0.330 | 1.70 [0.49 - 5.88] | 0.407 |
| **Beef consumption** |  |  |  |  |  |  |  |  |  |  |  |  |  |  |  |  |
| Less than once per week | 748 | 67.6% | 702 | 93.9% |  |  |  |  | 124 | 58.8% | 107 | 86.3% |  |  |  |  |
| Once per week | 155 | 14.0% | 146 | 94.2% | 1.06 [0.47 - 2.38] | 0.882 | 1.13 [0.50 - 2.57] | 0.767 | 38 | 18.0% | 34 | 89.5% | 1.35 [0.44 - 4.17] | 0.602 | 1.39 [0.44 - 4.41] | 0.576 |
| More than once per week | 204 | 18.4% | 198 | 97.1% | 2.16 [0.82 - 5.68] | 0.119 | 2.47 [0.89 - 6.88] | 0.085 | 49 | 23.2% | 46 | 93.9% | 2.44 [0.55 - 9.03] | 0.185 | 2.92 [0.69 - 12.37] | 0.148 |
| **Other type of meat** |  |  |  |  |  |  |  |  |  |  |  |  |  |  |  |  |
| Less than once per week | 870 | 78.6% | 815 | 93.7% |  |  |  |  | 157 | 74.4% | 137 | 87.3% |  |  |  |  |
| Once a week or more | 237 | 21.4% | 231 | 97.5% | 2.60 [1.01 - 6.68] | 0.049 | 2.89 [1.15 - 7.29] | 0.025 | 54 | 25.6% | 50 | 92.6% | 1.82 [0.59 - 5.68] | 0.301 | 2.00 [0.65 - 6.15] | 0.230 |
| **Shrimp consumption** |  |  |  |  |  |  |  |  |  |  |  |  |  |  |  |  |
| Less than once per week | 526 | 47.5% | 499 | 94.9% |  |  |  |  | 94 | 44.5% | 82 | 87.2% |  |  |  |  |
| Once per week | 382 | 34.5% | 356 | 93.2% | 0.74 [0.41 - 1.35] | 0.327 | 0.78 [0.42 - 1.44] | 0.428 | 75 | 35.5% | 66 | 88.0% | 1.07 [0.41 - 2.84] | 0.887 | 1.06 [0.40 - 2.84] | 0.910 |
| More than once per week | 199 | 18.0% | 191 | 96.0% | 1.29 [0.48 - 3.47] | 0.612 | 1.36 [0.50 - 3.72] | 0.546 | 42 | 19.9% | 39 | 92.9% | 1.90 [0.48 - 7.50] | 0.360 | 2.02 [0.52 - 7.79] | 0.310 |
| **Seafood consumption** |  |  |  |  |  |  |  |  |  |  |  |  |  |  |  |  |
| Less than once per week | 549 | 49.6% | 523 | 95.3% |  |  |  |  | 106 | 50.2% | 94 | 88.7% |  |  |  |  |
| Once per week | 289 | 26.1% | 261 | 90.3% | 0.46 [0.25 - 0.85] | 0.014 | 0.47 [0.25 - 0.88] | 0.019 | 56 | 26.5% | 46 | 82.1% | 0.59 [0.22 - 1.56] | 0.286 | 0.59 [0.22 - 1.58] | 0.292 |
| More than once per week | 269 | 24.3% | 262 | 97.4% | 1.86 [0.80 - 4.31] | 0.148 | 1.88 [0.82 - 4.30] | 0.137 | 49 | 23.2% | 47 | 95.9% | 3.00 [0.63 - 14.31] | 0.170 | 2.88 [0.60 - 13.76] | 0.186 |
| **Tofu consumption** |  |  |  |  |  |  |  |  |  |  |  |  |  |  |  |  |
| Once per week and less | 262 | 23.7% | 256 | 97.7% |  |  |  |  | 51 | 24.2% | 45 | 88.2% |  |  |  |  |
| More than once per week | 845 | 76.3% | 790 | 93.5% | 0.34 [0.15 - 0.77] | 0.010 | 0.32 [0.14 - 0.74] | 0.009 | 160 | 75.8% | 142 | 88.8% | 1.05 [0.35 - 3.20] | 0.929 | 0.93 [0.31 - 2.78] | 0.891 |
| **Dairy consumption** |  |  |  |  |  |  |  |  |  |  |  |  |  |  |  |  |
| Not every day | 426 | 38.5% | 408 | 95.8% |  |  |  |  | 65 | 30.8% | 56 | 86.2% |  |  |  |  |
| Everyday | 681 | 61.5% | 638 | 93.7% | 0.65 [0.36 - 1.20] | 0.171 | 0.67 [0.36 - 1.23] | 0.194 | 146 | 69.2% | 131 | 89.7% | 1.40 [0.54 - 3.63] | 0.485 | 1.49 [0.57 - 3.91] | 0.421 |
| **Fermented food** |  |  |  |  |  |  |  |  |  |  |  |  |  |  |  |  |
| Once per week and less | 748 | 67.6% | 715 | 95.6% |  |  |  |  | 138 | 65.4% | 119 | 86.2% |  |  |  |  |
| More than once per week | 359 | 32.4% | 331 | 92.2% | 0.55 [0.31 - 0.97] | 0.039 | 0.55 [0.31 - 0.96] | 0.037 | 73 | 34.6% | 68 | 93.2% | 2.17 [0.76 - 6.20] | 0.149 | 2.11 [0.74 - 6.03] | 0.166 |
| **Indoor smoking** |  |  |  |  |  |  |  |  |  |  |  |  |  |  |  |  |
| No | 746 | 67.4% | 710 | 95.2% |  |  |  |  | 150 | 71.1% | 131 | 87.3% |  |  |  |  |
| Yes | 361 | 32.6% | 336 | 93.1% | 0.68 [0.38 - 1.22] | 0.196 | 0.67 [0.37 - 1.19] | 0.175 | 61 | 28.9% | 56 | 91.8% | 1.62 [0.57 - 4.60] | 0.363 | 1.49 [0.53 - 4.19] | 0.450 |
| **Flush toilet** |  |  |  |  |  |  |  |  |  |  |  |  |  |  |  |  |
| Flush toilet | 1042 | 94.1% | 982 | 94.2% |  |  |  |  | 196 | 92.9% | 172 | 87.8% |  |  |  |  |
| Others / unknown | 65 | 5.9% | 64 | 98.5% | 3.91 [0.57 - 26.81] | 0.166 | 2.95 [0.41 - 21.34] | 0.286 | 15 | 7.1% | 15 | 100.0% |  | < 0.001 |  | < 0.001 |
| **Septic tank** |  |  |  |  |  |  |  |  |  |  |  |  |  |  |  |  |
| Septic tank | 1033 | 93.3% | 973 | 94.2% |  |  |  |  | 195 | 92.4% | 171 | 87.7% |  |  |  |  |
| Others / unknown | 74 | 6.7% | 73 | 98.6% | 4.50 [0.65 - 31.11] | 0.128 | 3.45 [0.47 - 25.22] | 0.223 | 16 | 7.6% | 16 | 100.0% |  | < 0.001 |  | < 0.001 |
| **Drinking water** |  |  |  |  |  |  |  |  |  |  |  |  |  |  |  |  |
| Improved source | 656 | 59.3% | 631 | 96.2% |  |  |  |  | 133 | 63.0% | 120 | 90.2% |  |  |  |  |
| Rainwater | 451 | 40.7% | 415 | 92.0% | 0.46 [0.25 - 0.82] | 0.009 | 0.44 [0.25 - 0.79] | 0.007 | 78 | 37.0% | 67 | 85.9% | 0.66 [0.27 - 1.64] | 0.372 | 0.65 [0.22 - 1.90] | 0.436 |
| **Drinking water 2** |  |  |  |  |  |  |  |  |  |  |  |  |  |  |  |  |
| Piped supply | 638 | 57.6% | 613 | 96.1% |  |  |  |  | 130 | 61.6% | 117 | 90.0% |  |  |  |  |
| Improved source | 469 | 42.4% | 433 | 92.3% | 0.49 [0.27 - 0.88] | 0.018 | 0.46 [0.26 - 9.83] | 0.011 | 81 | 38.4% | 70 | 86.4% | 0.71 [0.28 - 1.76] | 0.457 | 0.68 [0.23 - 1.97] | 0.477 |
| **Water treatment** |  |  |  |  |  |  |  |  |  |  |  |  |  |  |  |  |
| Boiling only | 943 | 85.2% | 887 | 94.1% |  |  |  |  | 178 | 84.4% | 155 | 87.1% |  |  |  |  |
| Other treatments | 164 | 14.8% | 159 | 97.0% | 2.01 [0.82 - 4.94] | 0.130 | 2.11 [0.82 - 5.45] | 0.123 | 33 | 15.6% | 32 | 97.0% | 4.75 [0.61 - 37.20] | 0.140 | 5.61 [0.63 - 50.19] | 0.125 |
| **WASH combined ternary** |  |  |  |  |  |  |  |  |  |  |  |  |  |  |  |  |
| Better condition | 437 | 39.5% | 401 | 91.8% |  |  |  |  | 77 | 36.5% | 66 | 85.7% |  |  |  |  |
| Middle condition | 467 | 42.2% | 447 | 95.7% | 2.01 [1.05 - 3.83] | 0.036 | 2.11 [1.11 - 4.00] | 0.023 | 90 | 42.7% | 78 | 86.7% | 1.08 [0.42 - 2.76] | 0.867 | 1.13 [0.40 - 3.21] | 0.814 |
| Worse condition | 203 | 18.3% | 198 | 97.5% | 3.56 [1.41 - 8.96] | 0.008 | 3.51 [1.35 - 9.17] | 0.011 | 44 | 20.9% | 43 | 97.7% | 7.17 [0.87 - 58.98] | 0.069 | 7.18 [0.71 - 72.68] | 0.097 |
| **WASH combined binary** |  |  |  |  |  |  |  |  |  |  |  |  |  |  |  |  |
| Better condition | 437 | 39.5% | 401 | 91.8% |  |  |  |  | 77 | 36.5% | 66 | 85.7% |  |  |  |  |
| Middle and worse condition | 670 | 60.5% | 645 | 96.3% | 2.32 [1.29 - 4.16] | 0.005 | 2.38 [1.32 - 4.29] | 0.004 | 134 | 63.5% | 121 | 90.3% | 1.55 [0.62 - 3.86] | 0.346 | 1.55 [0.53 - 4.55] | 0.423 |
| **Washing hands** |  |  |  |  |  |  |  |  |  |  |  |  |  |  |  |  |
| Rarely (< 1) | 210 | 19.0% | 201 | 95.7% |  |  |  |  | 29 | 13.7% | 28 | 96.6% |  |  |  |  |
| Sometimes (> 1) | 381 | 34.4% | 351 | 92.1% | 0.52 [0.24 - 1.16] | 0.111 | 0.61 [0.26 - 1.42] | 0.253 | 69 | 32.7% | 58 | 84.1% | 0.19 [0.02 - 1.57] | 0.125 | 0.22 [0.03 - 1.84] | 0.164 |
| Most of the times (> 1.5) | 516 | 46.6% | 494 | 95.7% | 1.01 [0.43 - 2.37] | 0.990 | 1.34 [0.55 - 3.24] | 0.521 | 113 | 53.6% | 101 | 89.4% | 0.30 [0.04 - 2.48] | 0.266 | 0.46 [0.06 - 3.92] | 0.483 |
| **Washing hands 2** |  |  |  |  |  |  |  |  |  |  |  |  |  |  |  |  |
| Not often | 591 | 53.4% | 552 | 93.4% |  |  |  |  | 98 | 46.4% | 86 | 87.8% |  |  |  |  |
| Often | 516 | 46.6% | 494 | 95.7% | 1.59 [0.86 - 2.93] | 0.141 | 1.94 [1.05 - 3.60] | 0.035 | 113 | 53.6% | 101 | 89.4% | 1.17 [0.47 - 2.91] | 0.729 | 1.67 [0.54 - 5.15] | 0.376 |
| **After toilet** |  |  |  |  |  |  |  |  |  |  |  |  |  |  |  |  |
| Not often | 438 | 39.6% | 408 | 93.2% |  |  |  |  | 80 | 37.9% | 72 | 90.0% |  |  |  |  |
| Often | 669 | 60.4% | 638 | 95.4% | 1.51 [0.85 - 2.68] | 0.156 | 1.86 [1.03 - 3.36] | 0.040 | 131 | 62.1% | 115 | 87.8% | 0.80 [0.30 - 2.12] | 0.652 | 0.99 [0.31 - 3.20] | 0.991 |
| **Before cooking** |  |  |  |  |  |  |  |  |  |  |  |  |  |  |  |  |
| Not often | 621 | 56.1% | 577 | 92.9% |  |  |  |  | 111 | 52.6% | 99 | 89.2% |  |  |  |  |
| Often | 486 | 43.9% | 569 | 117.1% | 2.10 [1.10 - 4.04] | 0.026 | 2.63 [1.36 - 5.08] | 0.004 | 100 | 47.4% | 88 | 88.0% | 0.89 [0.36 - 2.20] | 0.800 | 1.05 [0.36 - 3.18] | 0.925 |
| **Before eating** |  |  |  |  |  |  |  |  |  |  |  |  |  |  |  |  |
| Not often | 627 | 56.6% | 583 | 93.0% |  |  |  |  | 105 | 49.8% | 92 | 87.6% |  |  |  |  |
| Often | 480 | 43.4% | 463 | 96.5% | 2.06 [1.07 - 3.95] | 0.031 | 2.58 [1.34 - 4.97] | 0.005 | 106 | 50.2% | 95 | 89.6% | 1.22 [0.49 - 3.04] | 0.669 | 1.54 [0.53 - 4.46] | 0.423 |
| **After eating** |  |  |  |  |  |  |  |  |  |  |  |  |  |  |  |  |
| Not often | 591 | 53.4% | 545 | 92.2% |  |  |  |  | 104 | 49.3% | 90 | 86.5% |  |  |  |  |
| Often | 516 | 46.6% | 501 | 97.1% | 2.82 [1.44 - 5.51] | 0.003 | 3.66 [1.84 - 7.29] | < 0.001 | 107 | 50.7% | 97 | 90.7% | 1.51 [0.60 - 3.80] | 0.384 | 2.18 [0.66 - 7.21] | 0.203 |
| **After cleaning baby** |  |  |  |  |  |  |  |  |  |  |  |  |  |  |  |  |
| Not often | 236 | 21.3% | 223 | 94.5% |  |  |  |  | 35 | 16.6% | 32 | 91.4% |  |  |  |  |
| Often | 729 | 65.9% | 692 | 94.9% | 1.12 [0.57 - 2.21] | 0.744 | 1.30 [0.62 - 2.73] | 0.482 | 175 | 82.9% | 154 | 88.0% | 0.69 [0.19 - 2.51] | 0.571 | 0.85 [0.23 - 3.17] | 0.808 |
| No baby / unknown | 143 | 12.9% | 131 | 91.6% | 0.64 [0.25 - 1.65] | 0.353 | 0.67 [0.27 - 1.63] | 0.376 | 1 | 0.5% | 1 | 100.0% |  | < 0.001 |  | < 0.001 |
| **Before feeding baby** |  |  |  |  |  |  |  |  |  |  |  |  |  |  |  |  |
| Not often | 405 | 36.6% | 384 | 94.8% |  |  |  |  | 75 | 35.5% | 67 | 89.3% |  |  |  |  |
| Often | 550 | 49.7% | 522 | 94.9% | 1.02 [0.56 - 1.87] | 0.950 | 1.11 [0.56 - 2.18] | 0.769 | 135 | 64.0% | 119 | 88.1% | 0.14 [0.02 - 1.14] | 0.068 | 0.15 [0.02 - 1.23] | 0.079 |
| No baby / unknown | 152 | 13.7% | 140 | 92.1% | 0.64 [0.27 - 1.53] | 0.314 | 0.63 [0.27 - 1.47] | 0.291 | 1 | 0.5% | 1 | 100.0% | 0.74 [0.08 - 6.96] | 0.794 | 0.71 [0.07 - 7.27] | 0.774 |
| **After handling animals** |  |  |  |  |  |  |  |  |  |  |  |  |  |  |  |  |
| Not often | 169 | 15.3% | 159 | 94.1% |  |  |  |  | 29 | 13.7% | 28 | 96.6% |  |  |  |  |
| Often | 497 | 44.9% | 464 | 93.4% | 0.88 [0.42 - 1.86] | 0.747 | 0.92 [0.44 - 1.92] | 0.822 | 95 | 45.0% | 76 | 80.0% | 0.14 [0.02 - 1.14] | 0.068 | 0.15 [0.02 - 1.23] | 0.079 |
| Not owning animals | 441 | 39.8% | 423 | 95.9% | 1.48 [0.62 - 3.54] | 0.381 | 1.46 [0.62 - 3.43] | 0.390 | 87 | 41.2% | 83 | 95.4% | 0.74 [0.08 - 6.96] | 0.794 | 0.71 [0.07 - 7.27] | 0.774 |
| **After sneezing** |  |  |  |  |  |  |  |  |  |  |  |  |  |  |  |  |
| Not often | 1003 | 90.6% | 952 | 94.9% |  |  |  |  | 195 | 92.4% | 174 | 89.2% |  |  |  |  |
| Often | 104 | 9.4% | 94 | 90.4% | 0.50 [0.24 - 1.04] | 0.065 | 0.52 [0.25 - 1.07] | 0.076 | 16 | 7.6% | 13 | 81.3% | 0.52 [0.14 - 1.90] | 0.327 | 0.55 [0.16 - 1.91] | 0.349 |
| **Household AMU 4 weeks** |  |  |  |  |  |  |  |  |  |  |  |  |  |  |  |  |
| No | 663 | 59.9% | 622 | 93.8% |  |  |  |  | 115 | 54.5% | 101 | 87.8% |  |  |  |  |
| Yes | 444 | 40.1% | 424 | 95.5% | 1.40 [0.76 - 2.56] | 0.279 | 1.41 [0.77 - 2.61] | 0.269 | 96 | 45.5% | 86 | 89.6% | 1.19 [0.47 - 3.06] | 0.715 | 1.26 [0.49 - 3.20] | 0.634 |

**Supplementary file 7**: Complete list of C3GRE analyses of birth & immunisation factors (unweighted)

Model 1 – Weighted crude model (OR)

Model 2 – Weighted crude model + wealth + age in years (aOR)

Model 3 – Weighted crude model + wealth + age in years + AMU (mediating effect)

|  | Total | | C3GRE carrier | | MODEL 1 | | MODEL 2 | | MODEL 3 | |  |
| --- | --- | --- | --- | --- | --- | --- | --- | --- | --- | --- | --- |
|  | N | % | N | % | OR  95% CI | p-value | OR  95% CI | p-value | OR  95% CI | p-value | |
| **Age** |  |  |  |  |  |  |  |  |  |  | |
| < 1 year | 13 | 6.4% | 10 | 76.9% |  |  |  |  |  |  | |
| 1 year | 49 | 24.1% | 45 | 91.8% | 3.37 [0.64 - 17.74] | 0.153 |  |  |  |  | |
| 2 years | 48 | 23.6% | 42 | 87.5% | 2.10 [0.50 - 8.77] | 0.310 |  |  |  |  | |
| 3 years | 50 | 24.6% | 46 | 92.0% | 3.45 [0.66 - 17.98] | 0.143 |  |  |  |  | |
| 4 years | 43 | 21.2% | 39 | 90.7% | 2.92 [0.57 - 15.04] | 0.201 |  |  |  |  | |
| **Economic status** |  |  |  |  |  |  |  |  |  |  | |
| Low income | 66 | 32.5% | 62 | 93.9% |  |  |  |  |  |  | |
| Middle class | 63 | 31.0% | 55 | 87.3% | 0.44 [0.13 - 1.56] | 0.208 |  |  |  |  | |
| Wealthy | 74 | 36.5% | 65 | 87.8% | 0.47 [0.13 - 1.65] | 0.238 |  |  |  |  | |
| **Illness** |  |  |  |  |  |  |  |  |  |  | |
| No and unknown | 167 | 82.3% | 147 | 88.0% |  |  |  |  |  |  | |
| Yes | 36 | 17.7% | 35 | 97.2% | 4.76 [0.64 - 35.21] | 0.128 | 4.34 [0.55 - 34.10] | 0.165 | 6.19 [0.70 - 54.83] | 0.104 | |
| **Antibiotic use 4 weeks** |  |  |  |  |  |  |  |  |  |  | |
| No and unknown | 139 | 68.5% | 125 | 89.9% |  |  |  |  |  |  | |
| Yes | 64 | 31.5% | 57 | 89.1% | 0.91 [0.36 - 2.28] | 0.844 | 0.94 [0.33 - 2.73] | 0.915 |  |  | |
| **Seasonality** |  |  |  |  |  |  |  |  |  |  | |
| Spring and summer 1 | 151 | 74.4% | 133 | 88.1% |  |  |  |  |  |  | |
| Autumn and winter 2 | 52 | 25.6% | 49 | 94.2% | 2.21 [0.62 - 7.84] | 0.221 | 2.68 [0.78 - 9.18] | 0.118 | 2.77 [0.78 - 9.82] | 0.117 | |
| **Period of sampling** |  |  |  |  |  |  |  |  |  |  | |
| Period 1 | 109 | 53.7% | 99 | 90.8% |  |  |  |  |  |  | |
| Period 2 | 26 | 12.8% | 24 | 92.3% | 1.21 [0.24 - 6.07] | 0.815 | 1.09 [0.22 - 5.42] | 0.915 | 1.07 [0.20 - 5.82] | 0.939 | |
| Period 3 | 68 | 33.5% | 59 | 86.8% | 0.66 [0.24 - 1.80] | 0.421 | 0.55 [0.19 - 1.59] | 0.273 | 0.54 [0.17 - 1.74] | 0.302 | |
| **Education** |  |  |  |  |  |  |  |  |  |  | |
| In school | 74 | 36.5% | 68 | 91.9% |  |  |  |  |  |  | |
| Attended school before | 67 | 33.0% | 58 | 86.6% | 0.57 [0.19 - 1.69] | 0.312 | 0.63 [0.20 - 1.96] | 0.421 | 0.56 [0.17 - 1.78] | 0.324 | |
| Never attended school | 62 | 30.5% | 56 | 90.3% | 0.82 [0.23 - 2.97] | 0.767 | 1.03 [0.23 - 4.59] | 0.971 | 0.98 [0.21 - 4.54] | 0.979 | |
| **Gender** |  |  |  |  |  |  |  |  |  |  | |
| Female | 92 | 45.3% | 82 | 89.1% |  |  |  |  |  |  | |
| Male | 111 | 54.7% | 100 | 90.1% | 1.11 [0.47 - 2.62] | 0.815 | 1.08 [0.45 - 2.59] | 0.855 | 1.08 [0.45 - 2.62] | 0.863 | |
| **Caesarean section** |  |  |  |  |  |  |  |  |  |  | |
| No | 143 | 70.4% | 126 | 88.1% |  |  |  |  |  |  | |
| Yes | 60 | 29.6% | 56 | 93.3% | 1.89 [0.60 - 5.92] | 0.277 | 1.93 [0.63 - 5.90] | 0.248 | 1.93 [0.63 - 5.89] | 0.248 | |
| **Hospitalization *** |  |  |  |  |  |  |  |  |  |  | |
| Never | 110 | 54.2% | 97 | 88.2% |  |  |  |  |  |  | |
| Less than 1 day | 18 | 8.9% | 15 | 83.3% | 0.67 [0.18 - 2.55] | 0.559 | 0.78 [0.21 - 2.86] | 0.709 | 0.78 [0.21 - 2.88] | 0.712 | |
| Less than 1 week | 54 | 26.6% | 51 | 94.4% | 2.28 [0.62 - 8.44] | 0.219 | 2.75 [0.58 - 13.12] | 0.207 | 2.80 [0.61 - 12.89] | 0.189 | |
| 1 week or more | 21 | 10.3% | 19 | 90.5% | 1.27 [0.25 - 6.44] | 0.771 | 1.23 [0.23 - 6.56] | 0.809 | 1.25 [0.23 - 6.94] | 0.798 | |
| **Hospitalization 2 *** |  |  |  |  |  |  |  |  |  |  | |
| No | 110 | 54.2% | 97 | 88.2% |  |  |  |  |  |  | |
| Yes | 93 | 45.8% | 85 | 91.4% | 0.67 [0.18 - 2.55] | 0.559 | 0.78 [0.21 - 2.86] | 0.709 | 1.62 [0.57 - 4.58] | 0.369 | |
| **Hospitalization 3 *** |  |  |  |  |  |  |  |  |  |  | |
| Less than 2 days | 152 | 74.9% | 135 | 88.8% |  |  |  |  |  |  | |
| 2 days or more | 51 | 25.1% | 46 | 90.2% | 1.48 [0.47 - 4.65] | 0.503 | 1.46 [0.43 - 4.98] | 0.544 | 1.46 [0.43 - 4.99] | 0.545 | |
| **Hospitalization 4 *** |  |  |  |  |  |  |  |  |  |  | |
| Less than 1 day | 128 | 63.1% | 112 | 87.5% |  |  |  |  |  |  | |
| 1 day or more | 75 | 36.9% | 70 | 93.3% | 2.00 [0.69 - 5.79] | 0.203 | 2.20 [0.66 - 7.39] | 0.204 | 2.24 [0.67 - 7.47] | 0.191 | |
| **Hospitalization including caesarean section *** |  |  |  |  |  |  |  |  |  |  | |
| Less than 1 day | 107 | 52.7% | 93 | 86.9% |  |  |  |  |  |  | |
| 1 day or more | 96 | 47.3% | 89 | 92.7% | 1.91 [0.72 - 5.06] | 0.192 | 2.03 [0.70 - 5.84] | 0.192 | 2.05 [0.71 - 5.90] | 0.184 | |
| **Breastfeed after birth** |  |  |  |  |  |  |  |  |  |  | |
| Early breastfed | 176 | 86.7% | 155 | 88.1% |  |  |  |  |  |  | |
| Later | 27 | 13.3% | 27 | 100.0% |  | < 0.001 |  | < 0.001 | - | < 0.001 | |
| **Breastfeed after birth 2** |  |  |  |  |  |  |  |  |  |  | |
| Immediately | 161 | 79.3% | 141 | 87.6% |  |  |  |  |  |  | |
| Same day | 25 | 12.3% | 25 | 100.0% |  | < 0.001 |  | < 0.001 | - | < 0.001 | |
| More than 1 day | 15 | 7.4% | 14 | 93.3% | 1.99 [0.24 - 15.56] | 0.527 | 2.02 [0.19 - 21.73] | 0.564 | 2.02 [0.19 - 21.79] | 0.563 | |
| Unknown | 2 | 1.0% | 2 | 100.0% |  | < 0.001 |  | < 0.001 | - | < 0.001 | |
| **Exclusively breastfed 6 months** |  |  |  |  |  |  |  |  |  |  | |
| No | 200 | 98.5% | 180 | 90.0% |  |  |  |  |  |  | |
| Yes | 3 | 1.5% | 2 | 66.7% | 0.22 [0.02 - 2.58] | 0.231 | 0.23 [0.02 - 2.32] | 0.215 | 0.23 [0.02 - 2.32] | 0.212 | |
| **Exclusively breastfed 4 months** |  |  |  |  |  |  |  |  |  |  | |
| No | 137 | 67.5% | 126 | 92.0% |  |  |  |  |  |  | |
| Yes | 66 | 32.5% | 56 | 84.8% | 0.49 [0.19 - 1.27] | 0.143 | 0.49 [0.20 - 1.24] | 0.136 | 0.40 [0.12 - 1.32] | 0.134 | |
| **Still breastfed** |  |  |  |  |  |  |  |  |  |  | |
| No | 161 | 79.3% | 146 | 90.7% |  |  |  |  |  |  | |
| Yes | 42 | 20.7% | 36 | 85.7% | 0.62 [0.24 - 1.61] | 0.324 | 0.88 [0.24 - 3.22] | 0.845 | 0.88 [0.25 - 3.10] | 0.845 | |
| **Liquids first 3 days** |  |  |  |  |  |  |  |  |  |  | |
| Only mother milk | 92 | 45.3% | 80 | 87.0% |  |  |  |  |  |  | |
| Other milk | 107 | 52.7% | 98 | 91.6% | 1.63 [0.68 - 3.94] | 0.276 | 1.72 [0.74 - 4.03] | 0.212 | 1.92 [0.69 - 5.34] | 0.213 | |
| Infant formula | 4 | 2.0% | 4 | 100.0% |  | < 0.001 |  | < 0.001 |  | < 0.001 | |
| **Fully vaccinated **** |  |  |  |  |  |  |  |  |  |  | |
| Yes | 120 | 59.1% | 110 | 91.7% |  |  |  |  |  |  | |
| No | 83 | 40.9% | 72 | 86.7% | 0.60 [0.23 - 1.54] | 0.287 | 0.57 [0.22 - 1.48] | 0.246 | 0.57 [0.22 - 1.47] | 0.244 | |
| **BCG (1 dose at birth)** |  |  |  |  |  |  |  |  |  |  | |
| Yes | 194 | 95.6% | 173 | 89.2% |  |  |  |  |  |  | |
| No | 9 | 4.4% | 9 | 100.0% |  | < 0.001 |  | < 0.001 |  | < 0.001 | |
| **HepB (1 dose at birth)** |  |  |  |  |  |  |  |  |  |  | |
| Yes | 151 | 74.4% | 136 | 90.1% |  |  |  |  |  |  | |
| No | 12 | 5.9% | 11 | 91.7% | 1.12 [0.13 - 9.49] | 0.915 | 1.24 [0.16 - 9.76] | 0.84 | 1.23 [0.16 - 9.63] | 0.844 | |
| Unknown | 40 | 19.7% | 34 | 85.0% | 0.58 [0.21 - 1.58] | 0.287 | 0.45 [0.15 - 1.41] | 0.174 | 0.44 [0.14 - 1.43] | 0.173 | |
| **OPV (all doses at 4M)** |  |  |  |  |  |  |  |  |  |  | |
| Fully vaccinated | 165 | 81.3% | 153 | 92.7% |  |  |  |  |  |  | |
| Not (fully) vaccinated | 34 | 16.7% | 26 | 76.5% | 0.25 [0.09 - 0.68] | 0.007 | 0.20 [0.07 - 0.55] | 0.002 | 0.20 [0.07 - 0.55] | 0.002 | |
| Underage (< 4 months) | 4 | 2.0% | 3 | 75.0% | 0.24 [0.03 - 2.12] | 0.198 | 0.60 [0.05 - 7.84] | 0.700 | 0.61 [0.05 - 8.04] | 0.705 | |
| **Pentavalent (all doses 4M)** |  |  |  |  |  |  |  |  |  |  | |
| Fully vaccinated | 165 | 81.3% | 153 | 92.7% |  |  |  |  |  |  | |
| Not (fully) vaccinated | 34 | 16.7% | 26 | 76.5% | 0.25 [0.09 - 0.69] | 0.008 | 0.20 [0.07 - 0.57] | 0.003 | 0.20 [0.07 - 0.56] | 0.002 | |
| Underage (< 4 months) | 4 | 2.0% | 3 | 75.0% | 0.24 [0.03 - 2.12] | 0.198 | 0.61 [0.05 - 7.87] | 0.703 | 0.61 [0.05 - 8.11] | 0.710 | |
| **MMR (all doses at 18M)** |  |  |  |  |  |  |  |  |  |  | |
| Fully vaccinated | 121 | 59.6% | 113 | 93.4% |  |  |  |  |  |  | |
| Not (fully) vaccinated | 44 | 21.7% | 36 | 81.8% | 0.32 [0.11 - 0.92] | 0.035 | 0.25 [0.09 - 0.79] | 0.020 | 0.25 [0.08 - 0.74] | 0.014 | |
| Underage (< 18 months) | 38 | 18.7% | 33 | 86.8% | 0.47 [0.14 - 1.54] | 0.212 | 0.40 [0.03 - 4.90] | 0.473 | 0.39 [0.03 - 4.63] | 0.454 | |
| **Rotavirus** |  |  |  |  |  |  |  |  |  |  | |
| Yes | 80 | 39.4% | 72 | 90.0% |  |  |  |  |  |  | |
| No | 87 | 42.9% | 81 | 93.1% | 1.50 [0.50 - 4.53] | 0.474 | 1.47 [0.48 - 4.53] | 0.506 | 1.44 [0.49 - 4.21] | 0.510 | |
| Unknown | 36 | 17.7% | 29 | 80.6% | 0.46 [0.15 - 1.40] | 0.172 | 0.39 [0.11 - 1.36] | 0.144 | 0.37 [0.11 - 1.30] | 0.124 | |

* Hospitalization for any reasons after the baby was born. It is a common practice in Vietnam for children born via C-section to stay in the hospital for up to 3 days.

** Fully vaccinated as per the national EPI of Vietnam. taking age eligibility in consideration (see supplementary file 2).
