# Supplementary material for: Prevalence and determinants of faecal carriage of carbapenem- and third-generation cephalosporin-resistant Enterobacterales: a cross-sectional household survey in northern Vietnam
Source: Lancet Reg Health West Pac. 2025 Jan 13;54:101281. doi: 10.1016/j.lanwpc.2024.101281 (PMC11780954; doi:10.1016/j.lanwpc.2024.101281)
Supplement: Abstract_VN_clean [file mmc2.docx]

**Tóm tắt**

**Bối cảnh:** Kháng kháng sinh (KKS) là một đại dịch thầm lặng đã gây ra 1.27 triệu ca tử vong trong năm 2019, ảnh hưởng không đồng đều cho các quốc gia có thu nhập thấp và trung bình, tuy vậy tình trạng kháng kháng sinh của vi khuẩn trong vi hệ và các yếu tố quyết định liên quan đến việc cư trú của các vi khuẩn kháng thuốc chưa được báo cáo rộng rãi. Nghiên cứu cắt ngang này thực hiện trên các hộ gia đình nhằm mục đích xác định tỷ lệ hiện mang vi khuẩn Enterobacterales kháng carbapenem (CRE) và kháng cephalosporin thế hệ ba (C3GRE) tại một cộng đồng ở nông thôn thuộc tỉnh Hà Nam, phía bắc Việt Nam, cũng như để xem xét các yếu tố về nhân khẩu – xã hội, hành vi và môi trường liên quan tới việc mang vi khuẩn kháng kháng sinh. **Phương pháp**: Từ tháng 7/2018 đến tháng 4/2019, 1502 người từ 324 hộ gia đình đã tham gia vào nghiên cứu khảo sát này. Các mẫu phân được thu thập từ những người tham gia khảo sát và được nuôi cấy trực tiếp trên hai loại môi trường có bổ sung lần lượt các kháng sinh là meropenem để tìm CRE và ceftazidime để tìm C3GRE. Mô hình hồi quy logistic được sử dụng để tìm hiểu các yếu tố nguy cơ đối với việc mang CRE và C3GRE để so sánh với các chủng nhạy cảm. **Kết quả**: Tỷ lệ mang C3GRE và CRE lần lượt là 94,0% (Khoảng tin cậy (CI) 93,5% - 94.4%) và 1,9% (1,6% - 2,2%). Tỷ lệ hiện mang CRE quá thấp để xác định các yếu tố quyết định. Việc sử dụng kháng sinh trong tháng trước khi thu mẫu của người tham gia nghiên cứu (adjusted OR 1,22 [95% CI 0,45 – 3,31]) và biến cố về sức khỏe gần đây của họ (aOR 1,48 [0,34 – 6,51]) không liên quan đến việc mang C3GRE. Các yếu tố liên quan đến việc mang C3GRE là bao gồm thu nhập cao (OR 0,29 [0,12–0,74]), điều kiện vệ sinh kém (aOR 4,35 [1,07 – 17,43]), và việc tiêu thụ thịt bò thường xuyên (aOR 6,56 [2,16– 19,98]). Mối liên hệ mang tính bảo vệ giữa việc mang C3GNB và chăn nuôi được quan sát ở nhóm trẻ em dưới 5 tuổi (aOR 0,27 [0,09–0,84]). Với nhóm đối tượng từ 5 tuổi trở lên, tiêu thụ thịt gà có liên hệ với việc tăng khả năng mang C3GRE (aOR 3,45[1,45–8,22]), trong khi đó, thói quen ăn đậu phụ (aOR 0,32[0,14–0.74]) và thực phẩm lên men (aOR 0,55[0,31–0,96]) là yếu tố bảo vệ. **Phiên giải**: Trong bối cảnh có tỷ lệ cư trú C3GNE cao này, việc mang C3GRE không có mối liên hệ với việc sử dụng kháng sinh của các cá nhân, trong khi sự phơi nhiễm với môi trường, bao gồm thực phẩm và điều kiện vệ sinh, có liên quan đến việc mang C3GRE. Các nghiên cứu sâu hơn cần được thực hiện để hiểu cơ chế của các mối liên quan này. **Nguồn tài trợ**: Nghiên cứu này được hỗ trợ bởi khoản tài trợ nội bộ của Đơn vị nghiên cứu lâm sàng Đại học Oxford đến từ Chương trình tài trợ Châu Á, châu Phi của Wellcome Trust (2015 – 2022) tại Việt Nam (106680/Z/14/Z).

Disclaimer: This translation in Vietnamese was submitted by the authors and we reproduce it as supplied. It has not been peer reviewed. Our editorial processes have only been applied to the original abstract in English, which should serve as reference for this manuscript.
